# Supplementary material for: Connexin Genes Variants Associated with Non-Syndromic Hearing Impairment: A Systematic Review of the Global Burden
Source: Life (Basel). 2020 Oct 28;10(11):258. doi: 10.3390/life10110258 (PMC7693846; doi:10.3390/life10110258)
Supplement: Supplementary file 1 [file life-10-00258-s001.pdf]

Supplementary Materials

Connexin Genes Variants Associated with Non-Syndromic Hearing Impairment: A Systematic Review of the Global Burden

Table S1. Connexin genes primers sets used for targeted sequencing.

Gene	Forward/Reverse Primer	Frequency
GJB2	TTGGTGTTTGCTCAGGAAGA/GGCCTACAGGGGTTTCAAAT	8
GJB2	TCTTTTCCAGAGCAAACCGC/GACACGAAGATCAGCTGCAG	7
GJB2	CCTATGACAAACTAAGTTGGTTC/GACAGCTGAGCACGGGTTGCCTC	4
GJB2	GCTTACCCAGACTCAGAGAAG/CTACAGGGGTTTCAAATGGTTGC	4
GJB2	TCCGTAACTTTCCCAGTCTCCGAGGGAAGAGG/CCCAAGGACGTGTGTTGGTCCAGCCCC	4
GJB2	CCAGGCTGCAAGAACGTGTG/GGGCAATGCGTTAAACTGGC	3
GJB2	CTCCCTGTTCTGTCCTAGCT/CTCATCCCTCTCATGCTGTC	3
GJB2	TCTTTTCCAGAGCAAACCGC/GGGCAATGCGTTAAACTGGC	3
GJB2	AGTGGCCATGCACGTGGCCTA/TGATCTCCTCGATGTCCTTAA	2
GJB2	CCAGGCTGCAAGAACGTGT/ACAGCTGAGCACGGGTTG	2
GJB2	CCGGGAAGCTCTGAGGAC/GCAACCGCTCTGGGTCTC	2
GJB2	CTCCCTGTTCTGTCCTAGCT/AGCTGAGCACGGGTTGCCTCA	2
GJB2	CTGTCCTAGCTATGTTCC/TCAGCACGGGTTGCCTCAATC	2
GJB2	GAGGTTGTGTAAGAGTTGGTGTT/TCTTCTCATGTCTCCGGTAG	2
GJB2	GCCGCCCCCTCCGTAACTTTC/CGTGTGTTGGTCCAGCCCCCC	2
GJB2	GCTTACCCAGACTCAGAGAAG/CTTAATCTAACAACTGGGCAATGC	2
GJB2	GTCTCCCTGTTCTGTCCTA/TCTAACAACTGGGCAATG	2
GJB2	TCAAAGGAACTAGGAGATCGG/CAAGGACGTGTGTTGGTCCAG	2
GJB2	TCTTTTCCAGAGCAAACCGC/CTGGGCAATGCGTTAAACTGG	2
GJB2	TCTTTTCCAGAGCAAACCGCC/TGAGCACGGGTTGCCTCATC	2
GJB2	TGCTTACCCAGACTCAGAGAA/GACTGAGCCTTGACAGCTGAG	2
GJB2	AAGGGGCTGGCTCTTCA/GCACTTGGCCTGGGTAA	1
GJB2	AAGTCTCCCTGTTCTGTCCTAG/CCTCATCCCTCTCATGCTGTC	1
GJB2	ACACGTTCAAGAGGGTTTGG/CCTCATCCCTCTCATGCT	1
GJB2	ACACGTTCAAGAGGGTTTGG/GGGAAATGCTAGCGACTGAG	1
GJB2	ACTCATGGGGGCTCAAAGGA/GCAACCGCTCTGGGTCTCGCGGTCCCT	1
GJB2	AGAAGAAGAGGAAGTTCATCA/CCTCTCATGCTGTCTATTTC	1
GJB2	AGAAGAAGAGGAAGTTCATCAAGG/CTGAGGCCTACAGGGGTTTC	1
GJB2	AGACTCAGAGAAGTCTCCCTG/GACACGAAGATCAGCTGCAG	1
GJB2	AGACTCAGAGAAGTCTCCCTG/GCCAGTTTAACGCATTGCCC	1
GJB2	AGAGTGGTGTTTGCTCAGGA/GACTGAGCCTTGACAGCTGA	1

GJB2	AGCAAACCGCCCAGAGTAGAAG/TGATCTTCGTGTCCACGCCAG	1
GJB2	AGGCCGACTTTGTCTGCAACA/GTGGGCCGGGACACAAAG	1
GJB2	AGTCTCCCTGTTCTGTCCTA/TGAGCACGGGTTGCCTCATC	1
GJB2	AGTGCCTTTCAGCTAACGA/GTGGCATCTGGAGTTTCACC	1
GJB2	CACGCTGCAGACGATCC/GGTGGAGTGTTTGTTCAC	1
GJB2	CACTTTCCCAAGGCCTCTTCCAC/GTACGTCCACCACAGCGAC	1
GJB2	CAGTCTCCGAGGGAAGAGG/AAGGACGTGTGTTGGTCCAG	1
GJB2	CAGTCTCCGAGGGAAGAGG/GCAACCGCTCTGGGTCTC	1
GJB2	CCACGCCAGCGCTCCTAGTG/GAAGATGCTGCTGCTTGTGTAGG	1
GJB2	CCAGCATGACCTTTACCAG/ATCACTTGAATAAGAAGCCATTTG	1
GJB2	CCAGGCTGCAAGAACGTGTG/GACACGAAGATCAGCTGCAG	1
GJB2	CCAGGCTGCAAGAACGTGTG/RTGAGCACGGGTTGCCTCATC	1
GJB2	CCAGGCTGCAAGAACGTGTG/TGAGCACGGGTTGCCTCATC	1
GJB2	CCCTCCGTAACTTTCCCAGT/CCAAGGACGTGTGTTGGTC	1
GJB2	CCCTCCGTAACTTTCCCAGT/GCAACCGCTCTGGGTCTC	1
GJB2	CCTATGACAAACTAAGTTGGTTC/GCCTCATCCCTCTCATGCTGTC	1
GJB2	CCTCCGTAACTTTCCCAGT/AAGGACGTGTGTTGGTCCAG	1
GJB2	CCTGTGTTGTGTGCATTCGTC/CTCATCCCTCTCATGCTGTC	1
GJB2	CGAAGCCGCCTTCATGTACG/TTAGGGGAGCAGAGCTCCAT	1
GJB2	CGCACTATGCGGAGTACAGA/GGTGGCAGTGGGTCAAGTAG	1
GJB2	CGCGCTCCTCTCCCCGACT/TCCTTTGCAGCCACAACGAGGAT	1
GJB2	CGGAGACATGAGAAGAAGAGG/GATCTCCTCGATGTCCTTAA	1
GJB2	CGTAACTTTCCCAGTCTCCGAGGGAAGAGG/CCCAAGGACGTGTGTTGGTCCAGCC	1
GJB2	CGTAGCACACGTTCTTGCAGCCTG/CGATGCGGACCTTCTGGGTTTTG	1
GJB2	CGTCTTTTCCAGAGCAAACCG/AGCTCCATTGTGGCATCTGG	1
GJB2	CGTTCGTTCGGATTGGTGAG/CAGAAACGCCCGCTCCAGAA	1
GJB2	CTCATGGGGGCTCAAAGGAACTAGGAGATCGG/GGGGCTGGACCAACACACGTCCTTGGG	1
GJB2	CTCATGTCTCCGGTAGGCCAC/GCAGCATCTTCTTCCGGGT	1
GJB2	CTGGTGCTACGATCACTAC/TTCCAGACACTGCAATCATG	1
GJB2	CTTTTCCAGAGCAAACCGC/GGGCAATGCGTTAAACTGGC	1
GJB2	FTCTTTTCCAGAGCAAACCGCC/GACACGAAGATCAGCTGCAG	1
GJB2	GAAGTAGTGATCGTAGCACACGTTCTTGCA	1
GJB2	GAAGTCTCCCTGTTCTGTCCT/TCTAACAACTGGGCAATGC	1
GJB2	GAGCCTTCGATGCGGACCTT/TCATCCCTCTCATGCTGTC	1
GJB2	GCATTCGTCTTTTCCAGAGC/GGCCTACAGGGGTTTCAAAT	1
GJB2	GCATTCGTCTTTTCCAGAGCA/GAGCCTTCGATGCGGACCTT	1
GJB2	GCCAGGCTGCAAGAACGTGT/GGAGAAGCCGTCGTACATGA	1
GJB2	GCGCAAGCTTTATGGATTGGGGCACGCT/GCGCGGATCCCTAACTGGCTTTTTTGAC	1
GJB2	GCTTACCCAGACTCAGAGAAG/TGAGCACGGGTTGCCTCATC	1

GJB2	GGGCAATGCGTTAAACTGGC/CCAGGCTGCAAGAACGTGTG	1
GJB2	GGGGAGATGAGCAGGCCGAC/CGGCTGGTGAAGTGCAACGC	1
GJB2	GGGGCTCAAAGGAACTAGGA/AAGGACGTGTGTTGGTCC	1
GJB2	GGGGGCACTTGGGGAACTCA/GCAGAAACGCCCGCTCCAGAA	1
GJB2	GGTGAGGTTFTFTAAGAGTTGG/CTACGGTGTTACTCGAGACGA	1
GJB2	GGTGAGGTTGTGTAAGAGTTGG/TAGCGACTGAGCCTTGACAG	1
GJB2	GTCTCCCTGTTCTGTCCTAG/CTTCGAAGATGACCCGGAAG	1
GJB2	GTGCATTCGTCTTTTCCAGAGCA/TTGACAGCTGAGCACGGGTTG	1
GJB2	GTGGCCTACCGGAGACAT/CACTCTTTATCTCCCCCTTG	1
GJB2	GTGTGCATTCGTCTTTTCCAG/GCGACTGAGCCTTGACA	1
GJB2	GTGTTGTGTGCATTCGTCTTTTC/ACCTTCTGGGTTTTGATCTCCTC	1
GJB2	TACGATGGTTTTTCCTCTAATTCT/TTGCATAACTTAGTGAACTCAGAG	1
GJB2	TATGTCATGTACGACGGCT/TCTAACAACTGGGCAATGC	1
GJB2	TATGTTCCTGTGTTGTGTGC/CCTTCTGGGTTTTGATCTCC	1
GJB2	TCAAGGGGGAGATAAAGAGT/TGAGCACGGGTTGCCTCATC	1
GJB2	TCAAGGGGGAGATAAAGAGTG/TGAGCACGGGTTGCCTCATC	1
GJB2	TCAGAGAAGTCTCCCTGTTCTGTCC/TGAGGCCTACAGGGGTTTCAA	1
GJB2	TCCGTAACTTCCCAGTCTCCGAGGGAAGAGG/CCCAAGGACGTGTGTTGGTCCAGCCCC	1
GJB2	TCCGTAACTTTCCCAGTCTCCGAGGGAAGAG/CCCAAGGACGTGTGTTGGTCCAGCCC	1
GJB2	TCCGTAACTTTCCCAGTCTCCGAGGGAAGAGG/CCCAAGGACGTGTGTTGGTCCAGCCC	1
GJB2	TCCTGGGGGTGTGA/CCTGGGGGGTGTG	1
GJB2	TCGGCCCCAGTGGTACAG/CTGGGCAATGCGTTAAACTGG	1
GJB2	TCTTTTCCAGAGCAAACCGC/GTTGGGAAATGCTAGCGACT	1
GJB2	TCTTTTCCAGAGCAAACCGC/TGGGCAATGCGTTAAACTGGC	1
GJB2	TCTTTTCCAGAGCAAACCGC/TTGCCTCATCCCTCTCATGCTGT	1
GJB2	TCTTTTCCAGAGCAAACCGC3/CTGGGCAATGCGTTAAACTGG	1
GJB2	TCTTTTCCAGAGCAAACCGCC/CACGTGCATGGCCAGTAG	1
GJB2	TGCATCACCTCACATAGGTTA/TATTGGATACTTGAATCTGCTG	1
GJB2	TGCTTACCCAGACTCAGAGAA/CGACTGAGCCTTGACAGCTGA	1
GJB2	TGCTTGCTTACCCAGACTCA/CCTCATCCCTCTCATGCTGT	1
GJB2	TGGGGAACTCATGGGGGCTCAAAG/AGGTTCCTGGCCGGGCAGTCC	1
GJB2	TGGTGGCCATGCATGTGGCCTAC/CAGAAGTCTCCTTATGACGCAGC	1
GJB2	TGGTGTTTGCTCAGGAAGAG/TTGTGTAGGTCCACCACAGG	1
GJB2	TGTGCATTCGTCTTTTCC/CAGATCTTTCCAATGCTGGT	1
GJB2	TGTGCATTCGTCTTTTCCAG/CAGATCTTTCCAATGCTGGT	1
GJB2	TGTGCATTCGTCTTTTCCAG/GGGAAATGCTAGCGACTGAG	1
GJB2	TTGGGGCACGCTGCAGACGATCCTGGGGAG	1
GJB2	TTGGTGTTTGCTCAGGAAGA/GGCATCTGGAGTTTCACCTG	1
GJB2	TTTGCTCAGGAAGAGATTTAAGCA/GGGTTTTGATCTCCTCGATGTCC	1

GJB6	AGACTAGCAGGGCAGGGAGT/GGTTGGTATTGCCTTCTGGA	1
GJB6	GACTAGCAGGGCAGGGAGTT/CTCTTCTCTCCTCGCCTGAA	1
GJB6	TCCGTAACTTTCCCAGTCTCCGAGGGA AGAGG/CCCAAGGACGTGTGTTGGTCCAGCCCC	1
GJB6	GTCTGTAATATCACCGTGTCAC/CTCTTCAGGCTACAGAAGGAAC	1
GJB6	GGGCAGGGAGTTGAAGTTG/ACTCTTCAGGCTACAGAAGGAAC	1
GJB6	CTTTGCCCACTTTTGTCTGT/GACCCCTCTATCCGAACCTT	1
GJB6	TTTAGGGCATGATTGGGGTGATT/CACCATGCGTAGCCTTAACCATTTT	1
GJB6	CTCCTTTAGGGCATGATTGG/CCATGCGTAGCCTTAACCAT	1
GJB6	GACCACTTTTTCCCGGTGT/AGGTTGGTATTGCCTTCTGG	1
GJB6	CCTCCAGCTGATCTTCGTCT/GCAGCAGGTAGCACAACTCT	1
GJB6	TGCCCACCCCCCAAGTAGAG/TTTCGGTTTCATTCATTTTCCCTATT	1
GJB6	GCCCCAACCTTGTTGACTGC/GTTGGTATTGCCTTCTGGAG	1
GJB6	GGCAGGGAGTTGAAGTTGTA/ACGTTGTGTATGAATGGAGCA	1
GJB6	GAAAGGAAGGTCGGGCAAGG/CACAATCAAACCTCACTGCCATCTT	1
GJB6	GTTGCTTGTGCTTTTGGTGTCAT/AGCCCAGAAACAAACCCTTACATA	1
GJB3	GCAGGTAGGCAAGCCCCCACCAG/GCCACACTGCCCTGCATTTCCC	1
GJB3	ATTCTCTCAGGTAGGCACGG/TGGTGTTGCAGTCAAAGTCC	1
GJB3	CTATTCATTCATACGATGG/TCACTCAGCCCCTGTAGGAC	1
GJB3	CCCAGTTCCCAGTGTCAAA/TACGACAACGCAGGCAAGA	1
GJB3	TACGATGGTTTTTCCTCTAATTCT/TTGCATAACTTAGTGAACTCAGAG	1
GJB3	TATACGTGGTGGCTGCAGAG/CTGCGTTGTCGTACAGCTTG	1
GJB3	TAGGTCGGGCAATGTAGCA/GAACTCAGAACACTGCCTGGT	1
GJB3	CTCGCTGCTGGTCATCCT/CATATTGAAGCCATGCCAGA	1
GJB3	TTCCTCTACCTGCTGCACAC/GGCAGATGAGGTAGCAGAGC	1
GJB3	AGAGGGTCGTTGTGAGTATTG/AGAGGCGGATGTTGGAGATG	1
GJB3	TGCAGCTTGGGAGGAATAAC/CCCCTGTAGGACCTCTCCAC	1
GJB3	TGCTACGACAACTACTTCCCC/GGCGCCCACCATGAAGTAG	1
GJA1	GAAATACGTGAAACCGTT GG/ CCT GGT GCA CTT TCT ACA GC	1
GJA1	TGCGGTCTACACCTGCAAGA/ACCAAGGACACCACCAGCAT	1
GJA1	CGTGAAACCGTTGGTAGTATTT/CCTCCACCGGATCAAAATTA	1
GJA1	GGAGTTCAATCACCTTGGCGT/TCTTGATGCTTTCAAGCCTGT	1
GJA1	TTTGCAATCTGTGATCCTTGA/CCTGGTGCACTTTCTACAGC	1
GJB4	TCAATCGCACCAGCATTAAG/GGGGGACCTGTTGATCTTATC	1
GJB4	GCATTAAGGGTGCCCATCTC/TTTTCCTGGGTGGCCTCAT	1
GJC3	GCTCCCTCTGAAGGACAGTG /GGGAGGAGATCATCAGGACA	1
GJC3	TGGGTACGCACTGTGAAAAA/AGCTCCTCCTTGGACAGGAT	1

Table S2. *GJB2* variants with no clinical significance data from the three databases used.

Variant	Allele frequency [Reference]
IVS1+12G>A (NM_004004.6:c.+12G>A)	4/126 [1]; 1/1220 [2]
c.IVS1+27G>C	1/152 [3]
c.-3179G>A	1/218 [2]
c.23+G>A	8/90 [4]
51del12insA	2/72 [5]; 2/150 [6]
c.684C>A	1/300 [7]; 1/ 546 [8]; 1/209 [9]; 1/1220 [2]
p.His100ArgfsTer14/+ (c.299- 300delAT)	1/200 [10]
delE119 (c.355-357delGAG)	2/67[11]; 1/40 [12]; 3/150 [13]; 2/32 [14]
682C>T	1/114 [11]; 1/1220 [2]
c.964c>T	5/36 [15]
c.-31T>C	1/418 [9]
c.765C>T	3/113 [16]
p.P87del	1/112 [17]

Table S3. Eight commonly reported connexin 26 gene (*GJB2*) variants.

Country	p.Gly12 ValfsTer2 (c.35delG) rs80338939	p.M34T (c.101T>C) rs35887622	p.L79Cfs (c.235delC) rs80338943	p.V37I (c.109G>A) rs72474224	p.H100RfsTer14 (c.299_300delAT) rs111033204	p.W24* (c.71G>A) rs104894396	p.L56Rfs (c.167delT) rs80338942	p.R143W (c.167delT) rs80338948	Reference
Japan								2/2	[18]
Japan			10/70	1/70				4/70	[19]
Morocco	61/162			3/162					[20]
Ghana								64/442	[21]
Israel	2/8								[22]
Malaysia				2/66		4/66			[23]
Syria	9/70								[24]
Syria	36/100	2/100					5/100		[25]
Saudi Arabia	14/118			1/118		2/118			[26]
Kuwait	21/200								[27]
Spain	4/4								[28]
Spain	5/29					23/29			[29]
Algeria	55/62								[30]
Algeria	36/50			3/50			2/50		[31]
Algeria	27/232								[32]
Venezuela	5/84								[33]
Greece	7/58								[34]
Turkey	11/58				2/58				[35]
Iran	12/90				2/90				[4]
Bangladesh						19/134			[36]
Morocco	107/304			10/304					[37]
Turkey	25/62								[38]
India						2/116			[5]
Yakutia	51/786			14/786			1/786		[39]
Turkey	96/470								[40]
USA	6/6								[41]
Brazil	74/600	8/600	1/600	1/600		1/600	2/600		[7]
Brazil	3/4								[42]
Slovenia	15/138								[43]
Turkey	2/40				2/40				[44]
Iran	547/4644		1/4644	2/4644	8/4644	31/4644	12/4644	7/4644	[45]
Germany	110/138	1/138		2/138					[46]

Tunisia	44/204								[47]
Brazil	14/66			1/66					[48]
Brazil	14/66			1/66					[49]
Tunisia	6/6								[50]
India	27/116					10/116			[51]
Russia	527/1410	12/1410	14/1410	4/1410		3/1410	12/1410		[52]
Russia	86/222	2/222	1/111						[53]
Germany	8/50								[54]
Iran	7/348		1/348						[55]
Iran	10/1016		2/1016	2/1016	1/1016	4/1016	2/1016	1/1016	[56]
Hungary	2/326								[57]
Romania	3/692								[57]
Ashkenazi							9/372		[57]
Turkey	8/154								[58]
Ghana								44/58	[59]
Pakistan	2/140					2/140			[60]
Germany	13/1012	1/1012					1/1012		[61]
Italy	164/752	5/752					2/752		[62]
Sweden	44/158	1/158						2/158	[63]
São Tomé and Príncipe		2/272							[64]
Guatemala		1/266							[65]
Brazil	8/154	1/154							[66]
China			27/228	84/228	3/228				[67]
China			7/1238						[68]
Iran	10/68		2/68			1/68			[69]
USA	28/146		3/146	47/146	2/146	2/146	1/146	2/146	[70]
USA	20/542	7/542	2/542	40/542	2/542	3/542	2/542	2/542	[71]
China			42/230	6/230	3/230				[72]
China			41/230						[73]
China					1/70				[74]
China			1/58						[75]
China				2/200					[10]
China			118/1402	101/1402					[76]
China	2/11600		356/11600	597/11600	45/11600				[77]
China			6/50	11/50	4/50				[78]
China	9/160		47/160		7/160				[79]
Italy	9/258	1/258				1/258	1/258		[80]
Korea			10/102	1/102					[81]

Brazil	24/98			2/98					[82]
Chile	28/226								[83]
Italy	310/12395	54/12395							[84]
USA	76/98						11		[85]
Brazil	12/154								[86]
Belgium	67/554			5/554			11/554	3/554	[87]
Australia	50/104	5/104		8/104		3/104	1/104	1/104	[88]
China	12/4126		510/4126	200/4126	97/4126			4/4126	[89]
China			721/6008						[90]
China			278/2348	88/2348	19/2348				[91]
Argentina	16/92	1/92							[92]
Argentina	85/952	13/952		7/952			11/952	6/952	[93]
Argentina	46/504	7/504		2/504			2/504	1/504	[94]
Belarus	407/782		5/782				6/782		[95]
Iran	1/2								[96]
Portugal	75/234			1/234		2/234			[97]
Netherlands	8/18								[98]
Brazil	12/154								[99]
Mexico	12/152						2/152		[100]
Brazil	84/414	6/414		4/414					2006
Romania	11/250					8/250			[101]
Romania	11/700								[102]
Turkey	2/2								[103]
Egypt	11/102								[104]
Egypt	13/72								[105]
USA	27/136	1/136	2/136	2/136			3/136		[106]
Iran	49/266								[107]
Brazil	8/202								[108]
Italy	83/120								[109]
Spain	18/120								[109]
Kuwait	18/200								[110]
France	10/376	12/376							[111]
Peru	16/266		1/266	2/266			3/266	21/266	[112]
USA	29								2004
Austria	2/90								[113]
Austria	50/204					3/204			[11]
Austria	20/86					2/86			[12]
Germany	38/268				2/268				[114]
China				15/1300					[115]

China			181/1390		63/1390			5/1390	[116]
Morocco	50/232								[117]
Egypt	14/206								[118]
Argentina	11/712								[119]
Argentina	26/188			2/188			2/188	4/188	[120]
Denmark	9/980	5/980		2/980					[121]
Italy	23/358								[122]
Germany	2/4								[123]
Iran	144/440						9/440		[124]
UK	9139/18404	9112/18404							[125]
Ghana								110/758	[126]
China			2709/142417						[127]
Iran	7/100								[128]
Japan			37/138					15/138	[129]
Sweden	34/4104								[130]
Turkey	101/126				1/126		1/126	1/126	[131]
Canada	12/114		1/114				2		[132]
UK	2/346	14/346							[133]
China			7/598	90/598	1/598				[134]
China			6/68	1/68	1/68				[135]
China			32/7728	38/7728	7/7728			5/7728	[136]
China			2/600	17/600	1/600				[137]
China	35/80								[138]
Taiwan			38/648	58/648	5/648				[139]
Greece	54/214								[140]
Saudi Arabia	2/160								[141]
Eastern Azarbaijan	27/258								[142]
Austria	44/408						1/408		[143]
China	1/352		53/352	23/352	6/352				[144]
China	1/120		12/120		2/120				[145]
China				3/6					[146]
China			26/310	28/310	2/310				[147]
China			1/2		1/2				[148]
India						28/172			[149]
Korea			1/56		1/56			2/56	[150]
Germany	44/674								[151]
Turkey	48/186					3/186			[152]
Turkey	32/130								[153]

Iran	29/206			1/206	1/206	2/206	3/206		[154]
Hungary	60/478	4/478		3/478		4/478			[155]
USA	137/225	22/225	8/225	23/225	1/225	2/225	22/225	1/225	[156]
USA	21/48	4/48					9/48	1/48	[157]
Qatar	9/252								[1]
India	21/100					8/100			[158]
Russia	51/100								[159]
Korea				1/6					[160]
Korea				2/192					[161]
Korea				2/4					[162]
Korea			16/1176	4/1176	3/1176			11/1176	[163]
Korea			3/320	1/320				2/320	[164]
Korea			1/206	2/206				1/206	[165]
Greece	1/3								[166]
Iran	25/280								[167]
Iran	30/68		1/68			1/68			[168]
Japan			7/78						[169]
Thailand			3/34			1/34			[170]
Germany	51/684								[171]
Portugal		2/4							[172]
France	172/1380	44/1380							[173]
Korea			4/44						[174]
Israel	8/14						4/14		[175]
Israel	9/54						43/54		[176]
China			10/432						[176]
China			60/250		14/250				[177]
China			2/2						[178]
USA	25/320	3/320	2/320	2/320					[179]
USA	14/308	3/308	1/302	7/302	3/302	1/302	3/302		[180]
China			9/254	2/254					[181]
China	2/236		48/236	5/236	10/236				[182]
China			2/6		1/6				[183]
Mexico	36/176	1/136							[184]
Finland	26/142	3/142		1/142					[185]
China			39/312	7/312	10/312				[186]
Jordan	40/304								[187]
Syria	8/140						1/140		[188]
Iran	3/124					2/124			[189]
Iran	20/458								[190]

Iran	15/228			1/228		1/228			[191]
Iran	17/154								[192]
India	1/90					1/90		1/90	[193]
China			23/270						[194]
India				2/1060		174/1060		1/1060	[195]
Brazil	41/162								[196]
France	287/414		1/414			4/414	6/414		[197]
France	62/192						2/192		[198]
Mexico	4/4								[199]
Tunisia	10/70								[200]
Portugal						2/12			[201]
Croatia	37/126		1/126					1/126	[202]
Croatia	3/18								[203]
Jordan	22/136								[204]
Gado Bravo	5/88					3/88			[205]
Queimadas	21/152								[205]
USA				2/16					[206]
Rhode Island New	9/84						2/84		[207]
Slovakia	9/108			1/108		25/108			[208]
Slovakia	122/546	6/546		6/546		15/546	3/546		[8]
India	1/30					6/30			[209]
Mauritania	5/278								[210]
Egypt	27/194								[211]
Brazil	6/142								[212]
Italy	40/106								[213]
Iran	25/262		4/262						[214]
Iran	11/336					1/336			[215]
India						7/54			[216]
Cyprus	49/60						1/60		[217]
Japan			96/2454	61/2454	8/2454			8/2454	[218]
Brazil	43/72			3/72					[219]
Iran	1/2								[220]
Iran	1/2								[221]
Italy	136/200	2/200					3/200	1/200	[222]
UK	4/4								[223]
France	3/10								[224]
USA		11/1042	1/1042	5/1042	1/102		24/1042	4/1042	[225]
Korea	1/294		15/294		1/294			1/294	[226]
Ecuador	2/222							3/222	[227]

China			52/180		6/180				[228]
Athens	1/2								[229]
Athens	4/6								[230]
Iran	8/84								[231]
Brazil	14/66			1/66					[48]
Italy	32/308	2/308		1/308			1/308		[232]
Poland	0	17/466	3/466	17/466			7/466		[233]
Bulgaria	45/102					1/102			[234]
Russia	18/152		6/152						[3]
USA	86/418	5/418		2/418					[235]
Italy				1/8					[236]
China	2/2328		290/2328	210/2328	55/2328				[237]
Romania	32/90				1/90	2/90			[238]
Austria	26/42								[239]
Italy	3/78	1/78		1/78					[240]
Iran	39/126								[241]
Tunisia	2/4								[242]
Tunisia	82/262		2/262	2/262					[243]
Tunisia				2/2					[244]
UK	3/102					2/102			[245]
USA	2/288	2/288		3/288					[246]
Colombia	1/10								[247]
France	31/60	5/60	1/60	3/60		1/60		1/60	[16]
Iran	10/106					1/10/6			[248]
Iran	7/80					1/80			[249]
Pakistan						43/340			[250]
Italy	147/204	3/204					12/204		[251]
Sicily	35/128								[252]
USA	1/218	2/218		12/218					[253]
Pakistan						9/392			[254]
Netherlands	24/444	1/444		1/444				1/444	[255]
USA	28/95	2/95	2/95	10/95			1/95		[256]
Czech	10/13								[257]
Czech	113/312					13/312			[258]
Pakistan	19/125					52/125			[259]
Palestin	11/96		2/96				7/96		[260]
USA	13/418	1/418		2/418			3/418	1/418	[9]
China			9/20		1/20				[261]
Korea			31/842		6/842			4/842	[262]

Norway	82/201					2/201			[263]
Austria	56/500	9/500	5/500	10/500		6/500	4/500	1/500	[264]
India	6/632			26/632		38/632			[265,266]
UK	22/48								[266]
Hungary						9/1982			[267]
Indonesia				4/240					[268]
Egypt	24/222			3/222					[269]
Israel	31/150						21/150		[6]
Brazil	47/360	3/360		2/360		2/360	1/360		[270]
Colombia	38/224	3/224					9/224		[271]
USA	120/1220	19/1220	1/1220	38/1220			6/1220	1/1220	[2]
Japan			8/1018	9/1018				/10181	[272]
Turkey	22/188						1/188		[273]
Turkey	33/42						1/42		[274]
Turkey	141/742				1/742	2/742	1/742	1/742	[275]
Mongolia			16/1068	6/1068	4/1068			1/1068	[276]
China			27/200						[277]
China	1/256		28/256		2/256				[278]
United Arab Emirates	14/100								[279]
Hungary	104/NA								2002
Japan	2/2686		142/2686	47/2686	11/2686			18/2686	[280]
Turkey	28/120					3/120			[281]
China			8/34		1/34				[282]
China			22/338		12/338				[283]
China			64/672		18/672				[284]
Thailand	0		10/332	37/332		3/332			[17]
China	2/1316		154/1316	23/1316	33/1316				[285]
USA	6/20								[286]
Austria	11/36	2/36		3/36			1/36		[287]
USA	71/648	28/648	2/648	11/648		1/648	24/648	1/648	[288]
Taiwan			38/840	129/840	8/840			1/840	[289]
Taiwan			25/2034	184/2034					[290]
China			1/4						[291]
China			47/470		6/470				[292]
Taiwan			12/506						[293]
UK						10/354			[294]
China	5/424		164/424		24/424				[295]
China			1/228	2/228	1/228				[296]

Malaysia		1/64		5/64		3/64			[297]
Iran	105/836		1/836	2/836				2/386	[298]
China	4/2134		298/2134	189/2134	27/2134			2/2134	[299]
China			188/2402	34/2402	35/2402				[300]
Azarbaijan	14/258		2/258						[301]
Ghana								37/40	[302]
India						30/430			[303]
Algeria	27/232								[304]
USA	54/238			1/238			3/238		[305]
Turkey	57/346								[306]
Malaysia				1/62		3/62			[307]
USA	301/434	31/434		18/434			22/434		[308]
Turkey	1/14	1/14	1/14				1/14		[309]
Turkey	4/24								[310]
Germany	102/670			4/670	1/670	3/670	3/670		[311]
Slovenia	115/436			5/436		4/436			[312]
Turkey	13/296								[313]
Italy	199/277	15/277		1/277		3/277	3/277	3/277	[314]
Russia	527/1410	12/1410	9/1410	4/1410		3/1410			[315]
Turkey	76/542		4/542			3/542		1/542	[13]
Balkan Gypsies						28/1206			[316]
Russia	29/54		1/54				1/54		[14]
Iran								3/76	[317]
Iran	15/200								[318]
USA	178/386	11/386	3/386	11/386		1/386	23/386	1/386	[319]
Korea			2/290		2/290				[320]
Brazil	12/77								[321]
USA	2/26	14/26							[322]
Brazil	43/370								[323]
Brazil	40/1200	2/1200		2/1200		1/1200			[324]
Iran	26/332				1/332	1/332			[325]
Iran	18/100				2/100				[326]
China			10/30						[327]
USA	7/38	2/38							[328]
Finland	14/184	3/184						2/184	[329]
Portugal	3/13	11/13							[330]
Portugal	19/178	6/178							[331]
China	2/1076		73/1076	24/1076	46/1076				[332]
China	24/4796		292/4796		57/4796			4/4796	[333]

China	1/566		29/566	10/566	6/566				[334]
China	2/968		46/968	10/968	20/968			2/968	[335]
Turkey	2/2								[336]
Egypt	24/102								[337]
Mongolia	6/378		8/378	4/378	4/378				[338]
Turkey	15/112					2/112			[339]
Iran	27/100							5	[340]
Brazil	16/100								[341]
Spain	3/76								[342]
Spain	5/8								[343]
Toronto (Canada)						4/4	8		[344]
Vietnam			2/174	54/174	1/174				[345]
China	2/142		5/142		2/142				[346]
Korea			26/4144	28/4144	3/4144				[347]
China	3/12		3/12						[348]
Iran								2/4	[349]
Iran	10/158								[350]
China	24/2114		340/2114		40/2114			3/2114	[351]
Mexico	7/156	1/156						1/156	[352]
China					3/60				[353]
Greece	111								[354]
Slovak Republic	13					1			[355]
China	2		105	29	25				[356]
Japan			1		1				[357]
USA	33	2					9		[358]
Russia	51								[359]
Korea			8	10					[360]
USA	32			1			2	1	[361]
USA	58								[362]
Japan			16/38	4/38				4/38	[363]
China			2/6		2/6				[364]
Romania	98/358					13/358			[365]
Romania	50/150					8/150			[366]
China	4/324		76/324	13/324	12/324				[367]
Slovenia	3/64								[368]
China			104/1268		10/1268				[369]
USA	47/64			2/64					[370]
China			26/171		2/171				[371]
China	1/420		67/420	1/420	1/420				[372]

China			10/86		2/86				[373]
Finland		19/30		2/30				1/30	[374]
France	14/512								[375]
China			108/1070	12/1070	27/1070			3/1070	[376]
Portugal	84/528	7/528		5/528		5/528	1/528		[377]
Mexico	3/22								[378]
Slovakia	9/108			1/108		25/108			[379]
Morocco	13/278								[380]
India						7/27			[381]
Italy	130/178								[382]
Iran						10/200	2/200		[383]
Hungary	69/1892								[384]
Iran	167/1328					11/1328	3/1328	1/1328	[385]
Cyprus	19/60								[386]
Italy	34/2080	1/2080		2/2080			8/2080		[387]
Poland	2/6								[388]
Japan	3/30	1/30							[389]
India			2/912			52/912	1/912		[390]
India						2/2			[391]
France	14/74	1/74		2/74					[392]
Greece	139/510					2/510			[393]
China	6/760		55/760	10/760	26/760				[394]
Brazil	15/82								[395]
Russia			5/440	2/440	2/440				[396]
Germany	313/NA								[397]
Italy	73/1468	11/1468		4/1468	1/1468		6/1468		[398]
USA	1001/14802	256/14802	36/14802	183/14802	3/14802	33/14802	83/14802	16/14802	[399]
China	1/358		57/358						[400]
India						14/26			[401]
Morocco	24/50								[402]
Croatia	28/54					1/54			[403]
Croatia	41/116			2/116		1/116			[404]
Iran	2/448					4/448			[405]
Czech Republic	113/312	2/312				13/312			[406]
Pakistan	4/300		2/300			9/300			[407]
Israel	2/2								[408]
Turkey	13/70								[409]
16 countries	2103/3062	70/3062	1/3062	56/3062		37/3062	79/3062	10/3062	[410]
Israel	31/150						21/150		[411]

Macedonia	15/66			2/66		4/66			[412]
Latvia	69/130	3/130	1/130						[413]
USA	48/204						9/204		[414]
Belgium	21/32								[415]
Japan			19/106					1/106	[416]
Iran	6/12								[417]
Algeria	34/160								[418]
Algeria	8/22								[419]
USA	1/20							1/20	[420]
Hungary	104/NA								[421]
Romania	17/174								[422]
Tunisia	45/190			1/190					[423]
Venezuela	13/80								[424]
Germany	2/46								[425]
China	24/194		4/194						[426]
Poland	73/204	1/204							[427]
USA			16/428	93/428					[428]
China	2/764		111/764		20/764			8/764	[429]
China			80/465	86/465	6/465				[430]
China			10/180	20/180					[431]
China			2/2						[432]
China			148/1012	33/1012	13/1012			2/1012	[433]
China	2/138		20/138						[434]
China	13/236		25/236						[435]
China			3/42						[436]
China	35/202						4/202		[437]
Australia			31/520		7/520				[438]
Turkey	51/302								[439]
China			222/2380		62/2380				[440]
China			140/678	17/678	38/678			2/678	[441]
China			6/472	12/472	1/472			1/472	[442]
China	1/636		50/636		13/636				[443]
Germany	54/456	9/456							[444]
France	69/280								[445]
USA	304/1474	11/1474		5/1474	4/1474				[225]
USA	33/768			2/768				1/768	[361]

The numerators in this column represent the number of mutated alleles, and the denominators the total number of screened alleles.)

NA, not applicable (the authors were not clear on the total number of alleles they have screened

Table S4. Known but rare PLP *GJB2* variants common in isolated populations.

	Variant	(c.IVS1+1G>A)		p.W172* (c.516G>A)		p.W172C (c.516G>C)		p.W172R (c.514T>A)		p.W44* (c.131G>A)	
	Variant reference number			rs1302739538		rs1302739538		rs770330002		rs104894407	
Country	Continent	n/N	%	n/N	%	n/N	%	n/N	%	n/N	%
Algeria	Africa	2/118	1.7%								
Ghana	Africa									2/162	1.2%
Egypt	Africa	1/222	0.5%								
Bangladesh	Asia	10/106	9.4%								
China	Asia	12/2502	0.5%								
India	Asia	8/1062	0.8%					4/1060	0.4%		
Iran	Asia	134/7842	1.7%								
Mongolia	Asia	42/1068	3.9%	1/1068	0.1%						
Palestin	Asia	1/96	1.0%								
Qatar	Asia	10/252	4.0%								
Russia	Asia	13/140	9.3%			52/592	8.8%				
Syria	Asia	14/140	10.0%								
Turkey	Asia	55/4814	1.1%	1/3062	0.03%						
Yakutia	Asia	382/702	54.4%								
Austria	Europe	3/398	0.8%								
Belgium	Europe	2/116	1.7%								
Czech	Europe	9/312	2.9%								
France	Europe	7/830	0.8%							1/318	0.3%
Italy	Europe	18/1338	1.3%								
Netherlands	Europe	3/488	0.6%								
Poland	Europe	24/466	5.2%								
Portugal	Europe	2/529	0.4%	3/528	0.6%						
Slovakia	Europe	3/546	0.5%								
USA	North America	14/4736	0.3%	2/15090	0.0%					6/2918	0.2%
Argentina	South America	1/188	0.5%								
Brazil	South America	5/1570	0.3%	3/632	0.5%						
Guatemala	North America									21/266	7.9%

n/N = number of affected alleles out of total alleles tested, % = allele frequency.

References

1. 1. Khalifa Alkowari, M.; Girotto, G.; Abdulhadi, K.; Dipresa, S.; Siam, R.; Najjar, N.; Badii, R.; Gasparini, P. GJB2 and GJB6 genes and the A1555G mitochondrial mutation are only minor causes of nonsyndromic hearing loss in the Qatari population. *International journal of audiology* **2012**, *51*, 181-185, doi:10.3109/14992027.2011.625983.
2. 2. Tang, H.Y.; Fang, P.; Ward, P.A.; Schmitt, E.; Darilek, S.; Manolidis, S.; Oghalai, J.S.; Roa, B.B.; Alford, R.L. DNA sequence analysis of GJB2, encoding connexin 26: Observations from a population of hearing impaired cases and variable carrier rates, complex genotypes, and ethnic stratification of alleles among controls. *American Journal of Medical Genetics Part A* **2006**, *140a*, 2401-2415, doi:10.1002/ajmg.a.31525.
3. 3. Posukh, O.; Pallares-Ruiz, N.; Tadinova, V.; Osipova, L.; Claustres, M.; Roux, A.F. First molecular screening of deafness in the Altai Republic population. *BMC Med Genet* **2005**, *6*, 12, doi:10.1186/1471-2350-6-12.
4. 4. Azadegan-Dehkordi, F.; Bahrami, T.; Shirzad, M.; Karbasi, G.; Yazdanpanahi, N.; Farrokhi, E.; Koohiyan, M.; Tabatabaiefar, M.A.; Hashemzadeh-Chaleshtori, M. Mutations in GJB2 as Major Causes of Autosomal Recessive Non-Syndromic Hearing Loss: First Report of c.299-300delAT Mutation in Kurdish Population of Iran. *Journal of Audiology and Otology* **2019**, *23*, 20-26, doi:10.7874/jao.2018.00185.
5. 5. Banjara, H.; Mungutwar, V.; Swarnkar, N.; Patra, P. Detection of Connexion 26 GENE (GJB2) Mutations in Cases of Congenital Non Syndromic Deafness. *Indian journal of otolaryngology and head and neck surgery : official publication of the Association of Otolaryngologists of India* **2016**, *68*, 248-253, doi:10.1007/s12070-015-0950-4.
6. 6. Sobe, T.; Vreugde, S.; Shahin, H.; Berlin, M.; Davis, N.; Kanaan, M.; Yaron, Y.; Orr-Urtreger, A.; Frydman, M.; Shohat, M. The prevalence and expression of inherited connexin 26 mutations associated with nonsyndromic hearing loss in the Israeli population. *Human genetics* **2000**, *106*, 50-57.
7. 7. Batissoco, A.C.; Abreu-Silva, R.S.; Braga, M.C.C.; Lezirovitz, K.; Della-Rosa, V.; Alfredo Jr, T.; Otto, P.A.; Mingroni-Netto, R.C. Prevalence of GJB2 (connexin-26) and GJB6 (connexin-30) mutations in a cohort of 300 Brazilian hearing-impaired individuals: implications for diagnosis and genetic counseling. *Ear and hearing* **2009**, *30*, 1-7.
8. 8. Minarik, G.; Tretinarova, D.; Szemes, T.; Kadasi, L. Prevalence of DFNB1 mutations in Slovak patients with non-syndromic hearing loss. *International journal of pediatric otorhinolaryngology* **2012**, *76*, 400-403, doi:10.1016/j.ijporl.2011.12.020.
9. 9. Shan, J.; Chobot-Rodd, J.; Castellanos, R.; Babcock, M.; Shanske, A.; Parikh, S.R.; Morrow, B.E.; Samanich, J. GJB2 mutation spectrum in 209 hearing impaired individuals of predominantly Caribbean Hispanic and African descent. *International journal of pediatric otorhinolaryngology* **2010**, *74*, 611-618, doi:10.1016/j.ijporl.2010.03.004.
10. 10. Chen, K.; Wu, X.; Zong, L.; Jiang, H. GJB3/GJB6 screening in GJB2 carriers with idiopathic hearing loss: Is it necessary? *J Clin Lab Anal* **2018**, *32*, e22592, doi:10.1002/jcla.22592.

11. 11. Frei, K.; Ramsebner, R.; Lucas, T.; Hamader, G.; Szuhai, K.; Weipoltshammer, K.; Baumgartner, W.D.; Wachtler, F.J.; Kirschhofer, K. GJB2 mutations in hearing impairment: identification of a broad clinical spectrum for improved genetic counseling. *The Laryngoscope* **2005**, *115*, 461-465, doi:10.1097/01.mlg.0000157855.47143.71.
12. 12. Frei, K.; Szuhai, K.; Lucas, T.; Weipoltshammer, K.; Schofer, C.; Ramsebner, R.; Baumgartner, W.D.; Raap, A.K.; Bittner, R.; Wachtler, F.J., et al. Connexin 26 mutations in cases of sensorineural deafness in eastern Austria. *European journal of human genetics : EJHG* **2002**, *10*, 427-432, doi:10.1038/sj.ejhg.5200826.
13. 13. Bonyadi, M.; Esmaeili, M.; Abhari, M.; Lotfi, A. Mutation analysis of familial GJB2-related deafness in Iranian Azeri Turkish patients. *Genet Test Mol Biomarkers* **2009**, *13*, 689-692, doi:10.1089/gtmb.2009.0026.
14. 14. Bozhkova, V.P.; Khashaev, Z.H.; Umanskaya, T.M. Frequence and the mutation spectrum of GJB2-related hearing loss in children of Dagestan as compared with the central European part of Russia. *Biophysics* **2010**, *55*, 453-462, doi:10.1134/s0006350910030176.
15. 15. Naseri, M.; Akbarzadehlaleh, M.; Masoudi, M.; Ahangari, N.; Poursadegh Zonouzi, A.A.; Poursadegh Zonouzi, A.; Shams, L.; Nejatizadeh, A. Genetic Linkage Analysis of DFNB4, DFNB28, DFNB93 Loci in Autosomal Recessive Non-syndromic Hearing Loss: Evidence for Digenic Inheritance in GJB2 and GJB3 Mutations. *Iran J Public Health* **2018**, *47*, 95-102.
16. 16. Roux, A.F.; Pallares-Ruiz, N.; Vielle, A.; Faugere, V.; Templin, C.; Leprevost, D.; Artieres, F.; Lina, G.; Molinari, N.; Blanchet, P., et al. Molecular epidemiology of DFNB1 deafness in France. *BMC Med Genet* **2004**, *5*, 5, doi:10.1186/1471-2350-5-5.
17. 17. Wattanasirichaigoon, D.; Limwongse, C.; Jariengprasert, C.; Yenchitsomanus, P.T.; Tocharoenthanaphol, C.; Thongnoppakhun, W.; Thawil, C.; Charoenpipop, D.; Pho-iam, T.; Thongpradit, S., et al. High prevalence of V37I genetic variant in the connexin-26 (GJB2) gene among non-syndromic hearing-impaired and control Thai individuals. *Clinical genetics* **2004**, *66*, 452-460, doi:10.1111/j.1399-0004.2004.00325.x.
18. 18. Abe, S.; Nishio, S.Y.; Yokota, Y.; Moteki, H.; Kumakawa, K.; Usami, S.I. Diagnostic pitfalls for GJB2-related hearing loss: A novel deletion detected by Array-CGH analysis in a Japanese patient with congenital profound hearing loss. *Clin Case Rep* **2018**, *6*, 2111-2116, doi:10.1002/ccr3.1800.
19. 19. Abe, S.; Usami, S.; Shinkawa, H.; Kelley, P.M.; Kimberling, W.J. Prevalent connexin 26 gene (GJB2) mutations in Japanese. *J Med Genet* **2000**, *37*, 41-43, doi:10.1136/jmg.37.1.41.
20. 20. Abidi, O.; Boulouiz, R.; Nahili, H.; Ridal, M.; Alami, M.N.; Tlili, A.; Rouba, H.; Masmoudi, S.; Chafik, A.; Hassar, M. GJB2 (connexin 26) gene mutations in Moroccan patients with autosomal recessive non-syndromic hearing loss and carrier frequency of the common GJB2–35delG mutation. *International journal of pediatric otorhinolaryngology* **2007**, *71*, 1239-1245.
21. 21. Adadey, S.M.; Manyisa, N.; Mnika, K.; de Kock, C.; Nembaware, V.; Quaye, O.; Amedofu, G.K.; Awandare, G.A.; Wonkam, A. GJB2 and GJB6 Mutations in Non-Syndromic Childhood Hearing Impairment in Ghana. *Front Genet* **2019**, *10*, 1-10, doi:10.3389/fgene.2019.00841.
22. 22. Adato, A.; Raskin, L.; Petit, C.; Bonne-Tamir, B. Deafness heterogeneity in a Druze isolate from the Middle East: novel OTOF and PDS mutations, low prevalence of GJB2 35delG mutation and indication for a new DFNB locus. *European journal of human genetics : EJHG* **2000**, *8*, 437-442, doi:10.1038/sj.ejhg.5200489.

23. 23. Aishah, Z.S.; Khairi, M.D.; Normastura, A.R.; Zafarina, Z.; Zilfalil, B.A. Screening for gap junction protein beta-2 gene mutations in Malays with autosomal recessive, non-syndromic hearing loss, using denaturing high performance liquid chromatography. *The Journal of laryngology and otology* **2008**, *122*, 1284-1288, doi:10.1017/s0022215108002041.
24. 24. Al-Achkar, W.; Al-Halabi, B.; Ali, B.; Moassass, F. First report of prevalence c.IVS1+1G>A and del (GJB6-13S1854) mutations in Syrian families with non-syndromic sensorineural hearing loss. *International journal of pediatric otorhinolaryngology* **2017**, *92*, 82-87, doi:10.1016/j.ijporl.2016.11.015.
25. 25. Al-Achkar, W.; Moassass, F.; Al-Halabi, B.; Al-Ablog, A. Mutations of the Connexin 26 gene in families with non-syndromic hearing loss. *Molecular medicine reports* **2011**, *4*, 331-335, doi:10.3892/mmr.2011.428.
26. 26. Al-Qahtani, M.H.; Baghlab, I.; Chaudhary, A.G.; Abuzenadah, A.M.; Bamanie, A.; Daghistani, K.J.; Safieh, M.; Fida, L.; Dallol, A. Spectrum of GJB2 mutations in a cohort of nonsyndromic hearing loss cases from the Kingdom of Saudi Arabia. *Genet Test Mol Biomarkers* **2010**, *14*, 79-83, doi:10.1089/gtmb.2009.0111.
27. 27. Al-Sebeih, K.; Al-Kandari, M.; Al-Awadi, S.A.; Hegazy, F.F.; Al-Khamees, G.A.; Naguib, K.K.; Al-Dabbous, R.M. Connexin 26 gene mutations in non-syndromic hearing loss among Kuwaiti patients. *Medical principles and practice : international journal of the Kuwait University, Health Science Centre* **2014**, *23*, 74-79, doi:10.1159/000348304.
28. 28. Alvarez, A.; del Castillo, I.; Pera, A.; Villamar, M.; Moreno-Pelayo, M.A.; Rivera, T.; Solanellas, J.; Moreno, F. Uniparental disomy of chromosome 13q causing homozygosity for the 35delG mutation in the gene encoding connexin26 (GJB2) results in prelingual hearing impairment in two unrelated Spanish patients. *J Med Genet* **2003**, *40*, 636-639, doi:10.1136/jmg.40.8.636.
29. 29. Alvarez, A.; del Castillo, I.; Villamar, M.; Aguirre, L.A.; Gonzalez-Neira, A.; Lopez-Nevot, A.; Moreno-Pelayo, M.A.; Moreno, F. High prevalence of the W24X mutation in the gene encoding connexin-26 (GJB2) in Spanish Romani (gypsies) with autosomal recessive non-syndromic hearing loss. *American journal of medical genetics. Part A* **2005**, *137a*, 255-258, doi:10.1002/ajmg.a.30884.
30. 30. Ammar-Khodja, F.; Bonnet, C.; Dahmani, M.; Ouhab, S.; Lefevre, G.M.; Ibrahim, H.; Hardelin, J.P.; Weil, D.; Louha, M.; Petit, C. Diversity of the causal genes in hearing impaired Algerian individuals identified by whole exome sequencing. *Mol Genet Genomic Med* **2015**, *3*, 189-196, doi:10.1002/mgg3.131.
31. 31. Ammar-Khodja, F.; Faugere, V.; Baux, D.; Giannesini, C.; Leonard, S.; Makrelouf, M.; Malek, R.; Djennaoui, D.; Zenati, A.; Claustres, M., et al. Molecular screening of deafness in Algeria: high genetic heterogeneity involving DFNB1 and the Usher loci, DFNB2/USH1B, DFNB12/USH1D and DFNB23/USH1F. *Eur J Med Genet* **2009**, *52*, 174-179, doi:10.1016/j.ejmg.2009.03.018.
32. 32. Ammar-Khodja, F.; Makrelouf, M.; Malek, R.; Ibrahim, H.; Zenati, A. Frequency of the 35delG allele causing nonsyndromic recessive deafness in the Algerian patients. *Genetic counseling (Geneva, Switzerland)* **2007**, *18*, 383-391.
33. 33. Angeli, S.; Utrera, R.; Dib, S.; Chiossone, E.; Naranjo, C.; Henriquez, O.; Porta, M. GJB2 gene mutations in childhood deafness. *Acta oto-laryngologica* **2000**, *120*, 133-136, doi:10.1080/000164800750000766.

34. 34. Antoniadi, T.; Pampanos, A.; Petersen, M.B. Prenatal diagnosis of prelingual deafness: carrier testing and prenatal diagnosis of the common GJB2 35delG mutation. *Prenat Diagn* **2001**, *21*, 10-13, doi:10.1002/1097-0223(200101)21:1<10::aid-pd968>3.0.co;2-h.

35. 35. Atik, T.; Onay, H.; Aykut, A.; Bademci, G.; Kirazli, T.; Tekin, M.; Ozkinay, F. Comprehensive Analysis of Deafness Genes in Families with Autosomal Recessive Nonsyndromic Hearing Loss. *PloS one* **2015**, *10*, e0142154, doi:10.1371/journal.pone.0142154.

36. 36. Bajaj, Y.; Sirimanna, T.; Albert, D.M.; Qadir, P.; Jenkins, L.; Bitner-Glindzicz, M. Spectrum of GJB2 mutations causing deafness in the British Bangladeshi population. *Clinical otolaryngology : official journal of ENT-UK ; official journal of Netherlands Society for Oto-Rhino-Laryngology & Cervico-Facial Surgery* **2008**, *33*, 313-318, doi:10.1111/j.1749-4486.2008.01754.x.

37. 37. Bakhchane, A.; Bousfiha, A.; Charoute, H.; Salime, S.; Detsouli, M.; Snoussi, K.; Nadifi, S.; Kabine, M.; Rouba, H.; Dehbi, H., et al. Update of the spectrum of GJB2 gene mutations in 152 Moroccan families with autosomal recessive nonsyndromic hearing loss. *Eur J Med Genet* **2016**, *59*, 325-329, doi:10.1016/j.ejmg.2016.05.002.

38. 38. Balta, B.; Gundogdu, R.; Erdogan, M.; Alisik, M.; Kiraz, A.; Ozcan, I.; Erel, O. Decreased disulphide/thiol ratio in patients with autosomal recessive non-syndromic hearing loss. *International journal of pediatric otorhinolaryngology* **2018**, *112*, 188-192, doi:10.1016/j.ijporl.2018.07.014.

39. 39. Barashkov, N.A.; Pshennikova, V.G.; Posukh, O.L.; Teryutin, F.M.; Solovyev, A.V.; Klarov, L.A.; Romanov, G.P.; Gotovtsev, N.N.; Kozhevnikov, A.A.; Kirillina, E.V., et al. Spectrum and Frequency of the GJB2 Gene Pathogenic Variants in a Large Cohort of Patients with Hearing Impairment Living in a Subarctic Region of Russia (the Sakha Republic). *PloS one* **2016**, *11*, e0156300, doi:10.1371/journal.pone.0156300.

40. 40. Barış, I.; Kılınç, M.; Tolun, A. Frequency of the 35delG mutation in the connexin 26 gene in Turkish hearing ‐ impaired patients. *Clinical genetics* **2001**, *60*, 452-455.

41. 41. Bason, L.; Dudley, T.; Lewis, K.; Shah, U.; Potsic, W.; Ferraris, A.; Fortina, P.; Rappaport, E.; Krantz, I.D. Homozygosity for the V37I Connexin 26 mutation in three unrelated children with sensorineural hearing loss. *Clinical genetics* **2002**, *61*, 459-464, doi:10.1034/j.1399-0004.2002.610611.x.

42. 42. Batissoco, A.C.; Auricchio, M.T.; Kimura, L.; Tabith-Junior, A.; Mingroni-Netto, R.C. A novel missense mutation p.L76P in the GJB2 gene causing nonsyndromic recessive deafness in a Brazilian family. *Brazilian journal of medical and biological research = Revista brasileira de pesquisas medicas e biologicas* **2009**, *42*, 168-171, doi:10.1590/s0100-879x2009000200004.

43. 43. Battelino, S.; Repic Lampret, B.; Zargi, M.; Podkrajsek, K.T. Novel connexin 30 and connexin 26 mutational spectrum in patients with progressive sensorineural hearing loss. *The Journal of laryngology and otology* **2012**, *126*, 763-769, doi:10.1017/S0022215112001119.

44. 44. Bayazit, Y.A.; Cable, B.B.; Cataloluk, O.; Kara, C.; Chamberlin, P.; Smith, R.J.; Kanlikama, M.; Ozer, E.; Cakmak, E.A.; Mumbuc, S., et al. GJB2 gene mutations causing familial hereditary deafness in Turkey. *International journal of pediatric otorhinolaryngology* **2003**, *67*, 1331-1335, doi:10.1016/j.ijporl.2003.08.003.

45. 45. Bazazzadegan, N.; Nikzat, N.; Fattahi, Z.; Nishimura, C.; Meyer, N.; Sahraian, S.; Jamali, P.; Babanejad, M.; Kashef, A.; Yazdan, H., et al. The spectrum of GJB2 mutations in the Iranian population with non-syndromic hearing loss--a twelve year study. *International journal of pediatric otorhinolaryngology* **2012**, *76*, 1164-1174, doi:10.1016/j.ijporl.2012.04.026.

46. 46. Beck, C.; Perez-Alvarez, J.C.; Sigruener, A.; Haubner, F.; Seidler, T.; Aslanidis, C.; Strutz, J.; Schmitz, G. Identification and genotype/phenotype correlation of mutations in a large German cohort with hearing loss. *European archives of oto-rhino-laryngology : official journal of the European Federation of Oto-Rhino-Laryngological Societies* **2015**, *272*, 2765-2776, doi:10.1007/s00405-014-3157-5.
47. 47. Belguith, H.; Tlili, A.; Dhouib, H.; Ben Rebeh, I.; Lahmar, I.; Charfeddine, I.; Driss, N.; Ghorbel, A.; Ayadi, H.; Masmoudi, S. Mutation in gap and tight junctions in patients with non-syndromic hearing loss. *Biochemical and biophysical research communications* **2009**, *385*, 1-5, doi:10.1016/j.bbrc.2009.02.125.
48. 48. Piatto, V.B.; Bertollo, E.M.G.; Sartorato, E.L.; Maniglia, J.V. Prevalence of the GJB2 mutations and the del(GJB6-D13S1830) mutation in Brazilian patients with deafness. *Hearing research* **2004**, *196*, 87-93, doi:10.1016/j.heares.2004.05.007.
49. 49. Belintani Piatto, V.; Vasques Moreira, O.A.; Orate Menezes da Silva, M.A.; Victor Maniglia, J.; Coimbra Pereira, M.; Sartorato, E.L. Correlation between audiometric data and the 35delG mutation in ten patients. *Brazilian journal of otorhinolaryngology* **2007**, *73*, 777-783, doi:10.1016/s1808-8694(15)31174-5.
50. 50. Ben Arab, S.; Hmani, M.; Denoyelle, F.; Boulila-Elgaied, A.; Chardenoux, S.; Hachicha, S.; Petit, C.; Ayadi, H. Mutations of GJB2 in three geographic isolates from northern Tunisia: evidence for genetic heterogeneity within isolates. *Clinical genetics* **2000**, *57*, 439-443, doi:10.1034/j.1399-0004.2000.570607.x.
51. 51. Bhalla, S.; Sharma, R.; Khandelwal, G.; Panda, N.K.; Khullar, M. Low incidence of GJB2, GJB6 and mitochondrial DNA mutations in North Indian patients with non-syndromic hearing impairment. *Biochemical and biophysical research communications* **2009**, *385*, 445-448, doi:10.1016/j.bbrc.2009.05.083.
52. 52. Bliznetz, E.A.; Galkina, V.A.; Matyushchenko, G.N.; Kisina, A.G.; Markova, T.G.; Polyakov, A.V. Changes in the connexin 26 gene (GJB2) in Russian patients with hearing loss: Results of long-term molecular diagnostics of hereditary nonsyndromic hearing loss. *Russian Journal of Genetics* **2012**, *48*, 101-112, doi:10.1134/S1022795412010036.
53. 53. Bliznetz, E.A.; Martsul, D.N.; Khorov, O.G.; Markova, T.G.; Polyakov, A.V. Spectrum of the GJB2 Mutations in Belarussian Patients with Hearing Loss. Findings of Pilot Genetic Screening of Hearing Impairment in Newborns. *Russian Journal of Genetics* **2014**, *50*, 191-197, doi:10.1134/S1022795414020033.
54. 54. Bolz, H.; Schade, G.; Ehmer, S.; Kothe, C.; Hess, M.; Gal, A. Phenotypic variability of non-syndromic hearing loss in patients heterozygous for both c.35delG of GJB2 and the 342-kb deletion involving GJB6. *Hearing research* **2004**, *188*, 42-46, doi:10.1016/s0378-5955(03)00346-0.
55. 55. Bonyadi, M.; Fotouhi, N.; Esmaeili, M. Prevalence of IVS1+1G>A mutation among Iranian Azeri Turkish patients with autosomal recessive non-syndromic hearing loss (ARNSHL). *International journal of pediatric otorhinolaryngology* **2011**, *75*, 1612-1615, doi:10.1016/j.ijporl.2011.09.024.
56. 56. Bonyadi, M.J.; Fotouhi, N.; Esmaeili, M. Spectrum and frequency of GJB2 mutations causing deafness in the northwest of Iran. *International journal of pediatric otorhinolaryngology* **2014**, *78*, 637-640, doi:10.1016/j.ijporl.2014.01.022.
57. 57. Bors, A.; Andrikovics, H.; Kalmar, L.; Erdei, N.; Galambos, S.; Losonczi, A.; Furedi, S.; Balogh, I.; Szalai, C.; Tordai, A. Frequencies of two common mutations (c.35delG and c.167delT) of the connexin 26 gene in different populations of Hungary. *Int J Mol Med* **2004**, *14*, 1105-1108.
58. 58. Bozdogan, S.T.; Kuran, G.; Yuregir, O.O.; Aslan, H.; Haytoglu, S.; Ayaz, A.; Arikan, O.K. The Prevalence of Gap Junction Protein Beta 2 (GJB2) Mutations in Non Syndromic Sensorineural Hearing Loss in Cukurova Region. *J Int Adv Otol* **2015**, *11*, 118-121, doi:10.5152/iao.2015.1212.

59. 59. Brobby, G.W.; Muller-Myhsok, B.; Horstmann, R.D. Connexin 26 R143W mutation associated with recessive nonsyndromic sensorineural deafness in Africa. *The New England journal of medicine* **1998**, *338*, 548-550, doi:10.1056/NEJM199802193380813.

60. 60. Bukhari, I.; Mujtaba, G.; Naz, S. Contribution of GJB2 mutations to hearing loss in the Hazara Division of Pakistan. *Biochem Genet* **2013**, *51*, 524-529, doi:10.1007/s10528-013-9583-z.

61. 61. Burke, W.F.; Warnecke, A.; Schoner-Heinisch, A.; Lesinski-Schiedat, A.; Maier, H.; Lenarz, T. Prevalence and audiological profiles of GJB2 mutations in a large collective of hearing impaired patients. *Hearing research* **2016**, *333*, 77-86, doi:10.1016/j.heares.2016.01.006.

62. 62. Cama, E.; Melchionda, S.; Palladino, T.; Carella, M.; Santarelli, R.; Genovese, E.; Benettazzo, F.; Zelante, L.; Arslan, E. Hearing loss features in GJB2 biallelic mutations and GJB2/GJB6 digenic inheritance in a large Italian cohort. *International journal of audiology* **2009**, *48*, 12-17, doi:10.1080/14992020802400654.

63. 63. Carlsson, P.I.; Karltorp, E.; Carlsson-Hansen, E.; Ahlman, H.; Moller, C.; Vondobeln, U. GJB2 (Connexin 26) gene mutations among hearing-impaired persons in a Swedish cohort. *Acta oto-laryngologica* **2012**, *132*, 1301-1305, doi:10.3109/00016489.2012.701018.

64. 64. Caroça, C.; de Matos, T.M.; Ribeiro, D.; Lourenço, V.; Martins, T.; Campelo, P.; Fialho, G.; Silva, S.N.; Paço, J.; Caria, H. Genetic Basis of Nonsyndromic Sensorineural Hearing Loss in the Sub-Saharan African Island Population of São Tomé and Príncipe: The Role of the DFNB1 Locus? *Omics: a journal of integrative biology* **2016**, *20*, 449-455.

65. 65. Carranza, C.; Menendez, I.; Herrera, M.; Castellanos, P.; Amado, C.; Maldonado, F.; Rosales, L.; Escobar, N.; Guerra, M.; Alvarez, D., et al. A Mayan founder mutation is a common cause of deafness in Guatemala. *Clinical genetics* **2016**, *89*, 461-465, doi:10.1111/cge.12676.

66. 66. Castro, L.S.; Marinho, A.N.; Rodrigues, E.M.; Marques, G.C.; Carvalho, T.A.; Silva, L.C.; dos Santos, S.E. C. *Brazilian journal of otorhinolaryngology* **2013**, *79*, 95-99, doi:10.5935/1808-8694.20130016.

67. 67. Chai, Y.; Pang, X.; Chen, D.; Li, L.; Chen, Y.; Sun, L.; Wang, X.; Wu, H.; Yang, T. Molecular etiology of non-dominant, non-syndromic, mild-to-moderate childhood hearing impairment in Chinese Hans. *American journal of medical genetics. Part A* **2014**, *164a*, 3115-3119, doi:10.1002/ajmg.a.36785.

68. 68. Chai, Y.C.; Sun, L.H.; Pang, X.H.; Wang, X.W.; Chen, D.Y.; Chen, Y.; Wu, H.; Yang, T. Identification of both MT-RNR1 m.1555A > G and bi-allelic GJB2 mutations in probands with non-syndromic hearing loss. *International journal of pediatric otorhinolaryngology* **2014**, *78*, 614-617, doi:10.1016/j.ijporl.2014.01.011.

69. 69. Chaleshtori, M.H.; Farhud, D.; Taylor, R.; Hadavi, V.; Patton, M.; Afzal, A. Deafness–associated connexin 26 gene (GJB2) mutations in Iranian population. *Iranian Journal of Public Health* **2002**, 75-79.

70. 70. Chan, D.K.; Schrijver, I.; Chang, K.W. Connexin-26–associated deafness: Phenotypic variability and progression of hearing loss. *Genetics in Medicine* **2010**, *12*, 174.

71. 71. Chan, D.K.; Schrijver, I.; Chang, K.W. Diagnostic Yield in the Workup of Congenital Sensorineural Hearing Loss Is Dependent on Patient Ethnicity. *Otology & Neurotology* **2011**, *32*, 81-87, doi:10.1097/MAO.0b013e3181fc786f.

72. 72. Chen, D.; Chen, X.; Cao, K.; Zuo, J.; Jin, X.; Wei, C.; Fang, F. High prevalence of the connexin 26 (GJB2) mutation in Chinese cochlear implant recipients. *ORL; journal for oto-rhino-laryngology and its related specialties* **2009**, *71*, 212-215, doi:10.1159/000229300.

73. 73. Chen, D.Y.; Chen, X.W.; Cao, K.L.; Jin, X.; Zuo, J.; Wei, C.G.; Fang, F.D. [High prevalence of connexin-26 (GJB2) mutation in cochlear implant recipients]. *Zhonghua yi xue za zhi* **2006**, *86*, 3114-3117.

74. 74. Chen, G.; Fu, S.; Dong, J.; Chen, P. Low frequency of GJB2 mutations in thirty-five students with hearing loss in Chinese consanguineous families. *International journal of pediatric otorhinolaryngology* **2011**, *75*, 1535-1537, doi:10.1016/j.ijporl.2011.09.001.

75. 75. Chen, G.; Liu, J.; Dong, J.; Li, J.; Fu, S. GJB2 mutations are rare in probands with hearing loss in Chinese assortative mating families. *International journal of pediatric otorhinolaryngology* **2014**, *78*, 244-247, doi:10.1016/j.ijporl.2013.11.013.

76. 76. Chen, K.; Zong, L.; Liu, M.; Wang, X.; Zhou, W.; Zhan, Y.; Cao, H.; Dong, C.; Tang, H.; Jiang, H. Developing regional genetic counseling for southern Chinese with nonsyndromic hearing impairment: a unique mutational spectrum. *J Transl Med* **2014**, *12*, 64, doi:10.1186/1479-5876-12-64.

77. 77. Chen, Y.; Cao, Y.; Li, H.B.; Mao, J.; Liu, M.J.; Liu, Y.H.; Wang, B.J.; Jiang, D.; Zhu, Q.; Ding, Y., et al. SNaPshot reveals high mutation and carrier frequencies of 15 common hearing loss mutants in a Chinese newborn cohort. *Clinical genetics* **2015**, *87*, 467-472, doi:10.1111/cge.12452.

78. 78. Chen, Y.; Liu, Y.; Wang, B.; Mao, J.; Wang, T.; Ye, K.; Ye, Y.; Cram, D.S.; Li, H. Development and validation of a fetal genotyping assay with potential for noninvasive prenatal diagnosis of hereditary hearing loss. *Prenat Diagn* **2016**, *36*, 1233-1241, doi:10.1002/pd.4962.

79. 79. Chen, Y.; Tudi, M.; Sun, J.; He, C.; Lu, H.L.; Shang, Q.; Jiang, D.; Kuyaxi, P.; Hu, B.; Zhang, H. Genetic mutations in non-syndromic deafness patients of Uyghur and Han Chinese ethnicities in Xinjiang, China: a comparative study. *J Transl Med* **2011**, *9*, 154, doi:10.1186/1479-5876-9-154.

80. 80. Chinetti, V.; Iossa, S.; Auletta, G.; Corvino, V.; De Luca, M.; De Falco, F.; Giannini, P.; Lilli, G.; Malesci, R.; Riccardi, P., et al. Mutational analysis for GJB2, GJB6, and GJB3 genes in Campania within a universal neonatal hearing screening programme. *International journal of audiology* **2011**, *50*, 866-870, doi:10.3109/14992027.2011.603757.

81. 81. Choi, S.Y.; Lee, K.Y.; Kim, Y.E.; Bae, J.W.; Oh, S.K.; Kim, S.Y.; Hwang, S.J.; Kim, U.K.; Lee, S.H. Application of allele-specific primer extension-based microarray for simultaneous multi-gene mutation screening in patients with non-syndromic hearing loss. *Int J Mol Med* **2010**, *25*, 315-320, doi:10.3892/ijmm_00000347.

82. 82. Christiani, T.V.; Alexandrino, F.; de Oliveira, C.A.; Amantini, R.C.; Bevilacqua, M.C.; Filho, O.A.; Porto, P.; Sartorato, E.L. Molecular study in Brazilian cochlear implant recipients. *American journal of medical genetics. Part A* **2007**, *143a*, 1580-1582, doi:10.1002/ajmg.a.31778.

83. 83. Cifuentes, L.; Arancibia, M.; Torrente, M.; Acuna, M.; Farfan, C.; Rios, C. Prevalence of the 35delG mutation in the GJB2 gene in two samples of non-syndromic deaf subjects from Chile. *Biological research* **2013**, *46*, 239-242, doi:10.4067/s0716-97602013000300003.

84. 84. Coco, M.; Salvinelli, F.; Greco, F.; Trivelli, M.; D'Emidio, L.; Mesoraca, A.; Giorlandino, C.; Raffio, R.; Coco, C. Significance of heterozygosis M34T mutation of GJB2 gene in non-syndromic congenital deafness. Retrospective analysis of 12,472 samples of amniotic fluid. *J Prenat Med* **2013**, *7*, 56-58.

85. 85. Cohn, E.S.; Kelley, P.M.; Fowler, T.W.; Gorga, M.P.; Lefkowitz, D.M.; Kuehn, H.J.; Schaefer, G.B.; Gobar, L.S.; Hahn, F.J.; Harris, D.J., et al. Clinical studies of families with hearing loss attributable to mutations in the connexin 26 gene (GJB2/DFNB1). *Pediatrics* **1999**, *103*, 546-550, doi:10.1542/peds.103.3.546.
86. 86. Cordeiro-Silva Mde, F.; Barbosa, A.; Santiago, M.; Provetti, M.; Rabbi-Bortolini, E. Prevalence of 35delG/GJB2 and del (GJB6-D13S1830) mutations in patients with non-syndromic deafness from a population of Espirito Santo-Brazil. *Brazilian journal of otorhinolaryngology* **2010**, *76*, 428-432.
87. 87. Cryns, K.; Orzan, E.; Murgia, A.; Huygen, P.L.; Moreno, F.; del Castillo, I.; Chamberlin, G.P.; Azaiez, H.; Prasad, S.; Cucci, R.A., et al. A genotype-phenotype correlation for GJB2 (connexin 26) deafness. *J Med Genet* **2004**, *41*, 147-154, doi:10.1136/jmg.2003.013896.
88. 88. Dahl, H.H.; Saunders, K.; Kelly, T.M.; Osborn, A.H.; Wilcox, S.; Cone-Wesson, B.; Wunderlich, J.L.; Du Sart, D.; Kamarinos, M.; Gardner, R.J., et al. Prevalence and nature of connexin 26 mutations in children with non-syndromic deafness. *Med J Aust* **2001**, *175*, 191-194.
89. 89. Dai, P.; Yu, F.; Han, B.; Liu, X.; Wang, G.; Li, Q.; Yuan, Y.; Liu, X.; Huang, D.; Kang, D., et al. GJB2 mutation spectrum in 2,063 Chinese patients with nonsyndromic hearing impairment. *J Transl Med* **2009**, *7*, 26, doi:10.1186/1479-5876-7-26.
90. 90. Dai, P.; Yu, F.; Han, B.; Yuan, Y.; Li, Q.; Wang, G.; Liu, X.; He, J.; Huang, D.; Kang, D., et al. The prevalence of the 235delC GJB2 mutation in a Chinese deaf population. *Genet Med* **2007**, *9*, 283-289, doi:10.1097/gim.0b013e31804d2371.
91. 91. Dai, Z.Y.; Sun, B.C.; Huang, S.S.; Yuan, Y.Y.; Zhu, Y.H.; Su, Y.; Dai, P. Correlation analysis of phenotype and genotype of GJB2 in patients with non-syndromic hearing loss in China. *Gene* **2015**, *570*, 272-276, doi:10.1016/j.gene.2015.06.038.
92. 92. Dalamon, V.; Beheran, A.; Diamante, F.; Pallares, N.; Diamante, V.; Elgoyhen, A.B. Prevalence of GJB2 mutations and the del(GJB6-D13S1830) in Argentinean non-syndromic deaf patients. *Hearing research* **2005**, *207*, 43-49, doi:10.1016/j.heares.2005.04.012.
93. 93. Dalamon, V.; Florencia Wernert, M.; Lotersztein, V.; Craig, P.O.; Diamante, R.R.; Barteik, M.E.; Curet, C.; Paoli, B.; Mansilla, E.; Elgoyhen, A.B. Identification of four novel connexin 26 mutations in non-syndromic deaf patients: genotype-phenotype analysis in moderate cases. *Mol Biol Rep* **2013**, *40*, 6945-6955, doi:10.1007/s11033-013-2814-x.
94. 94. Dalamon, V.; Lotersztein, V.; Beheran, A.; Lipovsek, M.; Diamante, F.; Pallares, N.; Francipane, L.; Frechtel, G.; Paoli, B.; Mansilla, E., et al. GJB2 and GJB6 genes: molecular study and identification of novel GJB2 mutations in the hearing-impaired Argentinean population. *Audiology & neuro-otology* **2010**, *15*, 194-202, doi:10.1159/000254487.
95. 95. Danilenko, N.; Merkulava, E.; Siniauskaya, M.; Olejnik, O.; Levaya-Smaliak, A.; Kushniarevich, A.; Shymkevich, A.; Davydenko, O. Spectrum of genetic changes in patients with non-syndromic hearing impairment and extremely high carrier frequency of 35delG GJB2 mutation in Belarus. *PloS one* **2012**, *7*, e36354, doi:10.1371/journal.pone.0036354.
96. 96. Davoudi-Dehaghani, E.; Fallah, M.S.; Shirzad, T.; Tavakkoly-Bazzaz, J.; Bagherian, H.; Zeinali, S. Reporting the presence of three different diseases causing GJB2 mutations in a consanguineous deaf family. *International journal of audiology* **2014**, *53*, 128-131, doi:10.3109/14992027.2013.850748.

97. 97. de Barros Martinho, J.R.G.; Matos, T.D.M.; Martins, J.H.F.; Alves, M.C.; Andrade, S.M.S.; dos Santos Silva, L.F.; dos Reis Ribeiro, C.A.; de Sousa Antunes, M.C.; Fialho, M.G.M.A.; Caria, M.H.d.F.R. DFNB1-associated deafness in Portuguese cochlear implant users: prevalence and impact on oral outcome. *International journal of pediatric otorhinolaryngology* **2010**, *74*, 1135-1139.

98. 98. de Brouwer, A.P.; Pennings, R.J.; Roeters, M.; Van Hauwe, P.; Astuto, L.M.; Hoefsloot, L.H.; Huygen, P.L.; van den Helm, B.; Deutman, A.F.; Bork, J.M., et al. Mutations in the calcium-binding motifs of CDH23 and the 35delG mutation in GJB2 cause hearing loss in one family. *Human genetics* **2003**, *112*, 156-163, doi:10.1007/s00439-002-0833-0.

99. 99. de Freitas Cordeiro-Silva, M.; Barbosa, A.; Santiago, M.; Provetti, M.; Dettogni, R.S.; Tovar, T.T.; Rabbi-Bortolini, E.; Louro, I.D. Mutation analysis of GJB2 and GJB6 genes in Southeastern Brazilians with hereditary nonsyndromic deafness. *Molecular biology reports* **2011**, *38*, 1309-1313.

100. 100. de la Luz Arenas-Sordo, M.; Menendez, I.; Hernandez-Zamora, E.; Sirmaci, A.; Gutierrez-Tinajero, D.; McGetrick, M.; Murphy-Ruiz, P.; Leyva-Juarez, X.; Huesca-Hernandez, F.; Dominguez-Aburto, J., et al. Unique spectrum of GJB2 mutations in Mexico. *International journal of pediatric otorhinolaryngology* **2012**, *76*, 1678-1680, doi:10.1016/j.ijporl.2012.08.005.

101. 101. Dragomir, C.; Stan, A.; Stefanescu, D.T.; Sarafoleanu, C.; Severin, E.; Savu, L. GJB2 Gene Testing, Etiologic Diagnosis and Genetic Counseling in Romanian Persons With Prelingual Hearing Loss. *International Journal of Clinical Pediatrics* **2015**, *4*, 121-126.

102. 102. Dragomir, C.; Stan, A.; Stefanescu, D.T.; Savu, L.; Severin, E. Prenatal screening for the 35delG GJB2, del (GJB6-D13S1830), and del (GJB6-D13S1854) mutations in the Romanian population. *Genet Test Mol Biomarkers* **2011**, *15*, 749-753, doi:10.1089/gtmb.2011.0048.

103. 103. Duzcan, F.; Wollnik, B.; Tepeli, E.; Ardic, F.N.; Uyguner, O.; Bagci, H. [Family history, clinical features, and molecular characterization of a patient with autosomal recessive non-syndromic hearing loss]. *Kulak burun bogaz ihtisas dergisi : KBB = Journal of ear, nose, and throat* **2003**, *11*, 85-88.

104. 104. El Barbary, N.E.; El Belbesy, M.F.; Asal, S.I.; Kholeif, S.F. Detection of 35delG, 167delT mutations in the connexin 26 gene among Egyptian patients with nonsyndromic sensorineural hearing loss. *The Egyptian Journal of Otolaryngology* **2015**, *31*, 42.

105. 105. Elbagoury, N.M.; Soliman, H.N.; Mohammed, O.S.; Ghorab, E.A.; Elruby, M.O.; El-bassyouni, H.T.; Essawi, M.L. Mutation analysis of the GJB2 and GJB6 genes in Egyptian patients with autosomal recessive sensorineural nonsyndromic hearing loss. *Middle East Journal of Medical Genetics* **2014**, *3*, 11-15.

106. 106. Erbe, C.B.; Harris, K.C.; Runge-Samuelson, C.L.; Flanary, V.A.; Wackym, P.A. Connexin 26 and connexin 30 mutations in children with nonsyndromic hearing loss. *The Laryngoscope* **2004**, *114*, 607-611, doi:10.1097/00005537-200404000-00003.

107. 107. Esmaeili, M.; Bonyadi, M.; Nejadkazem, M. Common mutation analysis of GJB2 and GJB6 genes in affected families with autosomal recessive non-syndromic hearing loss from Iran: simultaneous detection of two common mutations (35delG/del(GJB6-D13S1830)) in the DFNB1-related deafness. *International journal of pediatric otorhinolaryngology* **2007**, *71*, 869-873, doi:10.1016/j.ijporl.2007.02.007.

108. 108. Esteves, M.C.; de Lima Isaac, M.; Francisco, A.M.; da Silva Junior, W.A.; Ferreira, C.A.; Dell'Aringa, A.H. Analysis of the presence of the GJB6 mutations in patients heterozygous for GJB2 mutation in Brazil. *European archives of oto-rhino-laryngology : official journal of the European Federation of Oto-Rhino-Laryngological Societies* **2014**, *271*, 695-699, doi:10.1007/s00405-013-2468-2.
109. 109. Estivill, X.; Fortina, P.; Surrey, S.; Rabionet, R.; Melchionda, S.; D'Agruma, L.; Mansfield, E.; Rappaport, E.; Govea, N.; Mila, M., et al. Connexin-26 mutations in sporadic and inherited sensorineural deafness. *Lancet* **1998**, *351*, 394-398, doi:10.1016/s0140-6736(97)11124-2.
110. 110. Farid, A.S.; Shabana, M.I.; Selim, M.H.; Al Sebeih, K.H.; Abdulla, M.M. Genetic study of autosomal recessive nonsyndromic sensorineural hearing loss in Kuwaiti children. *The Egyptian Journal of Otolaryngology* **2012**, *28*, 35.
111. 111. Feldmann, D.; Denoyelle, F.; Loundon, N.; Weil, D.; Garabedian, E.N.; Couderc, R.; Joannard, A.; Schmerber, S.; Delobel, B.; Leman, J., et al. Clinical evidence of the nonpathogenic nature of the M34T variant in the connexin 26 gene. *European journal of human genetics : EJHG* **2004**, *12*, 279-284, doi:10.1038/sj.ejhg.5201147.
112. 112. Figueroa-Ildefonso, E.; Bademci, G.; Rajabli, F.; Cornejo-Olivas, M.; Villanueva, R.D.C.; Badillo-Carrillo, R.; Inca-Martinez, M.; Neyra, K.M.; Sineni, C.; Tekin, M. Identification of Main Genetic Causes Responsible for Non-Syndromic Hearing Loss in a Peruvian Population. *Genes (Basel)* **2019**, *10*, 581, doi:10.3390/genes10080581.
113. 113. Frei, K.; Ramsebner, R.; Hamader, G.; Lucas, T.; Schoefer, C.; Baumgartner, W.D.; Wachtler, F.J.; Kirschhofer, K. Lack of association between Connexin 31 (GJB3) alterations and sensorineural deafness in Austria. *Hearing research* **2004**, *194*, 81-86, doi:10.1016/j.heares.2004.03.007.
114. 114. Gabriel, H.; Kupsch, P.; Sudendey, J.; Winterhager, E.; Jahnke, K.; Lautermann, J. Mutations in the connexin26/GJB2 gene are the most common event in non-syndromic hearing loss among the German population. *Human mutation* **2001**, *17*, 521-522, doi:10.1002/humu.1138.
115. 115. Gallant, E.; Francey, L.; Tsai, E.A.; Berman, M.; Zhao, Y.R.; Fetting, H.; Kaur, M.; Deardorff, M.A.; Wilkens, A.; Clark, D., et al. Homozygosity for the V37I GJB2 Mutation in Fifteen Probands With Mild to Moderate Sensorineural Hearing Impairment: Further Confirmation of Pathogenicity and Haplotype Analysis in Asian Populations. *American Journal of Medical Genetics Part A* **2013**, *161*, 2148-2157, doi:10.1002/ajmg.a.36042.
116. 116. Gao, Z.; Lu, Y.; Ke, J.; Li, T.; Hu, P.; Song, Y.; Xu, C.; Wang, J.; Cheng, J.; Zhang, L., et al. Application of SNPscan in Genetic Screening for Common Hearing Loss Genes. *PloS one* **2016**, *11*, e0165650, doi:10.1371/journal.pone.0165650.
117. 117. Gazzaz, B.; Weil, D.; Rais, L.; Akhyat, O.; Azeddoug, H.; Nadifi, S. Autosomal recessive and sporadic deafness in Morocco: high frequency of the 35delG GJB2 mutation and absence of the 342-kb GJB6 variant. *Hearing research* **2005**, *210*, 80-84, doi:10.1016/j.heares.2005.08.001.
118. 118. Gibriel, A.A.; Abou-Elew, M.H.; Masmoudi, S. Analysis of p.Gly12Valfs*2, p.Trp24* and p.Trp77Arg mutations in GJB2 and p.Arg81Gln variant in LRTOMT among non syndromic hearing loss Egyptian patients: implications for genetic diagnosis. *Mol Biol Rep* **2019**, *46*, 2139-2145, doi:10.1007/s11033-019-04667-0.
119. 119. Gravina, L.P.; Foncuberta, M.E.; Estrada, R.C.; Barreiro, C.; Chertkoff, L. Carrier frequency of the 35delG and A1555G deafness mutations in the Argentinean population. Impact on the newborn hearing screening. *International journal of pediatric otorhinolaryngology* **2007**, *71*, 639-643, doi:10.1016/j.ijporl.2006.12.015.

120. 120. Gravina, L.P.; Foncuberta, M.E.; Prieto, M.E.; Garrido, J.; Barreiro, C.; Chertkoff, L. Prevalence of DFNB1 mutations in Argentinean children with non-syndromic deafness. Report of a novel mutation in GJB2. *International journal of pediatric otorhinolaryngology* **2010**, *74*, 250-254, doi:10.1016/j.ijporl.2009.11.014.

121. 121. Gronskov, K.; Larsen, L.A.; Rendtorff, N.D.; Parving, A.; Norgaard-Pedersen, B.; Brondum-Nielsen, K. GJB2 and GJB6 mutations in 165 Danish patients showing non-syndromic hearing impairment. *Genet Test* **2004**, *8*, 181-184, doi:10.1089/gte.2004.8.181.

122. 122. Gualandi, F.; Ravani, A.; Berto, A.; Sensi, A.; Trabanelli, C.; Falciano, F.; Trevisi, P.; Mazzoli, M.; Tibiletti, M.G.; Cristofari, E., et al. Exploring the clinical and epidemiological complexity of GJB2-linked deafness. *American journal of medical genetics* **2002**, *112*, 38-45, doi:10.1002/ajmg.10621.

123. 123. Haack, B.; Schmalisch, K.; Palmada, M.; Bohmer, C.; Kohlschmidt, N.; Keilmann, A.; Zechner, U.; Limberger, A.; Beckert, S.; Zenner, H.P., et al. Deficient membrane integration of the novel p.N14D-GJB2 mutant associated with non-syndromic hearing impairment. *Human mutation* **2006**, *27*, 1158-1159, doi:10.1002/humu.9464.

124. 124. Haghighat-Nia, A.; Keivani, A.; Nadeali, Z.; Fazel-Najafabadi, E.; Hosseinzadeh, M.; Salehi, M. Mutation spectrum of autosomal recessive non-syndromic hearing loss in central Iran. *International journal of pediatric otorhinolaryngology* **2015**, *79*, 1892-1895, doi:10.1016/j.ijporl.2015.08.039.

125. 125. Hall, A.; Pembrey, M.; Lutman, M.; Steer, C.; Bitner-Glindzicz, M. Prevalence and audiological features in carriers of GJB2 mutations, c.35delG and c.101T>C (p.M34T), in a UK population study. *BMJ Open* **2012**, *2*, e001238, doi:10.1136/bmjopen-2012-001238.

126. 126. Hamelmann, C.; Amedofu, G.K.; Albrecht, K.; Muntau, B.; Gelhaus, A.; Brobby, G.W.; Horstmann, R.D. Pattern of connexin 26 (GJB2) mutations causing sensorineural hearing impairment in Ghana. *Human mutation* **2001**, *18*, 84-85, doi:10.1002/humu.1156.

127. 127. Hao, Z.; Fu, D.; Ming, Y.; Yang, J.; Huang, Q.; Lin, W.; Zhang, H.; Zhang, B.; Zhou, A.; Hu, X., et al. Large scale newborn deafness genetic screening of 142,417 neonates in Wuhan, China. *PloS one* **2018**, *13*, e0195740, doi:10.1371/journal.pone.0195740.

128. 128. Hashemi, S.B.; Ashraf, M.J.; Saboori, M.; Azarpira, N.; Darai, M. Prevalence of GJB2 (CX26) gene mutations in south Iranian patients with autosomal recessive nonsyndromic sensorineural hearing loss. *Mol Biol Rep* **2012**, *39*, 10481-10487, doi:10.1007/s11033-012-1929-9.

129. 129. Hayashi, C.; Funayama, M.; Li, Y.; Kamiya, K.; Kawano, A.; Suzuki, M.; Hattori, N.; Ikeda, K. Prevalence of GJB2 causing recessive profound non-syndromic deafness in Japanese children. *International journal of pediatric otorhinolaryngology* **2011**, *75*, 211-214, doi:10.1016/j.ijporl.2010.11.001.

130. 130. Hederstierna, C.; Möller, C.; Åhlman, H.; Lundberg, R.; Von Döbeln, U. The prevalence of Connexin 26 mutations in the Swedish population. *Audiological Medicine* **2005**, *3*, 154-157.

131. 131. Hismi, B.O.; Tokgoz-Yilmaz, S.; Incesulu, A.; Tekin, M. Effects of GJB2 genotypes on the audiological phenotype: Variability is present for all genotypes. *International journal of pediatric otorhinolaryngology* **2006**, *70*, 1687-1694, doi:10.1016/j.ijporl.2006.03.015.

132. 132. Hochman, J.B.; Stockley, T.L.; Shipp, D.; Lin, V.Y.; Chen, J.M.; Nedzelski, J.M. Prevalence of Connexin 26 (GJB2) and Pendred (SLC26A4) mutations in a population of adult cochlear implant candidates. *Otology & neurotology : official publication of the American Otological Society, American Neurotology Society [and] European Academy of Otology and Neurotology* **2010**, *31*, 919-922, doi:10.1097/MAO.0b013e3181e3d324.

133. 133. Houseman, M.J.; Ellis, L.A.; Pagnamenta, A.; Di, W.L.; Rickard, S.; Osborn, A.H.; Dahl, H.H.; Taylor, G.R.; Bitner-Glindzicz, M.; Reardon, W., et al. Genetic analysis of the connexin-26 M34T variant: identification of genotype M34T/M34T segregating with mild-moderate non-syndromic sensorineural hearing loss. *J Med Genet* **2001**, *38*, 20-25, doi:10.1136/jmg.38.1.20.

134. 134. Huang, B.; Han, M.; Wang, G.; Huang, S.; Zeng, J.; Yuan, Y.; Dai, P. Genetic mutations in non-syndromic deafness patients in Hainan Province have a different mutational spectrum compared to patients from Mainland China. *International journal of pediatric otorhinolaryngology* **2018**, *108*, 49-54, doi:10.1016/j.ijporl.2018.02.015.

135. 135. Huang, S.; Huang, B.; Wang, G.; Kang, D.Y.; Zhang, X.; Meng, X.; Dai, P. The relationship between the GJB3 c.538C>T variant and hearing phenotype in the Chinese population. *International journal of pediatric otorhinolaryngology* **2017**, *102*, 67-70, doi:10.1016/j.ijporl.2017.09.001.

136. 136. Huang, S.S.; Huang, B.Q.; Wang, G.J.; Yuan, Y.Y.; Dai, P. The Relationship between the p.V37I Mutation in GJB2 and Hearing Phenotypes in Chinese Individuals. *PloS one* **2015**, *10*, e0129662, doi:ARTN e0129662

137. 10.1371/journal.pone.0129662.

138. 137. Huang, Y.; Yang, X.L.; Chen, W.X.; Duan, B.; Lu, P.; Wang, Y.; Xu, Z.M. Prevalence of p.V37I variant of GJB2 among Chinese infants with mild or moderate hearing loss. *International Journal of Clinical and Experimental Medicine* **2015**, *8*, 21674-21678.

139. 138. Huculak, C.; Bruyere, H.; Nelson, T.N.; Kozak, F.K.; Langlois, S. V37I connexin 26 allele in patients with sensorineural hearing loss: evidence of its pathogenicity. *American journal of medical genetics. Part A* **2006**, *140*, 2394-2400, doi:10.1002/ajmg.a.31486.

140. 139. Hwa, H.L.; Ko, T.M.; Hsu, C.J.; Huang, C.H.; Chiang, Y.L.; Oong, J.L.; Chen, C.C.; Hsu, C.K. Mutation spectrum of the connexin 26 (GJB2) gene in Taiwanese patients with prelingual deafness. *Genet Med* **2003**, *5*, 161-165, doi:10.1097/01.GIM.0000066796.11916.94.

141. 140. Iliadou, V.; Eleftheriades, N.; Metaxas, A.S.; Skevas, A.; Kiratzidis, T.; Pampanos, A.; Voyiatzis, N.; Grigoriadou, M.; Petersen, M.B.; Iliades, T. Audiological profile of the prevalent genetic form of childhood sensorineural hearing loss due to GJB2 mutations in northern Greece. *European Archives of Oto-Rhino-Laryngology* **2004**, *261*, 259-261, doi:10.1007/s00405-003-0679-7.

142. 141. Imtiaz, F.; Taibah, K.; Ramzan, K.; Bin-Khamis, G.; Kennedy, S.; Al-Mubarak, B.; Trabzuni, D.; Allam, R.; Al-Mostafa, A.; Sogaty, S., et al. A comprehensive introduction to the genetic basis of non-syndromic hearing loss in the Saudi Arabian population. *BMC Med Genet* **2011**, *12*, 91, doi:10.1186/1471-2350-12-91.

143. 142. Jabbarpour Bonyadi, M.; Esmaeili, M.; Younespour, R.; Lotfalizadeh, N.; Absavaran, A. Analysis of Common Mutations in GJB2 and GJB6 Genes in Patients with Autosomal Recessive Non-syndromic Hearing Loss in Eastern Azarbaijan. *J Adv Med Biomed Res* **2006**, *14*, 30-38.

144. 143. Janecke, A.R.; Hirst-Stadlmann, A.; Gunther, B.; Utermann, B.; Muller, T.; Loffler, J.; Utermann, G.; Nekahm-Heis, D. Progressive hearing loss, and recurrent sudden sensorineural hearing loss associated with GJB2 mutations--phenotypic spectrum and frequencies of GJB2 mutations in Austria. *Human genetics* **2002**, *111*, 145-153, doi:10.1007/s00439-002-0762-y.

145. 144. Jiang, H.; Chen, J.; Shan, X.J.; Li, Y.; He, J.G.; Yang, B.B. Prevalence and range of GJB2 and SLC26A4 mutations in patients with autosomal recessive nonsyndromic hearing loss. *Molecular medicine reports* **2014**, *10*, 379-386, doi:10.3892/mmr.2014.2148.

146. 145. Jiang, H.; Liu, Q.; Chen, L. Screening and analysis of mutation hot-spots in deafness-associated genes among adolescents with hearing loss. *Molecular medicine reports* **2015**, *12*, 8179-8184, doi:10.3892/mmr.2015.4475.
147. 146. Jiang, H.; Niu, Y.; Qu, L.; Huang, X.; Zhu, X.; Tang, G. A novel compound heterozygous mutation in the GJB2 gene is associated with non-syndromic hearing loss in a Chinese family. *Biosci Trends* **2018**, *12*, 470-475, doi:10.5582/bst.2018.01156.
148. 147. Jiang, Y.; Huang, S.; Deng, T.; Wu, L.; Chen, J.; Kang, D.; Xu, X.; Li, R.; Han, D.; Dai, P. Mutation Spectrum of Common Deafness-Causing Genes in Patients with Non-Syndromic Deafness in the Xiamen Area, China. *PloS one* **2015**, *10*, e0135088, doi:10.1371/journal.pone.0135088.
149. 148. Jin, P.; Yu, S.; Zhu, W.; Tang, Y.; Du, B.; Wang, P. [Study on the causes of deafness in a consanguineous marriage family using DNA microarray]. *Lin chuang er bi yan hou tou jing wai ke za zhi = Journal of clinical otorhinolaryngology, head, and neck surgery* **2011**, *25*, 165-168.
150. 149. Joseph, A.Y.; Rasool, T.J. High frequency of connexin26 (GJB2) mutations associated with nonsyndromic hearing loss in the population of Kerala, India. *International journal of pediatric otorhinolaryngology* **2009**, *73*, 437-443, doi:10.1016/j.ijporl.2008.11.010.
151. 150. Jung, J.; Lee, J.S.; Cho, K.J.; Yu, S.; Yoon, J.H.; Yung Gee, H.; Choi, J.Y. Genetic Predisposition to Sporadic Congenital Hearing Loss in a Pediatric Population. *Scientific reports* **2017**, *7*, 45973, doi:10.1038/srep45973.
152. 151. Kaheel, H.; Bress, A.; Hassan, M.A.; Shah, A.A.; Amin, M.; Bakhit, Y.H.Y.; Kniper, M. Frequency of c.35delG Mutation in GJB2 Gene (Connexin 26) in Syrian Patients with Nonsyndromic Hearing Impairment. *Genet Res Int* **2017**, *2017*, 5836525, doi:10.1155/2017/5836525.
153. 152. Kalay, E.; Caylan, R.; Kremer, H.; de Brouwer, A.P.; Karaguzel, A. GJB2 mutations in Turkish patients with ARNSHL: prevalence and two novel mutations. *Hearing research* **2005**, *203*, 88-93, doi:10.1016/j.heares.2004.11.022.
154. 153. Karamert, R.; Bayazit, Y.A.; Altinyay, S.; Yilmaz, A.; Menevse, A.; Gokdogan, O.; Gokdogan, C.; Ant, A. Association of GJB2 gene mutation with cochlear implant performance in genetic non-syndromic hearing loss. *International journal of pediatric otorhinolaryngology* **2011**, *75*, 1572-1575, doi:10.1016/j.ijporl.2011.09.010.
155. 154. Kashef, A.; Nikzat, N.; Bazzazadegan, N.; Fattahi, Z.; Sabbagh-Kermani, F.; Taghdiri, M.; Azadeh, B.; Mojahedi, F.; Khoshaeen, A.; Habibi, H., et al. Finding mutation within non-coding region of GJB2 reveals its importance in genetic testing of hearing loss in Iranian population. *International journal of pediatric otorhinolaryngology* **2015**, *79*, 136-138, doi:10.1016/j.ijporl.2014.11.024.
156. 155. Kecskemeti, N.; Szonyi, M.; Gaborjan, A.; Kustel, M.; Milley, G.M.; Suveges, A.; Illes, A.; Kekesi, A.; Tamas, L.; Molnar, M.J., et al. Analysis of GJB2 mutations and the clinical manifestation in a large Hungarian cohort. *European archives of oto-rhino-laryngology : official journal of the European Federation of Oto-Rhino-Laryngological Societies* **2018**, *275*, 2441-2448, doi:10.1007/s00405-018-5083-4.
157. 156. Kenna, M.A.; Feldman, H.A.; Neault, M.W.; Frangulov, A.; Wu, B.L.; Fligor, B.; Rehm, H.L. Audiologic phenotype and progression in GJB2 (Connexin 26) hearing loss. *Archives of otolaryngology--head & neck surgery* **2010**, *136*, 81-87, doi:10.1001/archoto.2009.202.
158. 157. Kenna, M.A.; Wu, B.L.; Cotanche, D.A.; Korf, B.R.; Rehm, H.L. Connexin 26 studies in patients with sensorineural hearing loss. *Archives of otolaryngology--head & neck surgery* **2001**, *127*, 1037-1042, doi:10.1001/archotol.127.9.1037.

159. 158. Khandelwal, G.; Bhalla, S.; Khullar, M.; Panda, N.K. High frequency of heterozygosity in GJB2 mutations among patients with non-syndromic hearing loss. *The Journal of laryngology and otology* **2009**, *123*, 273-277, doi:10.1017/s0022215108002892.

160. 159. Khidiiatova, I.M.; Dzhemileva, L.U.; Khabibulin, R.M.; Khusnutdinova, E.K. [Frequency of the 35delG mutation of the connexin 26 gene (GJB2) in patients with non-syndromic autosome-recessive deafness from Bashkortostan and in ethnic groups of the Volga-Ural region]. *Molekuliarnaia biologiia* **2002**, *36*, 438-441.

161. 160. Kim, B.J.; Kim, A.R.; Park, G.; Park, W.Y.; Chang, S.O.; Oh, S.H.; Choi, B.Y. Targeted Exome Sequencing of Deafness Genes After Failure of Auditory Phenotype-Driven Candidate Gene Screening. *Otology & neurotology : official publication of the American Otological Society, American Neurotology Society [and] European Academy of Otology and Neurotology* **2015**, *36*, 1096-1102, doi:10.1097/mao.0000000000000747.

162. 161. Kim, H.J.; Park, C.H.; Kim, H.J.; Lee, K.O.; Won, H.H.; Ko, M.H.; Chu, H.; Cho, Y.S.; Chung, W.H.; Kim, J.W., et al. Sequence Variations and Haplotypes of the GJB2 Gene Revealed by Resequencing of 192 Chromosomes from the General Population in Korea. *Clin Exp Otorhinolaryngol* **2010**, *3*, 65-69, doi:10.3342/ceo.2010.3.2.65.

163. 162. Kim, J.; Jung, J.; Lee, M.G.; Choi, J.Y.; Lee, K.A. Non-syndromic hearing loss caused by the dominant cis mutation R75Q with the recessive mutation V37I of the GJB2 (Connexin 26) gene. *Experimental & molecular medicine* **2015**, *47*, e169, doi:10.1038/emm.2015.32.

164. 163. Kim, S.Y.; Kim, A.R.; Han, K.H.; Kim, M.Y.; Jeon, E.H.; Koo, J.W.; Oh, S.H.; Choi, B.Y. Residual Hearing in DFNB1 Deafness and Its Clinical Implication in a Korean Population. *PloS one* **2015**, *10*, e0125416, doi:10.1371/journal.pone.0125416.

165. 164. Kim, S.Y.; Kim, A.R.; Kim, N.K.; Lee, C.; Kim, M.Y.; Jeon, E.H.; Park, W.Y.; Choi, B.Y. Unraveling of Enigmatic Hearing-Impaired GJB2 Single Heterozygotes by Massive Parallel Sequencing: DFNB1 or Not? *Medicine (Baltimore)* **2016**, *95*, e3029, doi:10.1097/MD.0000000000003029.

166. 165. Kim, S.Y.; Park, G.; Han, K.H.; Kim, A.; Koo, J.W.; Chang, S.O.; Oh, S.H.; Park, W.Y.; Choi, B.Y. Prevalence of p.V37I variant of GJB2 in mild or moderate hearing loss in a pediatric population and the interpretation of its pathogenicity. *PloS one* **2013**, *8*, e61592, doi:10.1371/journal.pone.0061592.

167. 166. Kokotas, H.; Grigoriadou, M.; Yang, L.; Lodahl, M.; Rendtorff, N.D.; Gyftodimou, Y.; Korres, G.S.; Ferekidou, E.; Kandiloros, D.; Korres, S., et al. Homoplasmy of the G7444A mtDNA and heterozygosity of the GJB2 c.35delG mutations in a family with hearing loss. *International journal of pediatric otorhinolaryngology* **2011**, *75*, 89-94, doi:10.1016/j.ijporl.2010.10.016.

168. 167. Koohiyan, M.; Hashemzadeh-Chaleshtori, M.; Salehi, M.; Abtahi, H.; Reiisi, S.; Pourreza, M.R.; Noori-Daloii, M.R.; Tabatabaiefar, M.A. GJB2 mutations causing autosomal recessive non-syndromic hearing loss (ARNSHL) in two Iranian populations: Report of two novel variants. *International journal of pediatric otorhinolaryngology* **2018**, *107*, 121-126, doi:10.1016/j.ijporl.2018.01.012.

169. 168. Kooshavar, D.; Tabatabaiefar, M.A.; Farrokhi, E.; Abolhasani, M.; Noori-Daloii, M.R.; Hashemzadeh-Chaleshtori, M. Digenic inheritance in autosomal recessive non-syndromic hearing loss cases carrying GJB2 heterozygote mutations: assessment of GJB4, GJA1, and GJC3. *International journal of pediatric otorhinolaryngology* **2013**, *77*, 189-193, doi:10.1016/j.ijporl.2012.10.015.

170. 169. Kudo, T.; Ikeda, K.; Kure, S.; Matsubara, Y.; Oshima, T.; Watanabe, K.; Kawase, T.; Narisawa, K.; Takasaka, T. Novel mutations in the connexin 26 gene (GJB2) responsible for childhood deafness in the Japanese population. *American journal of medical genetics* **2000**, *90*, 141-145, doi:10.1002/(sici)1096-8628(20000117)90:2<141::aid-ajmg10>3.0.co;2-g.

171. 170. Kudo, T.; Ikeda, K.; Oshima, T.; Kure, S.; Tammasaeng, M.; Prasansuk, S.; Matsubara, Y. GJB2 (connexin 26) mutations and childhood deafness in Thailand. *Otology & neurotology : official publication of the American Otological Society, American Neurotology Society [and] European Academy of Otology and Neurotology* **2001**, *22*, 858-861, doi:10.1097/00129492-200111000-00025.

172. 171. Kupka, S.; Braun, S.; Aberle, S.; Haack, B.; Ebauer, M.; Zeißler, U.; Zenner, H.P.; Blin, N.; Pfister, M. Frequencies of GJB2 mutations in German control individuals and patients showing sporadic non - syndromic hearing impairment. *Human mutation* **2002**, *20*, 77-78.

173. 172. Lameiras, A.R.; Goncalves, A.C.; Santos, R.; O'Neill, A.; Reis, L.R.; Matos, T.D.; Fialho, G.; Caria, H.; Escada, P. The controversial p.Met34Thr variant in GJB2 gene: Two siblings, one genotype, two phenotypes. *International journal of pediatric otorhinolaryngology* **2015**, *79*, 1316-1319, doi:10.1016/j.ijporl.2015.05.041.

174. 173. Leclere, J.C.; Le Gac, M.S.; Le Marechal, C.; Ferec, C.; Marianowski, R. GJB2 mutations: Genotypic and phenotypic correlation in a cohort of 690 hearing-impaired patients, toward a new mutation? *International journal of pediatric otorhinolaryngology* **2017**, *102*, 80-85, doi:10.1016/j.ijporl.2017.09.011.

175. 174. Lee, K.Y.; Choi, S.Y.; Bae, J.W.; Kim, S.; Chung, K.W.; Drayna, D.; Kim, U.K.; Lee, S.H. Molecular analysis of the GJB2, GJB6 and SLC26A4 genes in Korean deafness patients. *International journal of pediatric otorhinolaryngology* **2008**, *72*, 1301-1309, doi:10.1016/j.ijporl.2008.05.007.

176. 175. Lerer, I.; Sagi, M.; Ben-Neriah, Z.; Wang, T.; Levi, H.; Abeliovich, D. A deletion mutation in GJB6 cooperating with a GJB2 mutation in trans in non-syndromic deafness: A novel founder mutation in Ashkenazi Jews. *Human mutation* **2001**, *18*, 460, doi:10.1002/humu.1222.

177. 176. Li, C.; Lu, D.; Chen, X.; Huang, B.; Chen, K.; Liu, X.; Hu, A.; Zhang, Y.; Xue, X.; Xing, Y., et al. [Analysis of mutations of 4 common genes among 216 patients with non-syndromic hearing impairment]. *Zhonghua yi xue yi chuan xue za zhi = Zhonghua yixue yichuanxue zazhi = Chinese journal of medical genetics* **2018**, *35*, 630-633, doi:10.3760/cma.j.issn.1003-9406.2018.05.002.

178. 177. Li, H.; Li, H.B.; Mao, J.; Liu, M.J.; Cheng, H.B.; Li, S.L.; Chen, Y. [Genetic screening of mutation hot-spots for nonsyndromic hearing loss in southern Jiangsu province with SNaPshot]. *Zhonghua yi xue yi chuan xue za zhi = Zhonghua yixue yichuanxue zazhi = Chinese journal of medical genetics* **2011**, *28*, 383-386, doi:10.3760/cma.j.issn.1003-9406.2011.04.005.

179. 178. Li, Q.; Fang, R.P.; Zhou, H.G.; Dai, P.; Tian, L.; Lin, D.; Huang, Q.; Song, J.M. [GJB2 235delC single allelic mutation modulates the phenotype associated with the mitochondrial A1555G mutation]. *Zhonghua yi xue yi chuan xue za zhi = Zhonghua yixue yichuanxue zazhi = Chinese journal of medical genetics* **2010**, *27*, 194-197, doi:10.3760/cma.j.issn.1003-9406.2010.02.017.

180. 179. Lim, L.H.; Bradshaw, J.K.; Guo, Y.; Pilipenko, V.; Madden, C.; Ingala, D.; Keddache, M.; Choo, D.I.; Wenstrup, R.; Greinwald, J.H., Jr. Genotypic and phenotypic correlations of DFNB1-related hearing impairment in the Midwestern United States. *Archives of otolaryngology--head & neck surgery* **2003**, *129*, 836-840, doi:10.1001/archotol.129.8.836.

181. 180. Lin, D.; Goldstein, J.A.; Mhatre, A.N.; Lustig, L.R.; Pfister, M.; Lalwani, A.K. Assessment of denaturing high-performance liquid chromatography (DHPLC) in screening for mutations in connexin 26 (GJB2). *Human mutation* **2001**, *18*, 42-51, doi:10.1002/humu.1148.

182. 181. Liu, S.; Xu, L.; Chen, B.; Liu, M.; Qu, S.; Liang, J.; Tang, F.; Shi, M.; Peng, L.; Jing, Y., et al. [Analysis common gene mutation spots of 127 non-syndromic deafness natients in Guangxi Drovince]. *Lin chuang er bi yan hou tou jing wai ke za zhi = Journal of clinical otorhinolaryngology, head, and neck surgery* **2015**, *29*, 1954-1958.

183. 182. Liu, X.Z.; Xia, X.J.; Ke, X.M.; Ouyang, X.M.; Du, L.L.; Liu, Y.H.; Angeli, S.; Telischi, F.F.; Nance, W.E.; Balkany, T., et al. The prevalence of connexin 26 (GJB2) mutations in the Chinese population. *Human genetics* **2002**, *111*, 394-397, doi:10.1007/s00439-002-0811-6.

184. 183. Liu, X.Z.; Yuan, Y.; Yan, D.; Ding, E.H.; Ouyang, X.M.; Fei, Y.; Tang, W.; Yuan, H.; Chang, Q.; Du, L.L., et al. Digenic inheritance of non-syndromic deafness caused by mutations at the gap junction proteins Cx26 and Cx31. *Human genetics* **2009**, *125*, 53-62, doi:10.1007/s00439-008-0602-9.

185. 184. Loeza-Becerra, F.; Rivera-Vega Mdel, R.; Martinez-Saucedo, M.; Gonzalez-Huerta, L.M.; Urueta-Cuellar, H.; Berrruecos-Villalobos, P.; Cuevas-Covarrubias, S. Particular distribution of the GJB2/GJB6 gene mutations in Mexican population with hearing impairment. *International journal of pediatric otorhinolaryngology* **2014**, *78*, 1057-1060, doi:10.1016/j.ijporl.2014.04.002.

186. 185. Lopponen, T.; Vaisanen, M.L.; Luotonen, M.; Allinen, M.; Uusimaa, J.; Lindholm, P.; Maki-Torkko, E.; Vayrynen, M.; Lopponen, H.; Leisti, J. Connexin 26 mutations and nonsyndromic hearing impairment in Northern Finland. *The Laryngoscope* **2003**, *113*, 1758-1763.

187. 186. Ma, Y.; Xiao, Y.; Bai, X.; Zhang, F.; Zhang, D.; Xu, X.; Xu, L.; Wang, H. GJB2, SLC26A4, and mitochondrial DNA12S rRNA hot-spots in 156 subjects with non-syndromic hearing loss in Tengzhou, China. *Acta oto-laryngologica* **2016**, *136*, 800-805, doi:10.3109/00016489.2016.1164893.

188. 187. Mahasneh, A.; Battah, R. Prevalence of connexin 26 mutations in patients from Jordan with non syndromic hearing loss. *International Journal of Human Genetics* **2006**, *6*, 119-124.

189. 188. Mahayri, Z.N.; Monem, F.S. GJB2 gene mutations in Syrians with sensorineural hearing loss. *Middle East Journal of Medical Genetics* **2012**, *1*, 80-84.

190. 189. Mahdieh, N.; Mahmoudi, H.; Ahmadzadeh, S.; Bakhtiyari, S. GJB2 mutations in deaf population of Ilam (Western Iran): a different pattern of mutation distribution. *European archives of oto-rhino-laryngology : official journal of the European Federation of Oto-Rhino-Laryngological Societies* **2016**, *273*, 1161-1165, doi:10.1007/s00405-015-3684-8.

191. 190. Mahdieh, N.; Nishimura, C.; Ali-Madadi, K.; Riazalhosseini, Y.; Yazdan, H.; Arzhangi, S.; Jalalvand, K.; Ebrahimi, A.; Kazemi, S.; Smith, R.J., et al. The frequency of GJB2 mutations and the Delta (GJB6-D13S1830) deletion as a cause of autosomal recessive non-syndromic deafness in the Kurdish population. *Clinical genetics* **2004**, *65*, 506-508, doi:10.1111/j.1399-0004.2004.00262.x.

192. 191. Mahdieh, N.; Rabbani, B.; Shirkavand, A.; Bagherian, H.; Movahed, Z.S.; Fouladi, P.; Rahiminejad, F.; Masoudifard, M.; Akbari, M.T.; Zeinali, S. Impact of consanguineous marriages in GJB2-related hearing loss in the Iranian population: a report of a novel variant. *Genet Test Mol Biomarkers* **2011**, *15*, 489-493, doi:10.1089/gtmb.2010.0145.

193. 192. Mahdieh, N.; RIAZ, A.Y.; Arzhangi, S.; Bazazzadegan, N.; TOUTOUNCHI, M.; NAJMABAD, H.; NISHIMURA, C.; SMITH, R.; ALI, M.K.; YAZDAN, Y. Frequency of connexin26 gene mutations in autosomal recessive non-syndromic deafness in kermanshah (2002-4). **2005**.

194. 193. Maheshwari, M.; Vijaya, R.; Ghosh, M.; Shastri, S.; Kabra, M.; Menon, P.S. Screening of families with autosomal recessive non-syndromic hearing impairment (ARNSHI) for mutations in GJB2 gene: Indian scenario. *American journal of medical genetics. Part A* **2003**, *120a*, 180-184, doi:10.1002/ajmg.a.20014.

195. 194. Man, R.; Zhang, Z.; Lu, R.; Wang, X.; Sun, S.; Wang, D.; Xu, X.; Wang, W.; Wang, H. [Mutation analysis of 16 mutation spots related to children patients with non-syndromic sensorineural hearing loss]. *Lin chuang er bi yan hou tou jing wai ke za zhi = Journal of clinical otorhinolaryngology, head, and neck surgery* **2015**, *29*, 319-324.

196. 195. Mani, R.S.; Ganapathy, A.; Jalvi, R.; Srikumari Srisailapathy, C.R.; Malhotra, V.; Chadha, S.; Agarwal, A.; Ramesh, A.; Rangasayee, R.R.; Anand, A. Functional consequences of novel connexin 26 mutations associated with hereditary hearing loss. *European journal of human genetics : EJHG* **2009**, *17*, 502-509, doi:10.1038/ejhg.2008.179.

197. 196. Manzoli, G.N.; Abe-Sandes, K.; Bittles, A.H.; da Silva, D.S.; Fernandes Lda, C.; Paulon, R.M.; de Castro, I.C.; Padovani, C.M.; Acosta, A.X. Non-syndromic hearing impairment in a multi-ethnic population of Northeastern Brazil. *International journal of pediatric otorhinolaryngology* **2013**, *77*, 1077-1082, doi:10.1016/j.ijporl.2013.04.001.

198. 197. Marlin, S.; Feldmann, D.; Blons, H.; Loundon, N.; Rouillon, I.; Albert, S.; Chauvin, P.; Garabedian, E.N.; Couderc, R.; Odent, S., et al. GJB2 and GJB6 mutations: genotypic and phenotypic correlations in a large cohort of hearing-impaired patients. *Archives of otolaryngology--head & neck surgery* **2005**, *131*, 481-487, doi:10.1001/archotol.131.6.481.

199. 198. Marlin, S.; Garabedian, E.N.; Roger, G.; Moatti, L.; Matha, N.; Lewin, P.; Petit, C.; Denoyelle, F. Connexin 26 gene mutations in congenitally deaf children: pitfalls for genetic counseling. *Archives of otolaryngology--head & neck surgery* **2001**, *127*, 927-933, doi:10.1001/archotol.127.8.927.

200. 199. Martínez-Saucedo, M.; Rivera-Vega, M.; Gonzalez-Huerta, L.; Urueta-Cuéllar, H.; Cuevas-Covarrubias, S. Exome sequencing analysis reveals homozygous GJB2 gene mutation in a Mexican family with profound hearing loss. *Revista Médica del Hospital General de México* **2017**, *80*, 77-80.

201. 200. MASMOUDI, S.; ELGAIED-BOULILA, A.; KASSAB, I.; ARAB, S.B.; BLANCHARD, S.; BOUZOUITA, J.-E.; DRIRA, M.; KASSAB, A.; HACHICHA, S.; PETIT, C. Determination of the frequency of connexin26 mutations in inherited sensorineural deafness and carrier rates in the Tunisian population using DGGE. *Journal of medical genetics* **2000**, *37*, e39-e39.

202. 201. Matos, T.D.; Simoes-Teixeira, H.; Caria, H.; Rosa, H.; O'Neill, A.; Fialho, G. The controversial p.Arg127His mutation in GJB2: report on three Portuguese hearing loss family cases. *Genet Test Mol Biomarkers* **2010**, *14*, 141-144, doi:10.1089/gtmb.2009.0103.

203. 202. Medica, I.; Rudolf, G.; Balaban, M.; Peterlin, B. C. 35delG/GJB2 and del (GJB6-D13S1830) mutations in Croatians with prelingual non-syndromic hearing impairment. *BMC Ear, Nose and Throat Disorders* **2005**, *5*, 11.

204. 203. Medica, I.; Rudolf, G.; Prpic, I.; Stanojevic, M.; Peterlin, B. Incidence of the del35G/GJB2 mutation in Croatian newborns with hearing impairment. *Medical science monitor : international medical journal of experimental and clinical research* **2005**, *11*, CR533-535.

205. 204. Medlej-Hashim, M.; Mustapha, M.; Chouery, E.; Weil, D.; Parronaud, J.; Salem, N.; Delague, V.; Loiselet, J.; Lathrop, M.; Petit, C., et al. Non-syndromic recessive deafness in Jordan: mapping of a new locus to chromosome 9q34.3 and prevalence of DFNB1 mutations. *European journal of human genetics : EJHG* **2002**, *10*, 391-394, doi:10.1038/sj.ejhg.5200813.

206. 205. Melo, U.S.; Santos, S.; Cavalcanti, H.G.; Andrade, W.T.; Dantas, V.G.; Rosa, M.R.; Mingroni-Netto, R.C. Strategies for genetic study of hearing loss in the Brazilian northeastern region. *Int J Mol Epidemiol Genet* **2014**, *5*, 11-21.

207. 206. Meyer, N.C.; Nishimura, C.J.; McMordie, S.; Smith, R.J. Audioprofiling identifies TECTA and GJB2-related deafness segregating in a single extended pedigree. *Clinical genetics* **2007**, *72*, 130-137, doi:10.1111/j.1399-0004.2007.00828.x.

208. 207. Milunsky, J.M.; Maher, T.A.; Yosunkaya, E.; Vohr, B.R. Connexin-26 gene analysis in hearing-impaired newborns. *Genet Test* **2000**, *4*, 345-349, doi:10.1089/109065700750065072.

209. 208. Minarik, G.; Ferak, V.; Ferakova, E.; Ficek, A.; Polakova, H.; Kadasi, L. High frequency of GJB2 mutation W24X among Slovak Romany (Gypsy) patients with non-syndromic hearing loss (NSHL). *General physiology and biophysics* **2003**, *22*, 549-556.

210. 209. Mishra, S.; Pandey, H.; Srivastava, P.; Mandal, K.; Phadke, S.R. Connexin 26 (GJB2) Mutations Associated with Non-Syndromic Hearing Loss (NSHL). *Indian journal of pediatrics* **2018**, *85*, 1061-1066, doi:10.1007/s12098-018-2654-8.

211. 210. Moctar, E.C.; Riahi, Z.; El Hachmi, H.; Veten, F.; Meiloud, G.; Bonnet, C.; Abdelhak, S.; Errami, M.; Houmeida, A. Etiology and associated GJB2 mutations in Mauritanian children with non-syndromic hearing loss. *European archives of oto-rhino-laryngology : official journal of the European Federation of Oto-Rhino-Laryngological Societies* **2016**, *273*, 3693-3698, doi:10.1007/s00405-016-4036-z.

212. 211. Mohamed, M.R.; Alesutan, I.; Foller, M.; Sopjani, M.; Bress, A.; Baur, M.; Salama, R.H.; Bakr, M.S.; Mohamed, M.A.; Blin, N., et al. Functional analysis of a novel I71N mutation in the GJB2 gene among Southern Egyptians causing autosomal recessive hearing loss. *Cellular physiology and biochemistry : international journal of experimental cellular physiology, biochemistry, and pharmacology* **2010**, *26*, 959-966, doi:10.1159/000324004.

213. 212. Moreira, D.; Silva, D.; Lopez, P.; Mantovani, J.C. Screening of connexin 26 in nonsyndromic hearing loss. *International archives of otorhinolaryngology* **2015**, *19*, 30-33, doi:10.1055/s-0034-1373783.

214. 213. Murgia, A.; Orzan, E.; Polli, R.; Martella, M.; Vinanzi, C.; Leonardi, E.; Arslan, E.; Zacchello, F. Cx26 deafness: mutation analysis and clinical variability. *J Med Genet* **1999**, *36*, 829-832.

215. 214. Naddafnia, H.; Noormohammadi, Z.; Irani, S.; Salahshoorifar, I. Frequency of GJB2 mutations, GJB6-D13S1830 and GJB6-D13S1854 deletions among patients with non-syndromic hearing loss from the central region of Iran. *Mol Genet Genomic Med* **2019**, *7*, e00780, doi:10.1002/mgg3.780.

216. 215. Najmabadi, H.; Cucci, R.A.; Sahebjam, S.; Kouchakian, N.; Farhadi, M.; Kahrizi, K.; Arzhangi, S.; Daneshmandan, N.; Javan, K.; Smith, R.J. GJB2 mutations in Iranians with autosomal recessive non-syndromic sensorineural hearing loss. *Human mutation* **2002**, *19*, 572, doi:10.1002/humu.9033.

217. 216. Nayyar, S.; Mukherjee, S.; Moorchung, N.; James, E.; Venkatesh, M.; Sukthankar, P.; Sabarigirish, K.; Batra, R. Connexin 26 mutations in congenital SNHL in Indian population. *Indian Journal of Otology* **2011**, *17*, 145.

218. 217. Neocleous, V.; Costi, C.; Shammas, C.; Spanou, E.; Anastasiadou, V.; Tanteles, G.A.; Phylactou, L.A. Spectrum of GJB2 mutations in Cypriot nonsyndromic hearing loss subjects. *J Genet* **2014**, *93*, 471-476, doi:10.1007/s12041-014-0365-0.

219. 218. Ohtsuka, A.; Yuge, I.; Kimura, S.; Namba, A.; Abe, S.; Van Laer, L.; Van Camp, G.; Usami, S. GJB2 deafness gene shows a specific spectrum of mutations in Japan, including a frequent founder mutation. *Human genetics* **2003**, *112*, 329-333, doi:10.1007/s00439-002-0889-x.

220. 219. Oliveira, C.A.; Maciel-Guerra, A.T.; Sartorato, E.L. Deafness resulting from mutations in the GJB2 (connexin 26) gene in Brazilian patients. *Clinical genetics* **2002**, *61*, 354-358, doi:10.1034/j.1399-0004.2002.610506.x.

221. 220. Onsori, H. A case report of non-syndromic sensorineural hearing loss with a compound heterozygous mutation (35delG/del120E) in the GJB2 gene. *The Journal of Qazvin University of Medical Sciences* **2016**, *20*, 70-74.

222. 221. Onsori, H.; Rahmati, M.; Fazli, D. A novel compound heterozygous mutation (35delG, 363delC) in the Connexin 26 gene causes non-syndromic autosomal recessive hearing loss. *Acta medica Iranica* **2014**, *52*, 638-640.

223. 222. Orzan, E.; Murgia, A.; Polli, R.; Martella, M.; Mazza, A.; Zacchello, F.; Babighian, G. Connexin 26 preverbal hearing impairment: mutation prevalence and heterozygosity in a selected population. *International journal of audiology* **2002**, *41*, 120-124, doi:10.3109/14992020209090402.

224. 223. Pagarkar, W.; Bitner-Glindzicz, M.; Knight, J.; Sirimanna, T. Late postnatal onset of hearing loss due to GJB2 mutations. *International journal of pediatric otorhinolaryngology* **2006**, *70*, 1119-1124, doi:10.1016/j.ijporl.2005.10.026.

225. 224. Pallares-Ruiz, N.; Blanchet, P.; Mondain, M.; Claustres, M.; Roux, A.F. A large deletion including most of GJB6 in recessive non syndromic deafness: a digenic effect? *European journal of human genetics : EJHG* **2002**, *10*, 72-76, doi:10.1038/sj.ejhg.5200762.

226. 225. Pandya, A.; Arnos, K.S.; Xia, X.J.; Welch, K.O.; Blanton, S.H.; Friedman, T.B.; Garcia Sanchez, G.; Liu, M.X.; Morell, R.; Nance, W.E. Frequency and distribution of GJB2 (connexin 26) and GJB6 (connexin 30) mutations in a large North American repository of deaf probands. *Genet Med* **2003**, *5*, 295-303, doi:10.1097/01.GIM.0000078026.01140.68.

227. 226. Park, H.J.; Hahn, S.H.; Chun, Y.M.; Park, K.; Kim, H.N. Connexin26 mutations associated with nonsyndromic hearing loss. *The Laryngoscope* **2000**, *110*, 1535-1538, doi:10.1097/00005537-200009000-00023.

228. 227. Paz-y-Mino, C.; Beaty, D.; Lopez-Cortes, A.; Proano, I. Frequency of GJB2 and del(GJB6-D13S1830) mutations among an Ecuadorian mestizo population. *International journal of pediatric otorhinolaryngology* **2014**, *78*, 1648-1654, doi:10.1016/j.ijporl.2014.07.014.

229. 228. Peng, X.; Li, X.; Xu, L.; Guan, B.; Zhang, J.; Yu, A. [Molecular etiology analysis among students with profound hearing loss in a special education school in Yangzhou]. *Lin chuang er bi yan hou tou jing wai ke za zhi = Journal of clinical otorhinolaryngology, head, and neck surgery* **2012**, *26*, 577-580.

230. 229. Petersen, M.B.; Grigoriadou, M.; Economides, J.; Kokotas, H. Compound heterozygosity of the novel c.292C>T (p.R98W) and the c.35delG GJB2 mutations in postlingual, non-syndromic, sensorineural deafness. *International journal of pediatric otorhinolaryngology* **2012**, *76*, 549-551, doi:10.1016/j.ijporl.2012.01.014.

231. 230. Petersen, M.B.; Grigoriadou, M.; Koutroumpe, M.; Kokotas, H. The novel c.247_249delTTC (p.F83del) GJB2 mutation in a family with prelingual sensorineural deafness. *International journal of pediatric otorhinolaryngology* **2012**, *76*, 969-971, doi:10.1016/j.ijporl.2012.03.007.

232. 231. Peyvandi, A.; Morovvati, S.; Rabiee, H.; Ranjbar, R.; Ajalloueyan, M.; Hassanalifard, M. Detection of the GJB2 Mutation in Iranian Children with Hearing Loss Treated with Cochlear Implantation. *Balkan journal of medical genetics : BJMG* **2011**, *14*, 19-24, doi:10.2478/v10034-011-0013-4.

233. 232. Picciotti, P.M.; Pietrobono, R.; Neri, G.; Paludetti, G.; Fetoni, A.R.; Cianfrone, F.; Pomponi, M.G. Correlation between GJB2 mutations and audiological deficits: personal experience. *European archives of oto-rhino-laryngology : official journal of the European Federation of Oto-Rhino-Laryngological Societies* **2009**, *266*, 489-494, doi:10.1007/s00405-008-0775-9.

234. 233. Pollak, A.; Skorka, A.; Mueller-Malesinska, M.; Kostrzewa, G.; Kisiel, B.; Waligora, J.; Krajewski, P.; Oldak, M.; Korniszewski, L.; Skarzynski, H., et al. M34T and V37I mutations in GJB2 associated hearing impairment: evidence for pathogenicity and reduced penetrance. *American journal of medical genetics. Part A* **2007**, *143a*, 2534-2543, doi:10.1002/ajmg.a.31982.

235. 234. Popova, D.P.; Kaneva, R.; Varbanova, S.; Popov, T.M. Prevalence of GBJ2 mutations in patients with severe to profound congenital nonsyndromic sensorineural hearing loss in Bulgarian population. *European archives of oto-rhino-laryngology : official journal of the European Federation of Oto-Rhino-Laryngological Societies* **2012**, *269*, 1589-1592, doi:10.1007/s00405-011-1817-2.

236. 235. Prasad, S.; Cucci, R.A.; Green, G.E.; Smith, R.J. Genetic testing for hereditary hearing loss: connexin 26 (GJB2) allele variants and two novel deafness-causing mutations (R32C and 645-648delTAGA). *Human mutation* **2000**, *16*, 502-508, doi:10.1002/1098-1004(200012)16:6<502::aid-humu7>3.0.co;2-4.

237. 236. Primignani, P.; Castorina, P.; Sironi, F.; Curcio, C.; Ambrosetti, U.; Coviello, D.A. A novel dominant missense mutation--D179N--in the GJB2 gene (Connexin 26) associated with non-syndromic hearing loss. *Clinical genetics* **2003**, *63*, 516-521, doi:10.1034/j.1399-0004.2003.00079.x.

238. 237. Qing, J.; Zhou, Y.; Lai, R.; Hu, P.; Ding, Y.; Wu, W.; Xiao, Z.; Ho, P.T.; Liu, Y.; Liu, J., et al. Prevalence of mutations in GJB2, SLC26A4, and mtDNA in children with severe or profound sensorineural hearing loss in southwestern China. *Genet Test Mol Biomarkers* **2015**, *19*, 52-58, doi:10.1089/gtmb.2014.0241.

239. 238. Radulescu, L.; Martu, C.; Birkenhager, R.; Cozma, S.; Ungureanu, L.; Laszig, R. Prevalence of mutations located at the dfnb1 locus in a population of cochlear implanted children in eastern Romania. *International journal of pediatric otorhinolaryngology* **2012**, *76*, 90-94, doi:10.1016/j.ijporl.2011.10.007.

240. 239. Ramsebner, R.; Volker, R.; Lucas, T.; Hamader, G.; Weipoltshammer, K.; Baumgartner, W.D.; Wachtler, F.J.; Kirschhofer, K.; Frei, K. High incidence of GJB2 mutations during screening of newborns for hearing loss in Austria. *Ear and hearing* **2007**, *28*, 298-301, doi:10.1097/AUD.0b013e318047932d.

241. 240. Ravecca, F.; Berrettini, S.; Forli, F.; Marcaccini, M.; Casani, A.; Baldinotti, F.; Fogli, A.; Siciliano, G.; Simi, P. Cx26 gene mutations in idiopathic progressive hearing loss. *The Journal of otolaryngology* **2005**, *34*, 126-134, doi:10.2310/7070.2005.04017.

242. 241. Rezaei, H.; Vallian, B.S.; Movahedi, R. High frequency of 35delG mutation in GJB2 associated with autosomal recessive nonsyndromic hearing loss (ARNSHL) in the province of Isfahan-Iran. **2010**.
243. 242. Riahi, Z.; Chahed, H.; Jaafoura, H.; Zainine, R.; Messaoud, O.; Naili, M.; Nagara, M.; Hammami, H.; Laroussi, N.; Bouyacoub, Y., et al. A novel frameshift mutation (c.405delC) in the GJB2 gene associated with autosomal recessive hearing loss in two Tunisian families. *International journal of pediatric otorhinolaryngology* **2013**, *77*, 1485-1488, doi:10.1016/j.ijporl.2013.06.015.
244. 243. Riahi, Z.; Hammami, H.; Ouragini, H.; Messai, H.; Zainine, R.; Bouyacoub, Y.; Romdhane, L.; Essaid, D.; Kefi, R.; Rhimi, M., et al. Update of the spectrum of GJB2 gene mutations in Tunisian families with autosomal recessive nonsyndromic hearing loss. *Gene* **2013**, *525*, 1-4, doi:10.1016/j.gene.2013.04.078.
245. 244. Riahi, Z.; Zainine, R.; Mellouli, Y.; Hannachi, R.; Bouyacoub, Y.; Laroussi, N.; Beltaief, N.; Kefi, R.; Romdhane, L.; Bonnet, C., et al. Compound heterozygosity for dominant and recessive GJB2 mutations in a Tunisian family and association with successful cochlear implant outcome. *International journal of pediatric otorhinolaryngology* **2013**, *77*, 1481-1484, doi:10.1016/j.ijporl.2013.06.013.
246. 245. Rickard, S.; Kelsell, D.P.; Sirimana, T.; Rajput, K.; MacArdle, B.; Bitner-Glindzicz, M. Recurrent mutations in the deafness geneGJB2 (connexin 26) in British Asian families. *Journal of medical genetics* **2001**, *38*, 530-533.
247. 246. Rodriguez-Paris, J.; Pique, L.; Colen, T.; Roberson, J.; Gardner, P.; Schrijver, I. Genotyping with a 198 mutation arrayed primer extension array for hereditary hearing loss: assessment of its diagnostic value for medical practice. *PloS one* **2010**, *5*, e11804, doi:10.1371/journal.pone.0011804.
248. 247. Rodriguez-Paris, J.; Tamayo, M.L.; Gelvez, N.; Schrijver, I. Allele-specific impairment of GJB2 expression by GJB6 deletion del(GJB6-D13S1854). *PloS one* **2011**, *6*, e21665, doi:10.1371/journal.pone.0021665.
249. 248. Sadeghi, A.; Sanati, M.H.; Alasti, F.; Hashemzadeh Chaleshtori, M.; Ataei, M. Mutation analysis of connexin 26 gene and del (GJB6-D13S1830) in patients with hereditary deafness from two provinces in Iran. *Iranian Journal of Biotechnology* **2005**, *3*, 255-258.
250. 249. Sadeghi, A.; Sanati, M.H.; Alasti, F.; Hashemzadeh-Chaleshtori, M.; Mahmoudian, S.; Ataei, M. Contribution of GJB2 mutations and Four common DFNB loci in autosomal recessive non-syndromic hearing impairment in Markazi and Qom provinces of Iran. *Iranian Journal of Biotechnology* **2009**, *7*, 108-111.
251. 250. Salman, M.; Bashir, R.; Imtiaz, A.; Maqsood, A.; Mujtaba, G.; Iqbal, M.; Naz, S. Mutations of GJB2 encoding connexin 26 contribute to non-syndromic moderate and severe hearing loss in Pakistan. *European archives of oto-rhino-laryngology : official journal of the European Federation of Oto-Rhino-Laryngological Societies* **2015**, *272*, 2071-2075, doi:10.1007/s00405-015-3523-y.
252. 251. Salvago, P.; Martines, E.; La Mattina, E.; Mucia, M.; Sammarco, P.; Sireci, F.; Martines, F. Distribution and phenotype of GJB2 mutations in 102 Sicilian patients with congenital non syndromic sensorineural hearing loss. *International journal of audiology* **2014**, *53*, 558-563, doi:10.3109/14992027.2014.905717.
253. 252. Salvinelli, F.; Casale, M.; D'Ascanio, L.; Firrisi, L.; Greco, F.; Baldi, A. Hearing loss associated with 35delG mutation in Connexin-26 (GJB2) gene: audiogram analysis. *The Journal of laryngology and otology* **2004**, *118*, 8-11, doi:10.1258/002215504322731547.

254. 253. Samanich, J.; Lowes, C.; Burk, R.; Shanske, S.; Lu, J.; Shanske, A.; Morrow, B.E. Mutations in GJB2, GJB6, and mitochondrial DNA are rare in African American and Caribbean Hispanic individuals with hearing impairment. *American journal of medical genetics Part A* **2007**, *143*, 830-838.
255. 254. Santos, R.L.; Wajid, M.; Pham, T.L.; Hussan, J.; Ali, G.; Ahmad, W.; Leal, S.M. Low prevalence of Connexin 26 (GJB2) variants in Pakistani families with autosomal recessive non-syndromic hearing impairment. *Clinical genetics* **2005**, *67*, 61-68, doi:10.1111/j.1399-0004.2005.00379.x.
256. 255. Santos, R.L.P.; Aulchenko, Y.S.; Huygen, P.L.; van der Donk, K.P.; de Wijs, I.J.; Kemperman, M.H.; Admiraal, R.J.; Kremer, H.; Hoefsloot, L.H.; Cremers, C.W. Hearing impairment in Dutch patients with connexin 26 (GJB2) and connexin 30 (GJB6) mutations. *International journal of pediatric otorhinolaryngology* **2005**, *69*, 165-174.
257. 256. Schimmenti, L.A.; Martinez, A.; Telatar, M.; Lai, C.H.; Shapiro, N.; Fox, M.; Warman, B.; McCarra, M.; Crandall, B.; Sininger, Y., et al. Infant hearing loss and connexin testing in a diverse population. *Genet Med* **2008**, *10*, 517-524, doi:10.1097GIM.0b013e31817708fa
258. 10.1097/gim.0b013e31817708fa.
259. 257. Seeman, P.; Bendova, O.; Raskova, D.; Malikova, M.; Groh, D.; Kabelka, Z. Double heterozygosity with mutations involving both the GJB2 and GJB6 genes is a possible, but very rare, cause of congenital deafness in the Czech population. *Annals of human genetics* **2005**, *69*, 9-14, doi:10.1046/j.1529-8817.2003.00120.x.
260. 258. Seeman, P.; Malikova, M.; Rašková, D.; Bendova, O.; Groh, D.; Kubalkova, M.; Sakmaryova, I.; Seemanova, E.; Kabelka, Z. Spectrum and frequencies of mutations in the GJB2 (Cx26) gene among 156 Czech patients with pre‐lingual deafness. *Clinical genetics* **2004**, *66*, 152-157.
261. 259. Shafique, S.; Siddiqi, S.; Schraders, M.; Oostrik, J.; Ayub, H.; Bilal, A.; Ajmal, M.; Seco, C.Z.; Strom, T.M.; Mansoor, A., et al. Genetic spectrum of autosomal recessive non-syndromic hearing loss in Pakistani families. *PloS one* **2014**, *9*, e100146, doi:10.1371/journal.pone.0100146.
262. 260. Shahin, H.; Walsh, T.; Sobe, T.; Lynch, E.; King, M.C.; Avraham, K.B.; Kanaan, M. Genetics of congenital deafness in the Palestinian population: multiple connexin 26 alleles with shared origins in the Middle East. *Human genetics* **2002**, *110*, 284-289, doi:10.1007/s00439-001-0674-2.
263. 261. Shi, G.Z.; Gong, L.X.; Nie, W.Y.; Lin, Q.; Xiang, L.L.; Xu, X.H.; Qi, Y.S. [Mutations of GJB2 gene in infants with non-syndromic hearing impairment]. *Zhonghua yi xue za zhi* **2005**, *85*, 689-692.
264. 262. Shin, J.W.; Lee, S.C.; Lee, H.K.; Park, H.J. Genetic Screening of GJB2 and SLC26A4 in Korean Cochlear Implantees: Experience of Soree Ear Clinic. *Clin Exp Otorhinolaryngol* **2012**, *5 Suppl 1*, S10-13, doi:10.3342/ceo.2012.5.S1.S10.
265. 263. Siem, G.; Fagerheim, T.; Jonsrud, C.; Laurent, C.; Teig, E.; Harris, S.; Leren, T.P.; Fruh, A.; Heimdal, K. Causes of hearing impairment in the Norwegian paediatric cochlear implant program. *International journal of audiology* **2010**, *49*, 596-605, doi:10.3109/14992021003743269.
266. 264. Siemering, K.; Manji, S.S.M.; Hutchison, W.M.; Du Sart, D.; Phelan, D.; Dahl, H.H.M. Detection of mutations in genes associated with hearing loss using a microarray-based approach. *Journal of Molecular Diagnostics* **2006**, *8*, 483-489, doi:10.2353/jmoldx.2006.050147.
267. 265. Singh, P.K.; Sharma, S.; Ghosh, M.; Shastri, S.S.; Gupta, N.; Kabra, M. Spectrum of GJB2 gene variants in Indian children with non-syndromic hearing loss. *Indian J Med Res* **2018**, *147*, 615-618, doi:10.4103/ijmr.IJMR_76_16.

268. 266. Sinnathuray, A.R.; Toner, J.G.; Clarke-Lyttle, J.; Geddis, A.; Patterson, C.C.; Hughes, A.E. Connexin 26 (GJB2) gene-related deafness and speech intelligibility after cochlear implantation. *Otology & neurotology : official publication of the American Otological Society, American Neurotology Society [and] European Academy of Otology and Neurotology* **2004**, *25*, 935-942, doi:10.1097/00129492-200411000-00013.

269. 267. Sipeky, C.; Matyas, P.; Melegh, M.; Janicsek, I.; Szalai, R.; Szabo, I.; Varnai, R.; Tarlos, G.; Ganczer, A.; Melegh, B. Lower carrier rate of GJB2 W24X ancestral Indian mutation in Roma samples from Hungary: implication for public health intervention. *Mol Biol Rep* **2014**, *41*, 6105-6110, doi:10.1007/s11033-014-3488-8.

270. 268. Snoeckx, R.L.; Djelantik, B.; Van Laer, L.; Van de Heyning, P.; Van Camp, G. GJB2 (connexin 26) mutations are not a major cause of hearing loss in the Indonesian population. *American Journal of Medical Genetics Part A* **2005**, *135*, 126-129.

271. 269. Snoeckx, R.L.; Hassan, D.M.; Kamal, N.M.; Van Den Bogaert, K.; Van Camp, G. Mutation analysis of the GJB2 (connexin 26) gene in Egypt. *Human mutation* **2005**, *26*, 60-61, doi:10.1002/humu.9350.

272. 270. Svidnicki, M.C.; Silva-Costa, S.M.; Ramos, P.Z.; dos Santos, N.Z.; Martins, F.T.; Castilho, A.M.; Sartorato, E.L. Screening of genetic alterations related to non-syndromic hearing loss using MassARRAY iPLEX(R) technology. *BMC Med Genet* **2015**, *16*, 85, doi:10.1186/s12881-015-0232-8.

273. 271. Tamayo, M.; Olarte, M.; Gelvez, N.; Gómez, M.; Frías, J.; Bernal, J.; Florez, S.; Medina, D. Molecular studies in the GJB2 gene (Cx26) among a deaf population from Bogotá, Colombia: results of a screening program. *International journal of pediatric otorhinolaryngology* **2009**, *73*, 97-101.

274. 272. Taniguchi, M.; Matsuo, H.; Shimizu, S.; Nakayama, A.; Suzuki, K.; Hamajima, N.; Shinomiya, N.; Nishio, S.; Kosugi, S.; Usami, S., et al. Carrier frequency of the GJB2 mutations that cause hereditary hearing loss in the Japanese population. *Journal of human genetics* **2015**, *60*, 613-617, doi:10.1038/jhg.2015.82.

275. 273. Tarkan, O.; Sari, P.; Demirhan, O.; Kiroglu, M.; Tuncer, U.; Surmelioglu, O.; Ozdemir, S.; Yilmaz, M.B.; Kara, K. Connexin 26 and 30 mutations in paediatric patients with congenital, non-syndromic hearing loss treated with cochlear implantation in Mediterranean Turkey. *The Journal of laryngology and otology* **2013**, *127*, 33-37, doi:10.1017/s0022215112002587.

276. 274. Tekin, M.; Akar, N.; Cin, S.; Blanton, S.H.; Xia, X.J.; Liu, X.Z.; Nance, W.E.; Pandya, A. Connexin 26 (GJB2) mutations in the Turkish population: implications for the origin and high frequency of the 35delG mutation in Caucasians. *Human genetics* **2001**, *108*, 385-389, doi:10.1007/s004390100507.

277. 275. Tekin, M.; Bogoclu, G.; Arican, S.T.; Orman, M.N.; Tastan, H.; Elsobky, E.; Elsayed, S.; Akar, N. Evidence for single origins of 35delG and delE120 mutations in the GJB2 gene in Anatolia. *Clinical genetics* **2005**, *67*, 31-37, doi:10.1111/j.1399-0004.2004.00334.x.

278. 276. Tekin, M.; Xia, X.J.; Erdenetungalag, R.; Cengiz, F.B.; White, T.W.; Radnaabazar, J.; Dangaasuren, B.; Tastan, H.; Nance, W.E.; Pandya, A. GJB2 mutations in Mongolia: complex alleles, low frequency, and reduced fitness of the deaf. *Annals of human genetics* **2010**, *74*, 155-164, doi:10.1111/j.1469-1809.2010.00564.x.

279. 277. Tian, Y.S.; Chen, X.W.; Cao, K.L.; Chen, D.Y.; Zuo, J.; Fang, F.D. [Analysis of genetic mutation in patients with nonsyndromic hearing loss received cochlear implant]. *Zhonghua yi xue za zhi* **2007**, *87*, 1093-1096.

280. 278. Tian, Y.; Wang, Z.; Yang, N.; Hui, L.; Jiang, X. [Common deafness gene mutations of non-syndromic hearing loss in Liaoning]. *Lin chuang er bi yan hou tou jing wai ke za zhi = Journal of clinical otorhinolaryngology, head, and neck surgery* **2014**, *28*, 1244-1247.

281. 279. Tlili, A.; Al Mutery, A.; Kamal Eddine Ahmad Mohamed, W.; Mahfood, M.; Hadj Kacem, H. Prevalence of GJB2 Mutations in Affected Individuals from United Arab Emirates with Autosomal Recessive Nonsyndromic Hearing Loss. *Genet Test Mol Biomarkers* **2017**, *21*, 686-691, doi:10.1089/gtmb.2017.0130.

282. 280. Tsukada, K.; Nishio, S.; Usami, S.; Deafness Gene Study, C. A large cohort study of GJB2 mutations in Japanese hearing loss patients. *Clinical genetics* **2010**, *78*, 464-470, doi:10.1111/j.1399-0004.2010.01407.x.

283. 281. Uyguner, O.; Emiroglu, M.; Uzumcu, A.; Hafiz, G.; Ghanbari, A.; Baserer, N.; Yuksel-Apak, M.; Wollnik, B. Frequencies of gap- and tight-junction mutations in Turkish families with autosomal-recessive non-syndromic hearing loss. *Clinical genetics* **2003**, *64*, 65-69, doi:10.1034/j.1399-0004.2003.00101.x.

284. 282. Wang, W.; Cheng, H.B.; Yang, N.; Shi, Y.C.; Liu, J.Z.; Li, Q.; Yang, S.M.; Shen, L.Y.; Liu, M.J.; Chen, Y., et al. [Analysis of GJB2 gene and mitochondrial DNA A1555G mutations in 16 families with non-syndromic hearing loss]. *Zhonghua yi xue yi chuan xue za zhi = Zhonghua yixue yichuanxue zazhi = Chinese journal of medical genetics* **2012**, *29*, 388-392, doi:10.3760/cma.j.issn.1003-9406.2012.04.003.

285. 283. Wang, Y.C.; Kung, C.Y.; Su, M.C.; Su, C.C.; Hsu, H.M.; Tsai, C.C.; Lin, C.C.; Li, S.Y. Mutations of Cx26 gene (GJB2) for prelingual deafness in Taiwan. *European journal of human genetics : EJHG* **2002**, *10*, 495-498, doi:10.1038/sj.ejhg.5200838.

286. 284. Wang, Y.L.; Zhu, Y.M.; Liu, X.W.; Xu, B.C.; Guo, Y.F.; Wang, Q.J. [Analysis of the hereditary etiology of 336 patients with non-syndromic sensorineural hearing loss from Ningxia Hui Autonomous Region of China]. *Zhonghua er bi yan hou tou jing wai ke za zhi = Chinese journal of otorhinolaryngology head and neck surgery* **2012**, *47*, 760-763.

287. 285. Wei, Q.; Wang, S.; Yao, J.; Lu, Y.; Chen, Z.; Xing, G.; Cao, X. Genetic mutations of GJB2 and mitochondrial 12S rRNA in nonsyndromic hearing loss in Jiangsu Province of China. *J Transl Med* **2013**, *11*, 163, doi:10.1186/1479-5876-11-163.

288. 286. Wilch, E.; Azaiez, H.; Fisher, R.A.; Elfenbein, J.; Murgia, A.; Birkenhäger, R.; Bolz, H.; Da Silva‐Costa, S.; Del Castillo, I.; Haaf, T. A novel DFNB1 deletion allele supports the existence of a distant cis‐regulatory region that controls GJB2 and GJB6 expression. *Clinical genetics* **2010**, *78*, 267-274.

289. 287. Wilcox, S.A.; Saunders, K.; Osborn, A.H.; Arnold, A.; Wunderlich, J.; Kelly, T.; Collins, V.; Wilcox, L.J.; McKinlay Gardner, R.J.; Kamarinos, M., et al. High frequency hearing loss correlated with mutations in the GJB2 gene. *Human genetics* **2000**, *106*, 399-405, doi:10.1007/s004390000273.

290. 288. Wu, B.L.; Lindeman, N.; Lip, V.; Adams, A.; Amato, R.S.; Cox, G.; Irons, M.; Kenna, M.; Korf, B.; Raisen, J., et al. Effectiveness of sequencing connexin 26 (GJB2) in cases of familial or sporadic childhood deafness referred for molecular diagnostic testing. *Genet Med* **2002**, *4*, 279-288, doi:10.1097/00125817-200207000-00006.

291. 289. Wu, C.C.; Chen, P.J.; Chiu, Y.H.; Lu, Y.C.; Wu, M.C.; Hsu, C.J. Prospective mutation screening of three common deafness genes in a large Taiwanese cohort with idiopathic bilateral sensorineural hearing impairment reveals a difference in the results between families from hospitals and those from rehabilitation facilities. *Audiology and Neuro-Otology* **2008**, *13*, 172-181, doi:10.1159/000112425.

292. 290. Wu, C.C.; Hung, C.C.; Lin, S.Y.; Hsieh, W.S.; Tsao, P.N.; Lee, C.N.; Su, Y.N.; Hsu, C.J. Newborn Genetic Screening for Hearing Impairment: A Preliminary Study at a Tertiary Center. *PloS one* **2011**, *6*, e22314, doi:ARTN e22314

293. 10.1371/journal.pone.0022314.

294. 291. Xia, H.; Xu, H.; Deng, X.; Yuan, L.; Xiong, W.; Yang, Z.; Deng, H. Compound heterozygous GJB2 mutations associated to a consanguineous Han family with autosomal recessive non-syndromic hearing loss. *Acta oto-laryngologica* **2016**, *136*, 782-785, doi:10.3109/00016489.2016.1157727.

295. 292. Xin, F.; Yuan, Y.; Deng, X.; Han, M.; Wang, G.; Zhao, J.; Gao, X.; Liu, J.; Yu, F.; Han, D., et al. Genetic mutations in nonsyndromic deafness patients of Chinese minority and Han ethnicities in Yunnan, China. *J Transl Med* **2013**, *11*, 312, doi:10.1186/1479-5876-11-312.

296. 293. Yang, J.J.; Wang, W.H.; Lin, Y.C.; Weng, H.H.; Yang, J.T.; Hwang, C.F.; Wu, C.M.; Li, S.Y. Prospective variants screening of connexin genes in children with hearing impairment: genotype/phenotype correlation. *Human genetics* **2010**, *128*, 303-313, doi:10.1007/s00439-010-0856-x.

297. 294. Yoong, S.Y.; Mavrogiannis, L.A.; Wright, J.; Fairley, L.; Bennett, C.P.; Charlton, R.S.; Spencer, N. Low prevalence of DFNB1 (connexin 26) mutations in British Pakistani children with non-syndromic sensorineural hearing loss. *Arch Dis Child* **2011**, *96*, 798-803, doi:10.1136/adc.2010.209262.

298. 295. Yuan, Y.; Yu, F.; Wang, G.; Huang, S.; Yu, R.; Zhang, X.; Huang, D.; Han, D.; Dai, P. Prevalence of the GJB2 IVS1+1G >A mutation in Chinese hearing loss patients with monoallelic pathogenic mutation in the coding region of GJB2. *J Transl Med* **2010**, *8*, 127, doi:10.1186/1479-5876-8-127.

299. 296. Yuan, Y.; Zhang, X.; Huang, S.; Zuo, L.; Zhang, G.; Song, Y.; Wang, G.; Wang, H.; Huang, D.; Han, D., et al. Common molecular etiologies are rare in nonsyndromic Tibetan Chinese patients with hearing impairment. *PloS one* **2012**, *7*, e30720, doi:10.1371/journal.pone.0030720.

300. 297. Zainal, S.A.; Md Daud, M.K.; Abd Rahman, N.; Zainuddin, Z.; Alwi, Z. Mutation detection in GJB2 gene among Malays with non-syndromic hearing loss. *International journal of pediatric otorhinolaryngology* **2012**, *76*, 1175-1179, doi:10.1016/j.ijporl.2012.04.027.

301. 298. Zeinali, S.; Davoudi-Dehaghani, E.; Azadmehr, S.; DabbaghBagheri, S.; Bagherian, H.; Jamali, M.; Zafarghandimotlagh, F.; Masoodifard, M.; BandehiSarhaddi, A.; Rejali, L., et al. GJB2 c.-23+1G>A mutation is second most common mutation among Iranian individuals with autosomal recessive hearing loss. *European archives of oto-rhino-laryngology : official journal of the European Federation of Oto-Rhino-Laryngological Societies* **2015**, *272*, 2255-2259, doi:10.1007/s00405-014-3171-7.

302. 299. Zheng, J.; Ying, Z.; Cai, Z.; Sun, D.; He, Z.; Gao, Y.; Zhang, T.; Zhu, Y.; Chen, Y.; Guan, M.X. GJB2 Mutation Spectrum and Genotype-Phenotype Correlation in 1067 Han Chinese Subjects with Non-Syndromic Hearing Loss. *PloS one* **2015**, *10*, e0128691, doi:10.1371/journal.pone.0128691.

303. 300. Zhou, Y.; Li, C.; Li, M.; Zhao, Z.; Tian, S.; Xia, H.; Liu, P.; Han, Y.; Ren, R.; Chen, J., et al. Mutation analysis of common deafness genes among 1,201 patients with non-syndromic hearing loss in Shanxi Province. *Mol Genet Genomic Med* **2019**, *7*, e537, doi:10.1002/mgg3.537.

304. 301. Abdi Rad, I.; Bagheri, M.; Farhoudi, F. The frequency of M34T, 167delT, 235delC and 35delG mutations in GJB2 gene in autosomal recessive non-syndromic hearing loss patients in West Azarbaijan. *Journal of Zanjan University of Medical Sciences and Health Services* **2011**, *19*.

305. 302. Adadey, S.M.; Tingang Wonkam, E.; Twumasi Aboagye, E.; Quansah, D.; Asante-Poku, A.; Quaye, O.; Amedofu, G.K.; Awandare, G.A.; Wonkam, A. Enhancing Genetic Medicine: Rapid and Cost-Effective Molecular Diagnosis for a GJB2 Founder Mutation for Hearing Impairment in Ghana. *Genes* **2020**, *11*, 132.

306. 303. Adhikary, B.; Ghosh, S.; Paul, S.; Bankura, B.; Pattanayak, A.K.; Biswas, S.; Maity, B.; Das, M. Spectrum and frequency of GJB2, GJB6 and SLC26A4 gene mutations among nonsyndromic hearing loss patients in eastern part of India. *Gene* **2015**, *573*, 239-245, doi:10.1016/j.gene.2015.07.050.

307. 304. Ammar-Khodja, F.; Makrelouf, M.; Malek, R.; Ibrahim, H.; Zenati, A. Frequency of the 35delG allele causing non-syndromic recessive deafness in the Algerian patients. *Genetic Counseling* **2007**, *18*, 383-391.

308. 305. Angeli, S.I. Phenotype/genotype correlations in a DFNB1 cohort with ethnical diversity. *The Laryngoscope* **2008**, *118*, 2014-2023, doi:10.1097/MLG.0b013e31817fb7ad.

309. 306. Arik Biyikli, T. Prevalence of the IVS1(+1)G→A and 35delG mutations in the GJB2 gene of Turkish patients with nonsyndromic hearing loss. *Turkish Journal of Biology* **2012**, *36*, 1-6, doi:10.3906/biy-1012-174.

310. 307. Asma, A.; Ashwaq, A.; Norzana, A.G.; Maizaton Atmadini, A.; Ruszymah, B.H.I.; Saim, L.; Farah Wahida, I. The association between GJB2 mutation and GJB6 gene in non syndromic hearing loss school children. *Medical Journal of Malaysia* **2011**, *66*, 124-128.

311. 308. Azaiez, H.; Chamberlin, G.P.; Fischer, S.M.; Welp, C.L.; Prasad, S.D.; Taggart, R.T.; del Castillo, I.; Van Camp, G.; Smith, R.J. GJB2: the spectrum of deafness-causing allele variants and their phenotype. *Human mutation* **2004**, *24*, 305-311, doi:10.1002/humu.20084.

312. 309. Baris, I.; Koksal, V. Multiplex detection of common mutations in the Connexin-26 gene. *Genet Test* **2003**, *7*, 63-65, doi:10.1089/109065703321560967.

313. 310. Baris, I.; Koksal, V.; Etlik, O. A combined allele-specific PCR and RFLP assay to detect the 35delG mutation in the Connexin 26 gene. *Genet Test* **2004**, *8*, 384-386, doi:10.1089/gte.2004.8.384.

314. 311. Bartsch, O.; Vatter, A.; Zechner, U.; Kohlschmidt, N.; Wetzig, C.; Baumgart, A.; Nospes, S.; Haaf, T.; Keilmann, A. GJB2 mutations and genotype-phenotype correlation in 335 patients from germany with nonsyndromic sensorineural hearing loss: evidence for additional recessive mutations not detected by current methods. *Audiology & neuro-otology* **2010**, *15*, 375-382, doi:10.1159/000297216.

315. 312. Battellino, S.; Rudolf, G.; Zargi, M.; Podkrajsek, K.T.; Peterlin, B. Connexin 26 (GJB2) and connexin 30 del(GJB6-D13S1830) mutations in Slovenians with prelingual non-syndromic deafness. *Journal of International Advanced Otology* **2011**, *7*, 372-378.

316. 313. Baysal, E.; Bayazit, Y.A.; Ceylaner, S.; Alatas, N.; Donmez, B.; Ceylaner, G.; San, I.; Korkmaz, B.; Yilmaz, A.; Menevse, A., et al. GJB2 and mitochondrial A1555G gene mutations in nonsyndromic profound hearing loss and carrier frequencies in healthy individuals. *J Genet* **2008**, *87*, 53-57, doi:10.1007/s12041-008-0007-5.

317. 314. Berto, A.; Pellati, D.; Castiglione, A.; Busi, M.; Trevisi, P.; Gualandi, F.; Ferlini, A.; Martini, A. Audiological profiles and gjb2, gjb6 mutations: A retrospective study on genetic and clinical data from 2003 to 2008. *Audiological Medicine* **2009**, *7*, 93-105, doi:10.1080/16513860902900136.

318. 315. Bliznets, E.A.; Galkina, V.A.; Matiushchenko, G.N.; Kisina, A.G.; Markova, T.G.; Poliakov, A.V. [Changes in the connexin 26 (GJB2) gene in Russian patients with hearing disorders: results of long-term molecular diagnostics of hereditary nonsyndromic deafness]. *Genetika* **2012**, *48*, 112-124.

319. 316. Bouwer, S.; Angelicheva, D.; Chandler, D.; Seeman, P.; Tournev, I.; Kalaydjieva, L. Carrier rates of the ancestral Indian W24X mutation in GJB2 in the general Gypsy population and individual subisolates. *Genet Test* **2007**, *11*, 455-458, doi:10.1089/gte.2007.0048.

320. 317. Chaleshtori, M.H.; Farhud, D.D.; Crosby, A.H.; Farrokhi, E.; Jafari, H.P.; Samani, G.; Chaleshtori, K.S.; Kasiri, M.; Shahrani, M.; Mobini, G.R., et al. Molecular pathology of 6 novel GJB2 allelic variants detected in familial and sporadic Iranian non syndromic hearing loss cases. *Iranian Journal of Public Health* **2008**, *37*, 9-18.

321. 318. Chaleshtori, M.H.; Zohour, M.M.; Rad, L.H.; Pour-Jafari, H.; Farhud, D.D.; Dolati, M.; Chaleshtori, K.S.; Sasanfar, R.; Hosseinipour, A.; Andonian, L., et al. Autosomal recessive and sporadic non syndromic hearing loss and the incidence of Cx26 mutations in a province of Iran. *Iranian Journal of Public Health* **2006**, *35*, 88-91.

322. 319. Cheng, X.; Li, L.; Brashears, S.; Morlet, T.; Ng, S.S.; Berlin, C.; Hood, L.; Keats, B. Connexin 26 variants and auditory neuropathy/dys-synchrony among children in schools for the deaf. *American journal of medical genetics. Part A* **2005**, *139*, 13-18, doi:10.1002/ajmg.a.30929.

323. 320. Choi, B.Y.; Park, G.; Gim, J.; Kim, A.R.; Kim, B.J.; Kim, H.S.; Park, J.H.; Park, T.; Oh, S.H.; Han, K.H., et al. Diagnostic application of targeted resequencing for familial nonsyndromic hearing loss. *PloS one* **2013**, *8*, e68692, doi:10.1371/journal.pone.0068692.

324. 321. Cordeiro-Silva Mde, F.; Barbosa, A.; Santiago, M.; Provetti, M.; Dettogni, R.S.; Tovar, T.T.; Rabbi-Bortolini, E.; Louro, I.D. Mutation analysis of GJB2 and GJB6 genes in Southeastern Brazilians with hereditary nonsyndromic deafness. *Mol Biol Rep* **2011**, *38*, 1309-1313, doi:10.1007/s11033-010-0231-y.

325. 322. Cucci, R.A.; Prasad, S.; Kelley, P.M.; Green, G.E.; Storm, K.; Willocx, S.; Cohn, E.S.; Van Camp, G.; Smith, R.J. The M34T allele variant of connexin 26. *Genet Test* **2000**, *4*, 335-344, doi:10.1089/109065700750065063.

326. 323. da Silva-Costa, S.M.; Coeli, F.B.; Lincoln-de-Carvalho, C.R.; Marques-de-Faria, A.P.; Kurc, M.; Pereira, T.; Pomilio, M.C.; Sartorato, E.L. Screening for the GJB2 c.-3170 G>A (IVS 1+1 G>A) mutation in Brazilian deaf individuals using multiplex ligation-dependent probe amplification. *Genet Test Mol Biomarkers* **2009**, *13*, 701-704, doi:10.1089/gtmb.2009.0025.

327. 324. da Silva-Costa, S.M.; Martins, F.T.; Pereira, T.; Pomilio, M.C.; Marques-de-Faria, A.P.; Sartorato, E.L. Searching for digenic inheritance in deaf Brazilian individuals using the multiplex ligation-dependent probe amplification technique. *Genet Test Mol Biomarkers* **2011**, *15*, 849-853, doi:10.1089/gtmb.2011.0034.

328. 325. Daneshi, A.; Hassanzadeh, S.; Emamdjomeh, H.; Mohammadi, S.H.; Arzhangi, S.; Farhadi, M.; Najmabadi, H. Prevalence of GJB2-associated deafness and outcomes of cochlear implantation in Iran. *The Journal of laryngology and otology* **2011**, *125*, 455-459, doi:10.1017/S0022215110002999.

329. 326. Davarnia, B.; Babanejad, M.; Fattahi, Z.; Nikzat, N.; Bazazzadegan, N.; Pirzade, A.; Farajollahi, R.; Nishimura, C.; Jalalvand, K.; Arzhangi, S., et al. Spectrum of GJB2 (Cx26) gene mutations in Iranian Azeri patients with nonsyndromic autosomal recessive hearing loss. *International journal of pediatric otorhinolaryngology* **2012**, *76*, 268-271, doi:10.1016/j.ijporl.2011.11.019.

330. 327. Deng, Y.; Sang, S.; Wen, J.; Liu, Y.; Ling, J.; Chen, H.; Cai, X.; Mei, L.; Chen, X.; Li, M., et al. Reproductive guidance through prenatal diagnosis and genetic counseling for recessive hereditary hearing loss in high-risk families. *International journal of pediatric otorhinolaryngology* **2018**, *115*, 114-119, doi:10.1016/j.ijporl.2018.08.026.

331. 328. Dent, K.M.; Kenneson, A.; Palumbos, J.C.; Maxwell, S.; Eichwald, J.; White, K.; Mao, R.; Bale, J.F., Jr.; Carey, J.C. Methodology of a multistate study of congenital hearing loss: preliminary data from Utah newborn screening. *American journal of medical genetics. Part C, Seminars in medical genetics* **2004**, *125C*, 28-34, doi:10.1002/ajmg.c.30002.

332. 329. Dietz, A.; Lopponen, T.; Valtonen, H.; Hyvarinen, A.; Lopponen, H. Prevalence and etiology of congenital or early acquired hearing impairment in Eastern Finland. *International journal of pediatric otorhinolaryngology* **2009**, *73*, 1353-1357, doi:10.1016/j.ijporl.2009.06.009.

333. 330. Dória, M.; Fernandes, S.; Moura, C.P. Study of Met34Thr variant in nonsyndromic hearing loss in four Portuguese families. *Porto Biomedical Journal* **2016**, *1*, 32-35, doi:10.1016/j.pbj.2015.07.001.

334. 331. Doria, M.; Neto, A.P.; Santos, A.C.; Barros, H.; Fernandes, S.; Moura, C.P. Prevalence of 35delG and Met34Thr GJB2 variants in Portuguese samples. *International journal of pediatric otorhinolaryngology* **2015**, *79*, 2187-2190, doi:10.1016/j.ijporl.2015.09.046.

335. 332. Du, W.; Cheng, J.; Ding, H.; Jiang, Z.; Guo, Y.; Yuan, H. A rapid method for simultaneous multi-gene mutation screening in children with nonsyndromic hearing loss. *Genomics* **2014**, *104*, 264-270, doi:10.1016/j.ygeno.2014.07.009.

336. 333. Du, W.; Zhu, Y.M.; Guo, Y.F.; Wang, Q.J.; Liu, X.W. Prevalence of GJB2 mutations in the Silk Road region of China and a report of three novel variants. *Acta oto-laryngologica* **2014**, *134*, 373-381, doi:10.3109/00016489.2013.849817.

337. 334. Duan, S.H.; Ma, J.L.; Yang, X.L.; Guo, Y.F. Simultaneous multigene mutation screening using SNPscan in patients from ethnic minorities with nonsyndromic hearingimpairment in Northwest China. *Molecular medicine reports* **2017**, *16*, 6722-6728, doi:10.3892/mmr.2017.7431.

338. 335. Duan, S.H.; Zhu, Y.M.; Wang, Y.L.; Guo, Y.F. Common molecular etiology of nonsyndromic hearing loss in 484 patients of 3 ethnicities in northwest China. *Acta oto-laryngologica* **2015**, *135*, 586-591, doi:10.3109/00016489.2015.1006334.

339. 336. Düzcan, F.; Wollnik, B.; Tepeli, E.; Ardiç, F.N.; Uyguner, O.; Bagci, H. Family history, clinical features, and molecular characterization of a patient with autosomal recessive non-syndromic hearing loss. *Kulak burun bogaz ihtisas dergisi : KBB = Journal of ear, nose, and throat* **2003**, *11*, 85-88.

340. 337. El Barbary, N.; El Belbesy, M.; Asal, S.; Kholeif, S. Detection of 35delG, 167delT mutations in the connexin 26 gene among Egyptian patients with nonsyndromic sensorineural hearing loss. *The Egyptian Journal of Otolaryngology* **2015**, *31*, 42, doi:10.4103/1012-5574.152707.

341. 338. Erdenechuluun, J.; Lin, Y.H.; Ganbat, K.; Bataakhuu, D.; Makhbal, Z.; Tsai, C.Y.; Lin, Y.H.; Chan, Y.H.; Hsu, C.J.; Hsu, W.C., et al. Unique spectra of deafness-associated mutations in Mongolians provide insights into the genetic relationships among Eurasian populations. *PloS one* **2018**, *13*, e0209797, doi:10.1371/journal.pone.0209797.

342. 339. Eyerci, N.; Altas, E.; Pirim, I. GJB2 mutations in Turkish patients with nonsyndromic hearing loss. *Meta Gene* **2016**, *10*, 56-60, doi:10.1016/j.mgene.2016.10.006.

343. 340. Falah, M.; Houshmand, M.; Akbaroghli, S.; Mahmodian, S.; Ghavami, Y.; Farhadi, M. Profile of Iranian GJB2 Mutations in Young Population with Novel Mutation. *Iranian Journal of Basic Medical Sciences* **2011**, *14*, 213-218.

344. 341. Felix, F.; Ribeiro, M.G.; Tomita, S.; Zalis, M.G. Frequency of GJB2 mutations in patients with nonsyndromic hearing loss from an ethnically characterized Brazilian population. *Brazilian journal of otorhinolaryngology* **2019**, *85*, 92-98, doi:10.1016/j.bjorl.2017.10.013.

345. 342. Gallo-Terán, J.; Morales-Angulo, C.; Rodríguez-Ballesteros, M.; Moreno-Pelayo, M.A.; del Castillo, I.; Moreno, F. Prevalencia de las mutaciones 35delG en el gen GJB2, del (GJB6-D13S1830) en el gen GJB6, Q829X en el gen OTOF y A1555G en el gen del ARNr 12S mitocondrial en sujetos con hipoacusia neurosensorial no sindrómica de inicio congénito o en la infancia. *Acta otorrinolaringologica espanola* **2005**, *56*, 463-468, doi:10.1016/s0001-6519(05)78649-0.

346. 343. Gandia, M.; Del Castillo, F.J.; Rodriguez-Alvarez, F.J.; Garrido, G.; Villamar, M.; Calderon, M.; Moreno-Pelayo, M.A.; Moreno, F.; del Castillo, I. A novel splice-site mutation in the GJB2 gene causing mild postlingual hearing impairment. *PloS one* **2013**, *8*, e73566, doi:10.1371/journal.pone.0073566.

347. 344. Griffith, A.J.; Chowdhry, A.A.; Kurima, K.; Hood, L.J.; Keats, B.; Berlin, C.I.; Morell, R.J.; Friedman, T.B. Autosomal recessive nonsyndromic neurosensory deafness at DFNB1 not associated with the compound-heterozygous GJB2 (connexin 26) genotype M34T/167delT. *American journal of human genetics* **2000**, *67*, 745-749, doi:10.1086/303045.

348. 345. Han, J.J.; Nguyen, P.D.; Oh, D.Y.; Han, J.H.; Kim, A.R.; Kim, M.Y.; Park, H.R.; Tran, L.H.; Dung, N.H.; Koo, J.W., et al. Elucidation of the unique mutation spectrum of severe hearing loss in a Vietnamese pediatric population. *Scientific reports* **2019**, *9*, 1604, doi:10.1038/s41598-018-38245-4.

349. 346. Han, R.; Li, L.; Duan, L.; Xia, Y.; Kuyaxi, P.; Zhao, J.; Zhao, Q.; Zhang, H.; Chen, Y. Efficiency of microarray and SNPscan for the detection of hearing loss gene in 71 cases with nonsyndromic hearing loss. *Medicine (Baltimore)* **2017**, *96*, e7149, doi:10.1097/MD.0000000000007149.

350. 347. Han, S.H.; Park, H.J.; Kang, E.J.; Ryu, J.S.; Lee, A.; Yang, Y.H.; Lee, K.R. Carrier frequency of GJB2 (connexin-26) mutations causing inherited deafness in the Korean population. *Journal of human genetics* **2008**, *53*, 1022-1028, doi:10.1007/s10038-008-0342-7.

351. 348. Harris, K.C.; Erbe, C.B.; Firszt, J.B.; Flanary, V.A.; Wackym, P.A. A novel connexin 26 compound heterozygous mutation results in deafness. *The Laryngoscope* **2002**, *112*, 1159-1162, doi:10.1097/00005537-200207000-00003.

352. 349. Hashemzadeh Chaleshtori, M.; Farhud, D.D.; Crosby, A.H.; Farrokhi, E.; Pour Jafari, H.; Ghatreh Samani, K.; Safa Chaleshtori, K.; Kasiri, M.; Shahrani, M.; Mobini, G.R., et al. Molecular pathology of 6 novel GJB2 allelic variants detected in familial and sporadic Iranian non syndromic hearing loss cases. *Iranian Journal of Public Health* **2008**, *37*, 9-18.

353. 350. Hashemzadeh Chaleshtori, M.; Montazer Zohour, M.; Hoghooghi Rad, L.; Pour-Jafari, H.; Farhud, D.D.; Dolati, M.; Safa Chaleshtori, K.; Sasanfar, R.; Hosseinipour, A.; Andonian, L., et al. Autosomal recessive and sporadic non syndromic hearing loss and the incidence of Cx26 mutations in a province of Iran. *Iranian Journal of Public Health* **2006**, *35*, 88-91.

354. 351. Hellstrom, S. Wan Du et al. - Prevalence of GJB2 mutations in the Silk Road region of China and a report of three novel variants. Acta Oto-Laryngol 2014; 134: 373-381. *Acta oto-laryngologica* **2018**, *138*, 329-338, doi:10.1080/00016489.2018.1438162.

355. 352. Hernandez-Juarez, A.A.; Lugo-Trampe Jde, J.; Campos-Acevedo, L.D.; Lugo-Trampe, A.; Trevino-Gonzalez, J.L.; de-la-Cruz-Avila, I.; Martinez-de-Villarreal, L.E. GJB2 and GJB6 mutations are an infrequent cause of autosomal-recessive nonsyndromic hearing loss in residents of Mexico. *International journal of pediatric otorhinolaryngology* **2014**, *78*, 2107-2112, doi:10.1016/j.ijporl.2014.09.016.

356. 353. Huang, S.; Wang, G.; Xu, Y.; Yuan, Y.; Han, D.; Dai, P. Identification of a p.R143Q dominant mutation in the gap junction beta-2 gene in three Chinese patients with different hearing phenotypes. *Acta oto-laryngologica* **2013**, *133*, 55-58, doi:10.3109/00016489.2012.715373.

357. 354. Iliades, T.; Eleftheriades, N.; Iliadou, V.; Pampanos, A.; Voyiatzis, N.; Economides, J.; Leotsakos, P.; Neou, P.; Tsakanikos, M.; Antoniadi, T., et al. Prelingual nonsyndromic hearing loss in Greece. Molecular and clinical findings. *ORL; journal for oto-rhino-laryngology and its related specialties* **2002**, *64*, 321-323, doi:10.1159/000066088.

358. 355. Jakubikova, J.; Kabatova, Z.; Pavlovcinova, G.; Profant, M. Newborn hearing screening and strategy for early detection of hearing loss in infants. *International journal of pediatric otorhinolaryngology* **2009**, *73*, 607-612, doi:10.1016/j.ijporl.2008.12.006.

359. 356. Ji, Y.B.; Han, D.Y.; Lan, L.; Wang, D.Y.; Zong, L.; Zhao, F.F.; Liu, Q.; Benedict-Alderfer, C.; Zheng, Q.Y.; Wang, Q.J. Molecular epidemiological analysis of mitochondrial DNA12SrRNA A1555G, GJB2, and SLC26A4 mutations in sporadic outpatients with nonsyndromic sensorineural hearing loss in China. *Acta oto-laryngologica* **2011**, *131*, 124-129, doi:10.3109/00016489.2010.483479.

360. 357. Kasakura-Kimura, N.; Masuda, M.; Mutai, H.; Masuda, S.; Morimoto, N.; Ogahara, N.; Misawa, H.; Sakamoto, H.; Saito, K.; Matsunaga, T. WFS1 and GJB2 mutations in patients with bilateral low-frequency sensorineural hearing loss. *The Laryngoscope* **2017**, *127*, E324-E329, doi:10.1002/lary.26528.

361. 358. Kelley, P.M.; Harris, D.J.; Comer, B.C.; Askew, J.W.; Fowler, T.; Smith, S.D.; Kimberling, W.J. Novel mutations in the connexin 26 gene (GJB2) that cause autosomal recessive (DFNB1) hearing loss. *American journal of human genetics* **1998**, *62*, 792-799, doi:10.1086/301807.

362. 359. Khidiyatova, I.M.; Dzhemileva, L.U.; Khabibulin, R.M.; Khusnutdinova, E.K. Frequency of the 35delG mutation of the connexin 26 gene (GJB2) in patients with non-syndromic recessive deafness from Bashkortostan and in ethnic groups of the Volga-Ural region. *Molecular Biology* **2002**, *36*, 338-341, doi:Unsp Udc 575.174:599.9

363. Doi 10.1023/A:1016047010528.

364. 360. Kim, S.Y.; Lee, B.Y.; Lim, J.H.; Ryu, H.M.; Yang, J.H.; Chung, J.H.; Han, H.W.; Park, S.Y. Determination of the carrier frequencies of selected GJB2 mutations in the Korean population. *International journal of audiology* **2011**, *50*, 694-698, doi:10.3109/14992027.2011.563247.

365. 361. King, P.J.; Ouyang, X.; Du, L.; Yan, D.; Angeli, S.I.; Liu, X.Z. Etiologic diagnosis of nonsyndromic genetic hearing loss in adult vs pediatric populations. *Otolaryngology--head and neck surgery : official journal of American Academy of Otolaryngology-Head and Neck Surgery* **2012**, *147*, 932-936, doi:10.1177/0194599812453553.

366. 362. Kucuk Kurtulgan, H.; Altuntas, E.E.; Yildirim, M.E.; Ozdemir, O.; Bagci, B.; Sezgin, I. The Analysis of GJB2, GJB3, and GJB6 Gene Mutations in Patients with Hereditary Non-Syndromic Hearing Loss Living in Sivas. *J Int Adv Otol* **2019**, *15*, 373-378, doi:10.5152/iao.2019.5401.

367. 363. Kudo, T.; Oshima, T.; Kure, S.; Matsubara, Y.; Ikeda, K. Mutation detection of GJB2 using IsoCode and real-time quantitative polymerase chain reaction with SYBR green I dye for newborn hearing screening. *The Laryngoscope* **2004**, *114*, 1299-1304, doi:10.1097/00005537-200407000-00030.

368. 364. Lan, X.; Sun, S.; Lan, X.; Niu, L.; Zhang, C.; Chen, X.; Xia, N. Case report: Novel GJB2 variant c.113T>C associated with autosomal recessive non-syndromic hearing loss (ARNSHL) in a Han family. *Medicine (Baltimore)* **2019**, *98*, e18253, doi:10.1097/MD.0000000000018253.

369. 365. Lazar, C.; Popp, R.; Al-Khzouz, C.; Mihut, G.; Grigorescu-Sido, P. GJB2 and GJB6 genes mutations in children with non-syndromic hearing loss. *Revista Romana De Medicina De Laborator* **2017**, *25*, 37-46, doi:10.1515/rrlm-2017-0004.

370. 366. Lazar, C.; Popp, R.; Trifa, A.; Mocanu, C.; Mihut, G.; Al-Khzouz, C.; Tomescu, E.; Figan, I.; Grigorescu-Sido, P. Prevalence of the c.35delG and p.W24X mutations in the GJB2 gene in patients with nonsyndromic hearing loss from North-West Romania. *International journal of pediatric otorhinolaryngology* **2010**, *74*, 351-355, doi:10.1016/j.ijporl.2009.12.015.

371. 367. Li, H.; Wang, B.; Liu, D.; Wang, T.; Li, Q.; Wang, W.; Li, H. SNPscan as a high-performance screening tool for mutation hotspots of hearing loss-associated genes. *Genomics* **2015**, *106*, 83-87, doi:10.1016/j.ygeno.2015.05.004.

372. 368. Likar, T.; Hasanhodzic, M.; Teran, N.; Maver, A.; Peterlin, B.; Writzl, K. Diagnostic outcomes of exome sequencing in patients with syndromic or non-syndromic hearing loss. *PloS one* **2018**, *13*, e0188578, doi:10.1371/journal.pone.0188578.

373. 369. Lin, Y.; Yu, F.; Jiao, Y.; Zhou, F. Variations in the Mutational Spectrum in Nonsyndromic Hearing Impairment: A study of the Special Schools for the Deaf in Southern China. *J Int Adv Otol* **2019**, *15*, 247-252, doi:10.5152/iao.2019.6512.

374. 370. Lipan, M.; Ouyang, X.; Yan, D.; Angeli, S.; Du, L.L.; Liu, X.Z. Clinical comparison of hearing-impaired patients with DFNB1 against heterozygote carriers of connexin 26 mutations. *The Laryngoscope* **2011**, *121*, 811-814, doi:10.1002/lary.21422.

375. 371. Liu, Y.; Hu, C.; Liu, C.; Liu, D.; Mei, L.; He, C.; Jiang, L.; Wu, H.; Chen, H.; Feng, Y. A rapid improved multiplex ligation detection reaction method for the identification of gene mutations in hereditary hearing loss. *PloS one* **2019**, *14*, e0215212, doi:10.1371/journal.pone.0215212.

376. 372. Liu, Y.; Ke, X.; Qi, Y.; Li, W.; Zhu, P. Connexin26 gene (GJB2): prevalence of mutations in the Chinese population. *Journal of human genetics* **2002**, *47*, 0688-0690.

377. 373. Liu, Y.; Ke, X.; Yu, Q.; Ping, Z.; Read, A.P.; Newton, V. An analysis of audiograms and connexin-26 mutations among Chinese with non-syndromic hearing loss. *Journal of Audiological Medicine* **2002**, *11*, 129-136.

378. 374. Lopponen, T.; Dietz, A.; Vaisanen, M.L.; Valtonen, H.; Kosunen, A.; Hyvarinen, A.; Ignatius, J.; Lopponen, H. Homozygous M34T mutation of the GJB2 gene associates with an autosomal recessive nonsyndromic sensorineural hearing impairment in Finnish families. *Acta oto-laryngologica* **2012**, *132*, 862-873, doi:10.3109/00016489.2012.669498.

379. 375. Lucotte, G.; Bathelier, C.; Champenois, T. PCR test for diagnosis of the common GJB2 (connexin 26) 35delG mutation on dried blood spots and determination of the carrier frequency in France. *Mol Cell Probe* **2001**, *15*, 57-59, doi:10.1006/mcpr.2000.0335.

380. 376. Luo, J.; Bai, X.; Zhang, F.; Xiao, Y.; Gu, L.; Han, Y.; Fan, Z.; Li, J.; Xu, L.; Wang, H. Prevalence of Mutations in Deafness-Causing Genes in Cochlear Implanted Patients with Profound Nonsyndromic Sensorineural Hearing Loss in Shandong Province, China. *Annals of human genetics* **2017**, *81*, 258-266, doi:10.1111/ahg.12207.

381. 377. Matos, T.D.; Simoes-Teixeira, H.; Caria, H.; Goncalves, A.C.; Chora, J.; Correia Mdo, C.; Moura, C.; Rosa, H.; Monteiro, L.; O'Neill, A., et al. Spectrum and frequency of GJB2 mutations in a cohort of 264 Portuguese nonsyndromic sensorineural hearing loss patients. *International journal of audiology* **2013**, *52*, 466-471, doi:10.3109/14992027.2013.783719.

382. 378. Mendelsberg-Fishbein, P.; Márquez-Ávila, C.S.; García-Delgado, C.; SánchezBoiso, A.; Rodríguez-Espino, B.A.; Vázquez-Martínez, E.R.; Roque-Lee, G.; Ortiz-Rodríguez, S.; de los Ángeles Fierro-Evans, M.; Castillo-Castillo, S., et al. Importance of the diagnosis of protein connexin 26 mutations in the integral management of non-syndromic congenital deafness. *Boletin Medico del Hospital Infantil de Mexico* **2013**, *70*, 89-97.

383. 379. Minarik, G.; Ferak, V.; Ferakova, E.; Ficek, A.; Polakova, H.; Zatkova, A.; Kadasi, L. Mutations in the GJB-2 gene in patients with non-syndromic hearing loss from endogamous population of Roms (Gypsies). *European Journal of Human Genetics* **2002**, *10*, 245-245.

384. 380. MoctarEly Cheikh, M.; Riahi, Z.; El Hachmi, H.; Veten, F.; Meiloud, G.; Bonnet, C.; Abdelhak, S.; Errami, M.; Houmeida, A. Etiology and associated GJB2 mutations in Mauritanian children with non-syndromic hearing loss. *European Archives of Oto-Rhino-Laryngology* **2016**, *273*, 3693-3698.

385. 381. Mukherjee, S.; James, E.; Sukthankar, P.S.; Batra, R.B.; Nayyar, S.S.; Moorchung, N.; Venkatesh, M.D.; Sabarigirish, K. Connexin 26 mutations in congenital SNHL in Indian population. *Indian Journal of Otology* **2011**, *17*, 145, doi:10.4103/0971-7749.94491.

386. 382. Murgia, A.; Orzan, E.; Polli, R.; Martella, M.; Vinanzi, C.; Turato, R.; Zacchello, F. Clinical advantages from the molecular analysis of the Cx26 gene in preverbal sensorineural deafness. *Rivista Italiana di Pediatria* **2000**, *26*, 178-181.

387. 383. Naghavi, A.; Nishimura, C.; Kahrizi, K.; Riazalhosseini, Y.; Bazazzadegan, N.; Mohseni, M.; Smith, R.J.; Najmabadi, H. GJB2 mutations in Baluchi population. *J Genet* **2008**, *87*, 195-197, doi:10.1007/s12041-008-0031-5.

388. 384. Nagy, A.L.; Csaki, R.; Klem, J.; Rovo, L.; Toth, F.; Talosi, G.; Jori, J.; Kovacs, K.; Kiss, J.G. Minimally invasive genetic screen for GJB2 related deafness using dried blood spots. *International journal of pediatric otorhinolaryngology* **2010**, *74*, 75-81, doi:10.1016/j.ijporl.2009.10.021.

389. 385. Najmabadi, H.; Nishimura, C.; Kahrizi, K.; Riazalhosseini, Y.; Malekpour, M.; Daneshi, A.; Farhadi, M.; Mohseni, M.; Mahdieh, N.; Ebrahimi, A., et al. GJB2 mutations: passage through Iran. *American journal of medical genetics. Part A* **2005**, *133A*, 132-137, doi:10.1002/ajmg.a.30576.

390. 386. Neocleous, V.; Aspris, A.; Shahpenterian, V.; Nicolaou, V.; Panagi, C.; Ioannou, I.; Kyamides, Y.; Anastasiadou, V.; Phylactou, L.A. High frequency of 35delG GJB2 mutation and absence of del(GJB6-D13S1830) in Greek Cypriot patients with nonsyndromic hearing loss. *Genet Test* **2006**, *10*, 285-289, doi:10.1089/gte.2006.10.285.

391. 387. Niceta, M.; Fabiano, C.; Sammarco, P.; Piccione, M.; Antona, V.; Giuffre, M.; Corsello, G. Epidemiological study of nonsyndromic hearing loss in Sicilian newborns. *American journal of medical genetics. Part A* **2007**, *143A*, 1666-1670, doi:10.1002/ajmg.a.31836.

392. 388. Niepokoj, K.; Rygiel, A.M.; Jurczak, P.; Kujko, A.A.; Sniegorska, D.; Sawicka, J.; Grabarczyk, A.; Bal, J.; Wertheim-Tysarowska, K. Hearing impairment caused by mutations in two different genes responsible for nonsyndromic and syndromic hearing loss within a single family. *Journal of applied genetics* **2018**, *59*, 67-72, doi:10.1007/s13353-017-0416-3.

393. 389. Olafsson, I.; Hjaltadottir, G.; Cook, E.; Thornorisson, H.M.; Eiriksdottir, G.; Petersen, H. [Hereditary hearing impairment. Mutation analysis of connexin 26 and POU3F4 genes in Icelanders with nonsyndromic hearing impairment.]. *Laeknabladid* **2000**, *86*, 833-839.

394. 390. Padma, G.; Ramchander, P.V.; Nandur, U.V.; Padma, T. GJB2 and GJB6 gene mutations found in Indian probands with congenital hearing impairment. *J Genet* **2009**, *88*, 267-272, doi:10.1007/s12041-009-0039-5.

395. 391. Padma, G.; Ramchander, P.V.; Nandur, U.V.; Padma, T. A rare event of maternal UPD in a proband with congenital non-syndromic hearing impairment with homozygosity for gjb2 p.w24x mutation. *International Journal of Genetics and Molecular Biology* **2010**, *2*, 126-129.

396. 392. Pallares-Ruiz, N.; Blanchet, P.; Mondain, M.; Low-Hong, S.; Demaille, J.; Claustres, M.; Roux, A.F. Evaluation of dHPLC for CX26 mutation screening in patients from southern France with sensorineural deafness. *Genet Test* **2001**, *5*, 339-343, doi:10.1089/109065701753617507.

397. 393. Pampanos, A.; Economides, J.; Iliadou, V.; Neou, P.; Leotsakos, P.; Voyiatzis, N.; Eleftheriades, N.; Tsakanikos, M.; Antoniadi, T.; Hatzaki, A., et al. Prevalence of GJB2 mutations in prelingual deafness in the Greek population. *International journal of pediatric otorhinolaryngology* **2002**, *65*, 101-108, doi:10.1016/s0165-5876(02)00177-5.

398. 394. Pan, J.; Xu, P.; Tang, W.; Cui, Z.; Feng, M.; Wang, C. Mutation analysis of common GJB2, SCL26A4 and 12S rRNA genes among 380 deafness patients in northern China. *International journal of pediatric otorhinolaryngology* **2017**, *98*, 39-42, doi:10.1016/j.ijporl.2017.04.018.

399. 395. Piatto, V.B.; Moreira, O.A.V.; Da Silva, M.A.O.M.; Maniglia, J.V.; Pereira, M.C.; Sartorato, E.L. Correlation between audiometric data and the 35delG mutation in ten patients. *Brazilian journal of otorhinolaryngology* **2007**, *73*, 777-783.

400. 396. Posukh, O.L.; Zytsar, M.V.; Bady-Khoo, M.S.; Danilchenko, V.Y.; Maslova, E.A.; Barashkov, N.A.; Bondar, A.A.; Morozov, I.V.; Maximov, V.N.; Voevoda, M.I. Unique Mutational Spectrum of the GJB2 Gene and its Pathogenic Contribution to Deafness in Tuvinians (Southern Siberia, Russia): A High Prevalence of Rare Variant c.516G>C (p.Trp172Cys). *Genes (Basel)* **2019**, *10*, doi:10.3390/genes10060429.

401. 397. Prera, N.; Lohle, E.; Birkenhager, R. [Progressive hearing impairment with deletion in GJB2 gene despite normal newborn hearing screening]. *Laryngo- rhino- otologie* **2014**, *93*, 244-248, doi:10.1055/s-0033-1355343.

402. 398. Primignani, P.; Trotta, L.; Castorina, P.; Lalatta, F.; Sironi, F.; Radaelli, C.; Degiorgio, D.; Curcio, C.; Travi, M.; Ambrosetti, U., et al. Analysis of the GJB2 and GJB6 genes in Italian patients with nonsyndromic hearing loss: frequencies, novel mutations, genotypes, and degree of hearing loss. *Genet Test Mol Biomarkers* **2009**, *13*, 209-217, doi:10.1089/gtmb.2008.0086.

403. 399. Putcha, G.V.; Bejjani, B.A.; Bleoo, S.; Booker, J.K.; Carey, J.C.; Carson, N.; Das, S.; Dempsey, M.A.; Gastier-Foster, J.M.; Greinwald, J.H., Jr., et al. A multicenter study of the frequency and distribution of GJB2 and GJB6 mutations in a large North American cohort. *Genet Med* **2007**, *9*, 413-426, doi:10.1097GIM.0b013e3180a03276.

404. 400. Qu, C.; Sun, X.; Shi, Y.; Gong, A.; Liang, S.; Zhao, M.; Chen, Y.; Liang, F. Microarray-based mutation detection of pediatric sporadic nonsyndromic hearing loss in China. *International journal of pediatric otorhinolaryngology* **2012**, *76*, 235-239, doi:10.1016/j.ijporl.2011.11.009.

405. 401. Ramchander, P.V.; Nandur, V.U.; Dwarakanath, K.; Vishnupriya, S.; Padma, T. Study of families of nonsyndromic hearing impairment segregating with mutations in Cx26 gene. *Indian Journal of Human Genetics* **2004**, *10*, 58-64.

406. 402. Ratbi, I.; Hajji, S.; Ouldim, K.; Aboussair, N.; Feldmann, D.; Sefiani, A. [The mutation 35delG of the gene of the connexin 26 is a frequent cause of autosomal-recessive non-syndromic hearing loss in Morocco]. *Archives de pediatrie : organe officiel de la Societe francaise de pediatrie* **2007**, *14*, 450-453, doi:10.1016/j.arcped.2006.12.004.

407. 403. Sansovic, I.; Knezevic, J.; Matijevic, T.; Balen, S.; Barisic, I.; Pavelic, J. Prevalence of the 35delG mutation in the GJB2 gene of patients with nonsyndromic hearing loss from Croatia. *Genet Test* **2005**, *9*, 297-300, doi:10.1089/gte.2005.9.297.

408. 404. Sansovic, I.; Knezevic, J.; Musani, V.; Seeman, P.; Barisic, I.; Pavelic, J. GJB2 mutations in patients with nonsyndromic hearing loss from Croatia. *Genet Test Mol Biomarkers* **2009**, *13*, 693-699, doi:10.1089/gtmb.2009.0073.

409. 405. Sasanfar, R.; Tolouei, A.; Hoseinipour, A.; Farhud, D.D.; Dolati, M.; Rad, L.H.; Zohour, M.M.; Ghadami, M.; Pour-Jafari, H.; Chaleshtori, M.H. Frequency of a very rare 35delG mutation in two ethnic groups of Iranian populations. *Iranian Journal of Public Health* **2004**, *33*, 26-30.

410. 406. Seeman, P.; Malikova, M.; Raskova, D.; Bendova, O.; Groh, D.; Kubalkova, M.; Sakmaryova, I.; Seemanova, E.; Kabelka, Z. Spectrum and frequencies of mutations in the GJB2 (Cx26) gene among 156 Czech patients with pre-lingual deafness. *Clinical genetics* **2004**, *66*, 152-157, doi:10.1111/j.1399-0004.2004.00283.x.

411. 407. Shaikh, H.; Waryah, A.M.; Narsani, A.K.; Iqbal, M.; Shahzad, M.; Waryah, Y.M.; Shaikh, N.; Mahmood, A. Genetic Testing of Non-familial Deaf Patients for CIB2 and GJB2 Mutations: Phenotype and Genetic Counselling. *Biochem Genet* **2017**, *55*, 410-420, doi:10.1007/s10528-017-9828-3.

412. 408. Shalev, S.A.; Hujirat, Y. Maternal origin of a de novo mutation of the connexin 26 gene resulting in recessive nonsyndromic deafness. *American journal of medical genetics. Part A* **2004**, *124A*, 411-412, doi:10.1002/ajmg.a.20406.

413. 409. Silan, F.; Guclu, O.; Kadioglu, L.E.; Silan, C.; Atik, S.; Uludag, A.; Demiray, A.; Ozdemir, O.; Derekoy, F.S. GJB2 35delG and Mitochondrial A1555G Mutations and Etiology of Deafness at the Gelibolu School for the Deaf in Turkey. *Journal of International Advanced Otology* **2011**, *7*, 361-371.

414. 410. Snoeckx, R.L.; Huygen, P.L.; Feldmann, D.; Marlin, S.; Denoyelle, F.; Waligora, J.; Mueller-Malesinska, M.; Pollak, A.; Ploski, R.; Murgia, A., et al. GJB2 mutations and degree of hearing loss: a multicenter study. *American journal of human genetics* **2005**, *77*, 945-957, doi:10.1086/497996.

415. 411. Sobe, T.; Vreugde, S.; Shahin, H.; Berlin, M.; Davis, N.; Kanaan, M.; Yaron, Y.; Orr-Urtreger, A.; Frydman, M.; Shohat, M., et al. The prevalence and expression of inherited connexin 26 mutations associated with nonsyndromic hearing loss in the Israeli population. *Human genetics* **2000**, *106*, 50-57, doi:10.1007/s004390051009.

416. 412. Stefanovska, E.S.; Momirovska, A.; Cakar, M.; Efremov, G.D. Gjb2 Mutations in Non Syndromic Hearing Loss in the Republic of Macedonia. *Balkan Journal of Medical Genetics* **2009**, *12*, 11-16, doi:10.2478/v10034-010-0004-x.

417. 413. Šterna, O.; Proņina, N.; Grīnfelde, I.; Kušķe, S.; Krūmiņa, A.; Lugovska, R.; Pētersons, A. Spectrum and Frequency of the GJB2 Gene Mutations Among Latvian Patients with Prelingual Nonsyndromic Hearing Loss. *Proceedings of the Latvian Academy of Sciences. Section B. Natural, Exact, and Applied Sciences.* **2009**, *63*, 198-203, doi:10.2478/v10046-009-0031-8.

418. 414. Stevenson, V.A.; Ito, M.; Milunsky, J.M. Connexin-30 deletion analysis in connexin-26 heterozygotes. *Genet Test* **2003**, *7*, 151-154, doi:10.1089/109065703322146867.

419. 415. Stinckens, C.; Kremer, H.; van Wijk, E.; Hoefsloot, L.H.; Huygen, P.L.; Standaert, L.; Fryns, J.P.; Cremers, C.W. Longitudinal phenotypic analysis in patients with connexin 26 (GJB2) (DFNB1) and connexin 30 (GJB6) mutations. *The Annals of otology, rhinology, and laryngology* **2004**, *113*, 587-593, doi:10.1177/000348940411300714.

420. 416. Sugata, A.; Fukushima, K.; Sugata, K.; Fukuda, S.; Kimura, N.; Gunduz, M.; Kasai, N.; Usami, S.; Smith, R.J.; Nishizaki, K. High-throughput screening for GJB2 mutations--its clinical application to genetic testing in prelingual deafness screening for GJB2 mutations. *Auris Nasus Larynx* **2002**, *29*, 231-239, doi:10.1016/s0385-8146(02)00014-7.

421. 417. Tahmasebi, P.; Chaleshtori, M.H.; Abdollahnejad, F.; Alavi, Z.; Sadeghian, L.; Talebi, F.; Mohammadi-Asl, J.; Saki, N.; Nezhad, S.R.K.; Tabatabaiefar, M.A. Frequency of Gjb2 Mutations in Families with Autosomal Recessive Non-Syndromic Hearing Loss in Khuzestan Province. *Genetika-Belgrade* **2018**, *50*, 837-846, doi:10.2298/Gensr1803837t.

422. 418. Talbi, S.; Bonnet, C.; Boudjenah, F.; Mansouri, M.T.; Petit, C.; Ammar Khodja, F. The spectrum of GJB2 gene mutations in Algerian families with nonsyndromic hearing loss from Sahara and Kabylie regions. *International journal of pediatric otorhinolaryngology* **2019**, *124*, 157-160, doi:10.1016/j.ijporl.2019.05.036.

423. 419. Talbi, S.; Bonnet, C.; Riahi, Z.; Boudjenah, F.; Dahmani, M.; Hardelin, J.P.; Wong Jun Tai, F.; Louha, M.; Ammar-Khodja, F.; Petit, C. Genetic heterogeneity of congenital hearing impairment in Algerians from the Ghardaia province. *International journal of pediatric otorhinolaryngology* **2018**, *112*, 1-5, doi:10.1016/j.ijporl.2018.06.012.

424. 420. Tekin, D.; Yan, D.; Bademci, G.; Feng, Y.; Guo, S.; Foster, J., 2nd; Blanton, S.; Tekin, M.; Liu, X. A next-generation sequencing gene panel (MiamiOtoGenes) for comprehensive analysis of deafness genes. *Hearing research* **2016**, *333*, 179-184, doi:10.1016/j.heares.2016.01.018.

425. 421. Tóth, T.; Kupka, S.; Blin, N.; Pfister, M.; Sziklai, I. Frequency of the Connexin26/35delG mutation and its characteristic phenotype in patients with hearing impairment and controls in Northeastern Hungary. *Orvosi hetilap* **2002**, *143*, 2285-2289.

426. 422. Totolin, M.; Boborelu, G.; Ioniţă, E.; Mitroi, M. Prevalence of 35delG/GJB2 mutation in children with congenital nonsyndromic sensorineural hearing loss in Oltenia (South-Western Romania). *Archives of the Balkan Medical Union* **2013**, *48*, 177-181.

427. 423. Trabelsi, M.; Bahri, W.; Habibi, M.; Zainine, R.; Maazoul, F.; Ghazi, B.; Chaabouni, H.; Mrad, R. GJB2 and GJB6 screening in Tunisian patients with autosomal recessive deafness. *International journal of pediatric otorhinolaryngology* **2013**, *77*, 714-716, doi:10.1016/j.ijporl.2013.01.024.

428. 424. Utrera, R.; Ridaura, V.; Rodriguez, Y.; Rojas, M.J.; Mago, L.; Angeli, S.; Henriquez, O. Detection of the 35delG/GJB2 and del(GJB6-D13S1830) mutations in Venezuelan patients with autosomal recessive nonsyndromic hearing loss. *Genet Test* **2007**, *11*, 347-352, doi:10.1089/gte.2006.0526.

429. 425. Vona, B.; Muller, T.; Nanda, I.; Neuner, C.; Hofrichter, M.A.; Schroder, J.; Bartsch, O.; Lassig, A.; Keilmann, A.; Schraven, S., et al. Targeted next-generation sequencing of deafness genes in hearing-impaired individuals uncovers informative mutations. *Genet Med* **2014**, *16*, 945-953, doi:10.1038/gim.2014.65.

430. 426. Wang, C.; Cai, X.; Zou, G.; Tie, L.; Li, Y. [Analysis on GJB2 gene mutations with nonsyndromic hearing impairment in Kazak patients of Xinjiang]. *Lin chuang er bi yan hou tou jing wai ke za zhi = Journal of clinical otorhinolaryngology, head, and neck surgery* **2014**, *28*, 676-679.

431. 427. Wiszniewski, W.; Sobieszczanska-Radoszewska, L.; Nowakowska-Szyrwinska, E.; Obersztyn, E.; Bal, J. High frequency of GJB2 gene mutations in Polish patients with prelingual nonsyndromic deafness. *Genet Test* **2001**, *5*, 147-148, doi:10.1089/109065701753145637.

432. 428. Wu, C.C.; Lu, Y.C.; Chen, P.J.; Liu, A.Y.; Hwu, W.L.; Hsu, C.J. Application of SNaPshot multiplex assays for simultaneous multigene mutation screening in patients with idiopathic sensorineural hearing impairment. *The Laryngoscope* **2009**, *119*, 2411-2416, doi:10.1002/lary.20621.

433. 429. Wu, H.; Feng, Y.; Jiang, L.; Pan, Q.; Liu, Y.; Liu, C.; He, C.; Chen, H.; Liu, X.; Hu, C., et al. Application of a New Genetic Deafness Microarray for Detecting Mutations in the Deaf in China. *PloS one* **2016**, *11*, e0151909, doi:10.1371/journal.pone.0151909.

434. 430. Wu, L.H.; Li, R.Y.; Ye, Q.; Lin, Q.M.; Que, X.M.; Xiao, Y. Genetic mutations of hotspots in patients with nonsyndromic deafness in Fujian, China. *International Journal of Clinical and Experimental Pathology* **2017**, *10*, 7067-7074.

435. 431. Wu, X.; Gao, X.; Han, P.; Zhou, Y. Identification of causative variants in patients with non-syndromic hearing loss in the Minnan region, China by targeted next-generation sequencing. *Acta oto-laryngologica* **2019**, *139*, 243-250, doi:10.1080/00016489.2018.1552015.

436. 432. Xia, H.; Huang, X.; Xu, H.; Zhou, Y.A.; Gong, L.; Yang, Z.; Lv, J.; Deng, H. GJB2 c.235delC variant associated with autosomal recessive nonsyndromic hearing loss and auditory neuropathy spectrum disorder. *Genet Mol Biol* **2019**, *42*, 48-51, doi:10.1590/1678-4685-gmb-2017-0318.

437. 433. Xiang, Y.B.; Tang, S.H.; Li, H.Z.; Xu, C.Y.; Chen, C.; Xu, Y.Z.; Ding, L.R.; Xu, X.Q. Mutation analysis of common deafness-causing genes among 506 patients with nonsyndromic hearing loss from Wenzhou city, China. *International journal of pediatric otorhinolaryngology* **2019**, *122*, 185-190, doi:10.1016/j.ijporl.2019.04.024.

438. 434. Xiao, Z.A.; Xie, D.H. GJB2 (Cx26) gene mutations in Chinese patients with congenital sensorineural deafness and a report of one novel mutation. *Chinese medical journal* **2004**, *117*, 1797-1801.

439. 435. Xiong, Y.; Zhong, M.; Chen, J.; Yan, Y.L.; Lin, X.F.; Li, X. Effect of GJB2 235delC and 30-35delG genetic polymorphisms on risk of congenital deafness in a Chinese population. *Genetics and molecular research : GMR* **2017**, *16*, doi:10.4238/gmr16019165.

440. 436. Xu, C.; Zhang, H.; Zhang, Y.; Zhao, S.; Geng, X.; Shan, Y.; Shan, X. [Mutations analysis in a pedigree with maternally inherited sensorineural hearing loss]. *Zhonghua yi xue yi chuan xue za zhi = Zhonghua yixue yichuanxue zazhi = Chinese journal of medical genetics* **2005**, *22*, 125-128.

441. 437. Yan, D.; Xiang, G.; Chai, X.; Qing, J.; Shang, H.; Zou, B.; Mittal, R.; Shen, J.; Smith, R.J.; Fan, Y.S., et al. Screening of deafness-causing DNA variants that are common in patients of European ancestry using a microarray-based approach. *PloS one* **2017**, *12*, e0169219, doi:10.1371/journal.pone.0169219.

442. 438. Yang, J.J.; Huang, S.H.; Chou, K.H.; Liao, P.J.; Su, C.C.; Li, S.Y. Identification of mutations in members of the connexin gene family as a cause of nonsyndromic deafness in Taiwan. *Audiology & neuro-otology* **2007**, *12*, 198-208, doi:10.1159/000099024.

443. 439. Yilmaz, A.; Menevse, S.; Bayazit, Y.; Karamert, R.; Ergin, V.; Menevse, A. Two novel missense mutations in the connexin 26 gene in Turkish patients with nonsyndromic hearing loss. *Biochem Genet* **2010**, *48*, 248-256, doi:10.1007/s10528-009-9314-7.

444. 440. Yu, F.; Han, D.Y.; Dai, P.; Kang, D.Y.; Zhang, X.; Liu, X.; Zhu, Q.W.; Yuan, Y.Y.; Sun, Q.; Xue, D.D., et al. [Mutation of GJB2 gene in nonsyndromic hearing impairment patients: analysis of 1190 cases]. *Zhonghua yi xue za zhi* **2007**, *87*, 2814-2819.

445. 441. Zhang, F.; Xiao, Y.; Xu, L.; Zhang, X.; Zhang, G.; Li, J.; Lv, H.; Bai, X.; Wang, H. Mutation Analysis of the Common Deafness Genes in Patients with Nonsyndromic Hearing Loss in Linyi by SNPscan Assay. *Biomed Res Int* **2016**, *2016*, 1302914, doi:10.1155/2016/1302914.

446. 442. Zhang, J.; Wang, Z.; Dai, W.; Zeng, Y.; Li, H. GJB2 allele variants and the associated audiologic features identified in Chinese patients with less severe idiopathic hearing loss. *Genet Test Mol Biomarkers* **2011**, *15*, 313-318, doi:10.1089/gtmb.2010.0182.

447. 443. Zhu, J.; Cao, Q.; Zhang, N.; Ge, J.; Sun, D.; Feng, Q. A study of deafness-related genetic mutations as a basis for strategies to prevent hereditary hearing loss in Hebei, China. *Intractable Rare Dis Res* **2015**, *4*, 131-138, doi:10.5582/irdr.2015.01018.

448. 444. Zoll, B.; Petersen, L.; Lange, K.; Gabriel, P.; Kiese-Himmel, C.; Rausch, P.; Berger, J.; Pasche, B.; Meins, M.; Gross, M., et al. Evaluation of Cx26/GJB2 in German hearing impaired persons: mutation spectrum and detection of disequilibrium between M34T (c.101T>C) and -493del10. *Human mutation* **2003**, *21*, 98, doi:10.1002/humu.9098.

449. 445. Denoyelle, F.; Marlin, S.; Weil, D.; Moatti, L.; Chauvin, P.; Garabedian, E.N.; Petit, C. Clinical features of the prevalent form of childhood deafness, DFNB1, due to a connexin-26 gene defect: implications for genetic counselling. *Lancet* **1999**, *353*, 1298-1303, doi:Doi 10.1016/S0140-6736(98)11071-1.

450.
